# RNA polymerase II clusters form in line with surface condensation on regulatory chromatin

**DOI:** 10.15252/msb.202110272

**Published:** 2021-09-27

**Authors:** Agnieszka Pancholi, Tim Klingberg, Weichun Zhang, Roshan Prizak, Irina Mamontova, Amra Noa, Marcel Sobucki, Andrei Yu Kobitski, Gerd Ulrich Nienhaus, Vasily Zaburdaev, Lennart Hilbert

**Affiliations:** ^1^ Zoological Institute Department of Systems Biology and Bioinformatics Karlsruhe Institute of Technology Karlsruhe Germany; ^2^ Institute of Biological and Chemical Systems—Biological Information Processing Karlsruhe Institute of Technology Eggenstein‐Leopoldshafen Germany; ^3^ Department of Biology Friedrich‐Alexander‐Universität Erlangen‐Nürnberg Erlangen Germany; ^4^ Max‐Planck‐Zentrum für Physik und Medizin Erlangen Germany; ^5^ Institute of Applied Physics Karlsruhe Institute of Technology Karlsruhe Germany; ^6^ Institute of Nanotechnology Karlsruhe Institute of Technology Eggenstein‐Leopoldshafen Germany; ^7^ Department of Physics University of Illinois at Urbana‐Champaign Urbana IL USA

**Keywords:** active matter, gene regulation, nuclear organization, super‐resolution microscopy, transcriptional condensates, Chromatin, Transcription & Genomics

## Abstract

It is essential for cells to control which genes are transcribed into RNA. In eukaryotes, two major control points are recruitment of RNA polymerase II (Pol II) into a paused state, and subsequent pause release toward transcription. Pol II recruitment and pause release occur in association with macromolecular clusters, which were proposed to be formed by a liquid–liquid phase separation mechanism. How such a phase separation mechanism relates to the interaction of Pol II with DNA during recruitment and transcription, however, remains poorly understood. Here, we use live and super‐resolution microscopy in zebrafish embryos to reveal Pol II clusters with a large variety of shapes, which can be explained by a theoretical model in which regulatory chromatin regions provide surfaces for liquid‐phase condensation at concentrations that are too low for canonical liquid–liquid phase separation. Model simulations and chemical perturbation experiments indicate that recruited Pol II contributes to the formation of these surface‐associated condensates, whereas elongating Pol II is excluded from these condensates and thereby drives their unfolding.

## Graphical abstract

Recruited RNA polymerase II forms clusters via surface condensation on regulatory chromatin. These clusters unfold due to the exclusion of elongating polymerase from condensates.

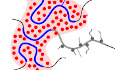

## Introduction

**Figure 1:**
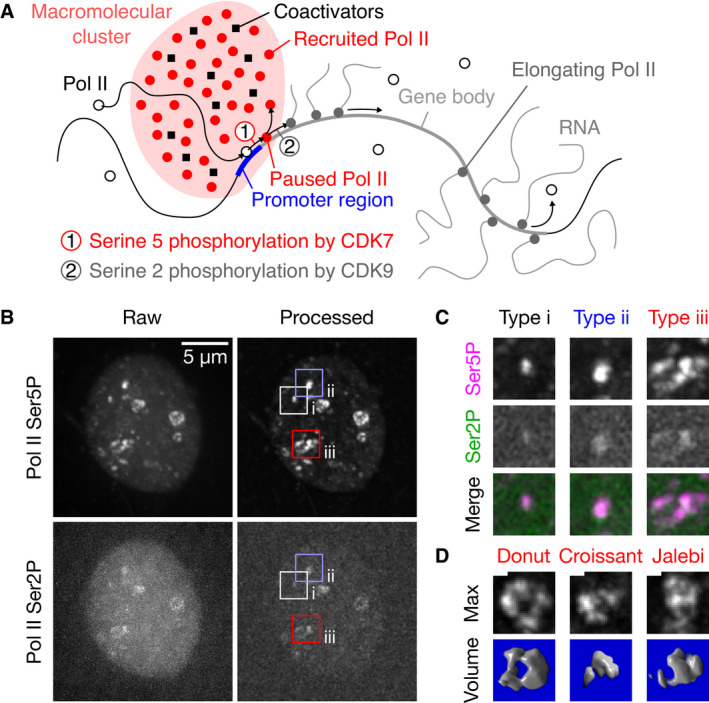
Phosphorylation‐specific detection of RNA polymerase II reveals clusters displaying a variety of morphologies Sketch of the recruitment and pause release of RNA polymerase II (Pol II) in the context of macromolecular clusters.Representative maximum‐intensity projection of a nucleus in a live zebrafish embryo (sphere stage), where Pol II was detected via fluorescently labeled antigen‐binding fragments (Fab) specific against Ser5 and Ser2 phosphorylation of the C‐terminal domain heptad repeat (Pol II Ser5P, Pol II Ser2P). Pol II Ser5P clusters representing the different apparent types of morphologies are marked. Single time point z‐stacks were recorded using an instantaneous Structured Illumination Microscope (instant‐SIM), raw data were processed by local background subtraction (both channels) and smoothed (Pol II Ser2P only).Detail views of the clusters of the apparent morphology types i–iii, as marked in panel (B). The detail views are 2.7 μm across.Examples of the varied morphologies of type iii clusters, shown as maximum‐intensity projections and corresponding volume renderings of the processed Pol II Ser5P signal. Morphologies are named by similarity to patisserie and candy items. Scale bar: 500 nm. 3D renderings: ImageJ Volume Viewer plugin.

Eukaryotic cells have an extensive library of genetic DNA sequences at their disposal, but selectively transcribe only a small subset of this genetic information into RNA transcripts at any given point in time. For the vast majority of genes whose transcription is controlled, the synthesis of RNA transcripts is carried out by the multi‐protein complex RNA polymerase II (Pol II). Two major points at which transcription by Pol II is controlled are initiation and pause release (Bartman *et al*, 2019). During initiation, Pol II engages with the promoter region, a sequence that is located upstream of an actual gene and integrates many regulatory influences, and starts synthesizing the RNA transcript (Fig 1A). After proceeding for 20–60 base pairs along the DNA sequence, initiated Pol II complexes enter a state of promoter‐proximal pausing (Adelman & Lis, 2012). The rates of Pol II initiation and subsequent release from the paused state are under cellular control and can differ between genes and change in response to stimuli (Gressel *et al*, 2017; Bartman *et al*, 2019). Initiated as well as paused Pol II readily detaches from DNA, only the transition into processive elongation past the paused state leads to stable engagement (Stasevich *et al*, 2014; Steurer *et al*, 2018; Forero‐Quintero *et al*, 2021). For genes with a pause release rate similar to or greater than the rate of initiation, Pol II proceeds into proper elongation with negligible retention in the promoter‐proximal position (Chen *et al*, 2013). In contrast, when pause release is slower than initiation, Pol II remains in the promoter‐proximal position, thus entering the so‐called poised state. Genes that exhibit poising remain ready for induction, enabling, for example, an extensive transcriptional response to heat shock (Gressel *et al*, 2019) and, potentially, the trans‐differentiation of neuronal types (Ferrai *et al*, 2017). In early embryonic development, some genes are also poised—supposedly in preparation for subsequent expression during cell type specification (Chen *et al*, 2013; Ghavi‐Helm *et al*, 2014).

A complementary perspective on Pol II initiation and pause release considers changes in the localization of Pol II (Fig 1A). It has been proposed that transcription occurs in static factories, containing several Pol II complexes and several genes in a shared local context (Iborra *et al*, 1996; Cook, 1999; Ferrai *et al*, 2010; Papantonis & Cook, 2013). This initial transcription factory picture is refined based on live‐cell microscopy, showing dynamic macromolecular clusters that are enriched in Pol II and provide platforms for the initiation of transcript elongation (Cissé *et al*, 2013; Cho *et al*, 2016; Wei *et al*, 2020a). These clusters are supposedly formed by mechanisms related to liquid–liquid phase separation (LLPS) and support the co‐association of activating factors such as the protein Mediator or activity‐inducing chromatin remodelers (Cho *et al*, 2018; Chong *et al*, 2018; Sabari *et al*, 2018; Li *et al*, 2020). Post‐translational modifications that occur during Pol II recruitment have been found to control the association of Pol II with such clusters. Specifically, Pol II initiation proceeds in conjunction with phosphorylation of serine 5 (Ser5P) of the Pol II carboxy‐terminal domain (CTD) YSPTSPS repeat array by cyclin‐dependent kinase 7 (CDK7). This Ser5P mark drives the association of Pol II with liquid‐phase droplets *in vitro* or macromolecular clusters *in vivo*, which are enriched in coactivators such as cyclin‐dependent kinase 9 (CDK9) (Lu *et al*, 2018; Guo *et al*, 2020). Pol II Ser5P that remains engaged after initiation, in turn, is phosphorylated by CDK9 at serine 2 (Ser2P) of the Pol II CTD‐repeat, a modification that is essential to enable pause release and subsequent elongation (Ahn *et al*, 2004). The newly deposited Ser2P mark also abolishes the affinity of Pol II for the Pol II‐enriched clusters (Lu *et al*, 2018), allowing Pol II relocation toward nuclear speckles associated with further RNA processing (Hu *et al*, 2009; Guo *et al*, 2019). Pol II can also be released from DNA before proceeding into proper transcript elongation, while retaining the Ser5P mark (Stasevich *et al*, 2014; Steurer *et al*, 2018; Forero‐Quintero *et al*, 2021). The released Pol II Ser5P thus maintains its affinity for macromolecular clusters and, together with initiated Pol II, can be classified as recruited.

While LLPS was suggested as part of the mechanism for the formation of Pol II‐enriched clusters, these clusters exhibit complex morphologies that deviate markedly from the round, droplet‐like shapes typical of canonical LLPS (Eskiw *et al*, 2008; Brangwynne *et al*, 2009; Cho *et al*, 2018; McSwiggen *et al*, 2019). Recent live‐cell microscopy results suggest that the progression of Pol II through recruitment and pause release affects the internal organization of Pol II‐enriched macromolecular clusters (Li *et al*, 2019, 2020; Forero‐Quintero *et al*, 2021). Such reorganization processes might contribute to the complex morphologies exhibited by Pol II‐enriched clusters. Here, we analyze how Pol II recruitment and pause release are related to cluster morphology by a combination of live‐cell and super‐resolution microscopy in zebrafish embryos with lattice simulations of liquid‐phase condensation on polymer surfaces. We observe that clusters exhibit various types of morphologies that are associated with different levels of Pol II Ser5P and Pol II Ser2P. Our lattice simulations reproduce this observation, explaining cluster formation via condensation of a liquid phase that is enriched in recruited Pol II on surfaces provided by regulatory genomic regions, and cluster unfolding via the exclusion of elongating Pol II from this liquid phase. The causal relevance of Pol II phosphorylation is supported by chemical perturbation of Pol II recruitment and pause release, which induces changes in cluster morphology and cluster number that are in line with results from our lattice simulations. In combination with previous work on Pol II liquid‐phase behavior (Cho *et al*, 2018; Sabari *et al*, 2018) and studies showing surface condensation of transcription factors on DNA *in vitro* (preprint: Morin *et al*, 2020; Quail *et al*, 2021; preprint: Renger *et al*, 2021), our findings in zebrafish, an embryonic model system, suggest that a similar surface condensation on regulatory chromatin might occur *in vivo* as well.

## Results

### Recruited RNA polymerase II occurs in clusters exhibiting different types of morphologies

To study Pol II‐enriched clusters, we used zebrafish embryos in the pluripotent stage of development (sphere) as an experimental model system. Zebrafish embryos provide the context of a normally developing vertebrate and are amenable to study by light microscopy. Our previous work demonstrated that fluorescently labeled antigen‐binding fragments (Fab) of antibodies against post‐translational modifications do not interfere with the normal development in an obvious manner and provide good sensitivity as well as time resolution in zebrafish embryos (Sato *et al*, 2019; Hilbert *et al*, 2021). To assess Pol II specifically in the recruited and elongating states, we injected embryos with antigen‐binding fragments against Pol II Ser5P and Pol II Ser2P, respectively. We acquired microscopy images from live embryos using an instantaneous Structured Illumination Microscope (instant‐SIM), which provides approximately twofold increased resolution in all three spatial dimensions relative to conventional confocal microscopy (York *et al*, 2013).

In our microscopy images, the Pol II Ser5P channel (recruited Pol II) revealed prominent clusters with a rich array of morphologies (Fig 1B). These clusters were long‐lived, persisting for more than 10 min (Appendix Fig S1). The observation of long‐lived clusters is in line with results from another model of pluripotency, mouse embryonic stem cells (mESCs) (Cho *et al*, 2018). Based on the shapes of the clusters observed in the Pol II Ser5P channel, we gained the impression that clusters mostly occur in three distinct morphology types (Fig 1C). These morphology types seem to correlate with different levels of Pol II Ser5P and Pol II Ser2P signal (elongating Pol II). (Note that the Pol II CTD YSPTSPS motif is repeated 52 times per Pol II complex in zebrafish, implying that (i) fluorescence intensity is not necessarily directly proportional to molecule numbers, and (ii) signal is amplified, so that spots might correspond to single genes, or even single polymerases.) Type i clusters are small, appear as dots in the Pol II Ser5P channel, and exhibit low Pol II Ser2P signal. Type ii clusters are larger, relatively compact in the Pol II Ser5P channel, and also exhibit low Pol II Ser2P signal. Type iii clusters are also larger, appear unfolded in the Pol II Ser5P channel, and have relatively high Pol II Ser2P signal. The unfolded type iii clusters show especially complex shapes with a large morphological variety. Illustrative examples include shapes resembling donuts (a ring with a hole in the center), croissants (a crescent shape), or Jalebi sweets (segments running across each other; Fig 1D). Taken together, recruited Pol II forms distinct, long‐lived clusters with a rich array of morphologies that appear to vary with the levels of Pol II Ser2 and Ser5 phosphorylation present at a given cluster.

### Recruited Pol II is associated with large clusters, whereas elongating Pol II is located at the margins of unfolded large clusters

**Figure 2:**
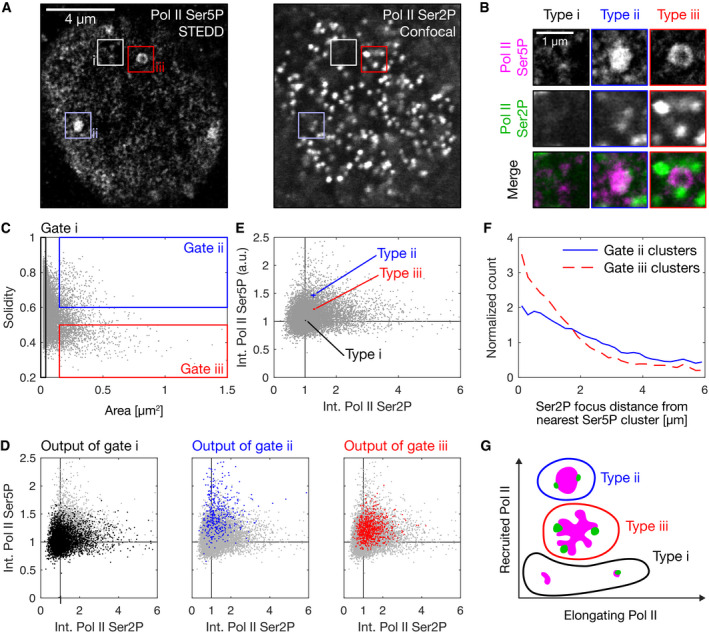
Super‐resolution microscopy reveals types of cluster morphologies correlating with levels of recruited and elongating RNA polymerase II Representative nuclear mid‐section obtained by STEDD super‐resolution microscopy from a fixed sphere‐stage zebrafish embryo. Pol II Ser5P intensity distributions were obtained by STEDD microscopy, Pol II Ser2P intensity distributions by regular confocal microscopy from the same focal plane. Pol II Ser5P clusters with typical morphologies i–iii are marked.Detail views of the marked clusters, representing the typical morphologies i–iii.Area and solidity of individual clusters, with gate regions for the typical morphologies i–iii. Clusters were segmented based on Pol II Ser5P intensity, data obtained from a total of 52 mid‐nuclear sections from two different samples.The Pol II Ser5P and Pol II Ser2P intensities (mean intensity across all pixels inside a given cluster's segmentation mask) of the clusters in the gates i–iii are plotted in color over the entire ungated cluster population (light gray). Mean Pol II Ser5P and Pol II Ser2P intensities were scaled by the median value for each nucleus, then pooled.The median of the Pol II Ser5P and Pol II Ser2P levels of the gated clusters in panel D is plotted over the ungated population of clusters. Each cluster type is plotted with 95% bootstrapped confidence intervals in Pol II Ser5P and Pol II Ser2P direction (10,000 resamples).Analysis of the placement of Pol II Ser2P spots relative to Pol II Ser5P clusters of type ii or type iii (spots segmented based on Pol II Ser2P channel). Lines represent histograms of the distances to the nearest surface of a Pol II Ser5P cluster. The Euclidean metric was used to calculate distance of Ser2P foci gated pixels from nearest cluster. To display relative enrichment against random placement, histogram bin counts are normalized by counts for ungated pixel distances, then scaled by the mean across bins.Sketch of apparent morphology types of Pol II Ser5P clusters, placed by their levels of elongating and recruited Pol II.

To systematically characterize cluster morphologies and their relation to Pol II recruitment and elongation, we assessed clusters by super‐resolution microscopy in fixed embryos. Specifically, we applied stimulated emission double depletion (STEDD) microscopy, which significantly reduces background from low‐frequency contributions and out‐of‐focus light relative to conventional stimulated emission depletion (STED) microscopy (Gao & Nienhaus, 2017; Gao *et al*, 2017; Zhang *et al*, 2019). Here, we super‐resolved the Pol II Ser5P distribution and acquired the level of Pol II Ser2P in a second color channel in the same focal plane by regular confocal microscopy. The Pol II Ser5P channel revealed the same apparent cluster morphologies that were seen in our live imaging data (Fig 2A and B). The improved signal‐to‐noise ratio and better separation of color channels relative to our live imaging data revealed an additional detail: Pol II Ser2P signal occurred in a pattern of many small spots that is present throughout the nucleus, while the elevated Pol II Ser2P signal associated with type iii clusters was located directly adjacent to, but did not strongly overlap with the Pol II Ser5P signal (Fig 2B). To more comprehensively assess cluster morphology, we extracted individual clusters and characterized their morphologies by size (area) and compactness (solidity) using an automated analysis pipeline (Fig 2C, Appendix Fig S2A and B). Based on area and solidity, we gated the clusters into extreme examples of the morphology types i–iii (Fig 2C). The gated sets of clusters exhibited systematic differences in Pol II Ser2P and Pol II Ser5P intensities: Small clusters (type i) had low Pol II Ser5P and Pol II Ser2P levels; large and round clusters (type ii) had the highest levels of Pol II Ser5P and high levels of Pol II Ser2P; large and dispersed clusters (type iii) had intermediate levels of Pol II Ser5P and high levels of Pol II Ser2P (Fig 2D and E). Taken together, these results imply that high levels of Pol II Ser5P are associated with the large cluster types ii and iii.

Visual inspection of our STEDD micrographs further suggests that Pol II Ser2P is positioned relative to Pol II Ser5P in an intricate pattern, where their signals are separated but in close neighborhood of each other (Fig 2B). In particular, a comprehensive analysis shows that Pol II Ser2P clusters are positioned next to the margins of Pol II Ser5P clusters of type iii (Fig 2F). This arrangement is suggestive of a scenario where Pol II Ser5P is associated with a convergent force that establishes compact clusters and Pol II Ser2P is associated with an unfolding force that acts at the margins of a given cluster.

To rule out that conclusions from the analysis of the STEDD microscopy data are affected by technical artefacts, we carried out several control experiments. All results obtained by the analysis of STEDD images were reproduced, albeit at lower resolution, in an analysis of our live imaging data (Appendix Fig S3A–C). Fixation of Fab‐injected embryos allowed instant‐SIM microscopy with improved signal‐to‐noise ratio and temporal separation of color channels, thereby revealing a pattern of Pol II Ser2P spots placed adjacent to Pol II Ser5P clusters (Appendix Fig S3D) that resembles the pattern seen in our STEDD micrographs (Fig 2A and B). The data obtained from these fixed embryos also reproduced the results obtained by the gating‐based analysis of STEDD data (Appendix Fig S3E–G). To verify the specificity of the Pol II Ser2P immunofluorescence detection, we also recorded data from embryos fixed at the oblong stage of development. The oblong stage precedes the sphere stage, and in the minutes directly following cell division, nuclei exhibit prominent clusters of elongating Pol II that are associated with microRNA miR‐430 transcription (Chan *et al*, 2019; Hadzhiev *et al*, 2019; Hilbert *et al*, 2021). Staining with the same primary antibodies used to label samples for STEDD microscopy, we indeed observed the expected prominent clusters of Pol II Ser5P and Pol II Ser2P signal (Appendix Fig S4). Note that, in line with a conversion from morphology type ii to morphology type iii, the shape of these prominent clusters seen in the Pol II Ser5P channel was more unfolded for higher levels of the Pol II Ser2P signal (Appendix Fig S4). The specificity of the antibody used to label Pol II Ser2P for STEDD microscopy was further confirmed by treatment of whole embryos with the transcription inhibitor flavopiridol, which indeed largely abolished the Pol II Ser2P signal (Appendix Fig S5A and B). Flavopiridol treatment had the same effect, regardless of exchange of all primary antibodies, secondary antibodies, and fluorophores in the immunofluorescence protocol by alternative ingredients (Appendix Fig S5C and D). To control for artefacts only appearing with super‐resolution microscopy, we carried out two‐color STED microscopy for samples labeled with both alternative sets of antibodies and fluorophores (Appendix Fig S6A and B). An analysis based on gates for the morphology types i‐iii reproduced the relationship of cluster morphology to Pol II phosphorylation (Appendix Fig S6C–H) detected in data acquired by STEDD microscopy (Fig 2C–E). These control experiments support the validity of the conclusions drawn from the analysis of the STEDD microscopy data.

The question might arise how the apparent mutual exclusion of Pol II Ser5P and Pol II Ser2P can be reconciled with retention of the Pol II Ser5P mark for all, or at least a good fraction, of transcript elongation. We assessed the relative localization of Pol II Ser5P and Ser2P in the two‐color 3D‐STED super‐resolution microscopy data (Appendix Fig S6A and B). Here, large Pol II Ser5P clusters show almost no overlap with Pol II Ser2P, whereas small Pol II Ser5P clusters do overlap with Pol II Ser2P spots in some cases (Appendix Fig S6I–P). These results indicate that type ii and iii clusters indeed mostly exclude elongating Pol II. The occasional overlap between type i clusters and Pol II Ser2P spots implies that only type i clusters can contain transcribed genes, and is in line with the broad Pol II Ser2P distribution seen for type i clusters in our STEDD data (Fig 2D). This interpretation also aligns well with a reanalysis of ChIP‐seq data, which shows genes with Pol II Ser5P in the promoter region only, but also genes with Pol II Ser5P additionally in the gene body (Appendix Fig S7A and B). Based on these results, it appears that type i clusters represent recruited and elongating Pol II in association with single genes. Taken together, these results indicate that high levels of recruited Pol II are associated with larger clusters, and the presence of transcribed genes at cluster margins is associated with unfolding of clusters (an overview is sketched in Fig 2G).

### Clusters of recruited Pol II are partially destabilized by hexanediol treatment, form stable patterns of non‐growing domains, and exhibit repeated connection and splitting

**Figure 3:**
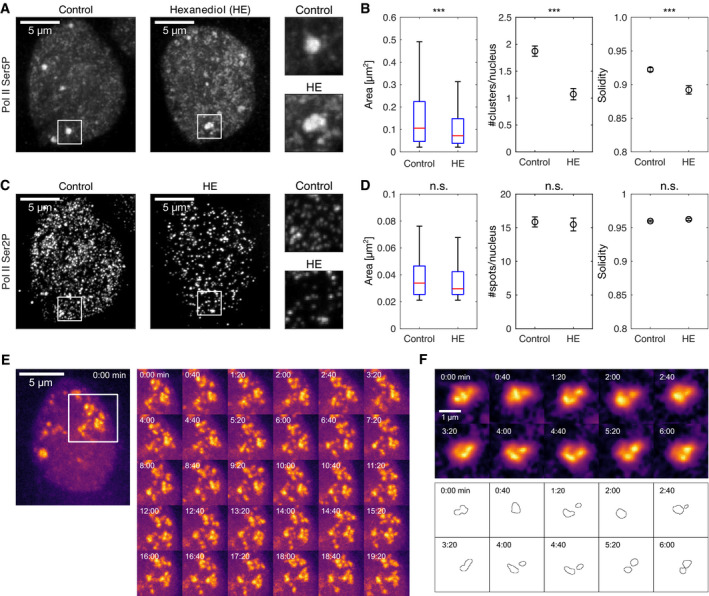
Large clusters of recruited Pol II are partially destabilized by hexanediol treatment, form stable patterns of non‐growing domains, and undergo repeated connection and splitting Representative maximum‐intensity projection micrographs of nuclei in primary cell cultures of zebrafish embryos, treated for 5 min with control media or 3% 1,6‐hexanediol (HE), then fixed and labeled for Pol II Ser5P and Ser2P by immunofluorescence, images recorded by instant‐SIM, this panel shows only the Pol II Ser5P channel.Quantification of changes in Pol II Ser5P cluster morphology upon hexanediol treatment. Area displayed as standard boxplots, solidity and the number of clusters per nucleus are mean±SEM. *** indicates *P* < 0.001 (two‐tailed permutation test for differences upon hexanediol treatment from control, *N* = 5, 3 independent samples; area: *P* < 0.0001 with *n*_Cluster_ = 1,435, 841 clusters for area; number of large clusters (area > 0.2 μm^2^) per nucleus: *P* < 0.0001 with *n*_Nuc_ = 212, 124 nuclei; solidity: *P* < 0.0001 with *n*_Large_ = 401, 135 large clusters).Pol II Ser2P channel micrographs of the same nuclei shown in panel A.Quantification of changes in Pol II Ser2P spots. *n*.s. indicates no statistically significant changes (area: *P* = 0.76 with *n*_Spots_ = 3,394, 1,937 spots; number of clusters per nucleus (no size cut‐off): *P* = 0.80 with *n*_Nuc_ = 212, 124 nuclei; solidity: *P* = 0.21 with *n*_Spots_ = 3,394, 1,937 spots).Representative time‐lapse recording of Pol II Ser5P Fab in a live embryo (no hexanediol treatment). Similar results were observed in two independent experiments, each performed on three different embryos. Images are maximum‐intensity projections, and images recorded by instant‐SIM.Representative close‐up time‐lapse showing transient merging and separation events within a Pol II Ser5P cluster. Single z‐sections, images were bleaching‐corrected and local background was subtracted (radius 3.3 μm). Images from same data set as panel (E). Contour plots are obtained by application of a manually adjusted threshold to assist interpretation, same threshold for all time points.

The relationship between cluster morphologies and Pol II CTD phosphorylation levels raised the question what the underlying mechanisms might be. Previous work has suggested mechanisms related to LLPS (Cho *et al*, 2018; Chong *et al*, 2018; Sabari *et al*, 2018). One common assay to test for LLPS is treatment with 1,6‐hexanediol, which perturbs the molecular interactions implicated in the LLPS of many biological macromolecules (Kroschwald *et al*, 2017). For example, large clusters of Pol II in mESCs were dissolved in response to treatment with 10% hexanediol (Cho *et al*, 2018). More recently, however, hexanediol treatment has been found to interfere with enzymatic activity and genome organization independently of its effect on LLPS. The effects on enzymatic activity gradually increase with the concentration of hexanediol and include the activity of RNA polymerase II (Düster *et al*, 2021). Genome organization is affected on the level of the chromatin fiber and histones, mobility of chromatin, compartment, and contact formation, as well as large‐scale compaction (Itoh *et al*, 2021; Ulianov *et al*, 2021). These effects are pronounced at concentrations of 5%, and less visible at 2.5%. Suggestions for the proper use of hexanediol to assess LLPS include limiting the treatment to 5–10 min (Kroschwald *et al*, 2017). We therefore applied 3% hexanediol for 5 min and, in agreement with previous studies working with similar conditions, found a reduced number of large clusters of recruited Pol II (Sabari *et al*, 2018; Itoh *et al*, 2021) (Fig 3A and B). The remaining large clusters appear more unfolded (Fig 3A), which is reflected in a lowered solidity of large clusters (Fig 3B). The observed changes were specific to clusters of recruited Pol II—spots of elongating Pol II were unaffected by hexanediol treatment in terms of size, number, and solidity (Fig 3C and D). These observations are in line with a model where interactions that can drive phase separation contribute to a certain extent to the formation of large Pol II Ser5P clusters, but Pol II Ser2P spots represent more stably bound Pol II convoys engaged in transcript elongation (Kroschwald *et al*, 2017). A model based solely on LLPS would predict that treatment with hexanediol would largely dissolve Pol II clusters (Kroschwald *et al*, 2017; Li *et al*, 2020), which is an effect we cannot see in our experiments.

Two further hallmark behaviors indicating LLPS are Ostwald ripening and droplet fusion (Brangwynne *et al*, 2009; Bracha *et al*, 2018). Ostwald ripening refers to growth of larger droplets at the expense of shrinking of smaller droplets in their vicinity, mediated by diffusion via the dilute phase and without requirement for contact between droplets. In contrast to this ripening scenario, clusters of recruited Pol II in our experiments persist without appreciable growth or shrinking for well over 10 min (Fig 3E). Droplet fusion occurs between two close‐by droplets that establish contact and merge into a single droplet, which approaches a spherical shape over time. Different from this fusion scenario, we observed that parts of clusters transiently connected and separated again over the course of 1–2 min (Fig 3F). The lack of Ostwald ripening and droplet fusion again indicates that a model based on LLPS alone cannot explain the formation of large clusters of recruited Pol II in our experiments.

### Regulatory chromatin associates with clusters of recruited Pol II, whereas chromatin harboring elongating Pol II is excluded

**Figure 4:**
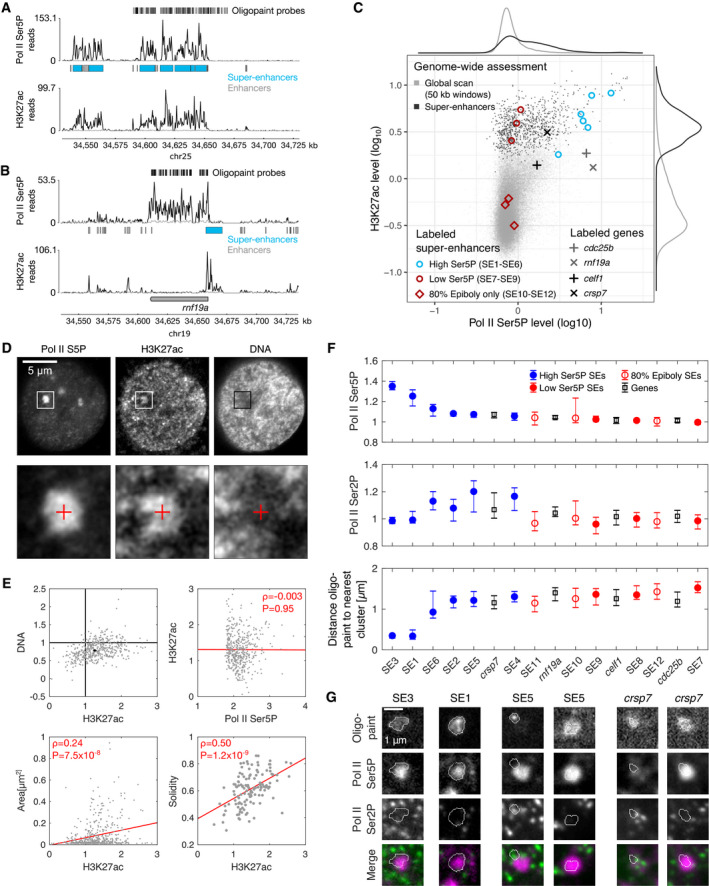
Regulatory chromatin associates with clusters of recruited Pol II Example view of a genomic region containing a super‐enhancer (SE3), for an overview of all genomic regions labeled by oligopaint probe sets see Appendix Fig S8.Example view of a genomic region containing a gene with high Pol II Ser5P levels at the promoter (*rnf19a*).Overview of Pol II Ser5P and H3K27ac ChIP‐seq levels for 50‐kb windows covering the entire genome, windows covering regions containing super‐enhancers, and symbols indicating regions covered by specific oligopaint probe sets.Top row—representative optical section obtained by three‐color STED microscopy from a fixed zebrafish embryo, showing Pol II Ser5P and H3K27ac (immunofluorescence) and DNA (JF646‐Hoechst). Bottom row—Detail views as marked in the top row. The details views are 2.4 μm across.Mean fluorescence intensities (normalized against median intensity of a given nucleus) within Pol II Ser5P clusters. Overall levels of H3K27ac and DNA inside clusters were determined (values for individual clusters in gray, median with 95% bootstrap confidence interval in black) as well as correlations between H3K27ac intensity and Pol II Ser5P intensity, cluster area, and cluster solidity (Pearson correlation coefficient, red line is a linear fit to guide the eye). *n* = 470 clusters and *n* = 130 large clusters (area > 0.08 μm^2^, used for solidity analysis) were extracted from images of *N* = 29 nuclei obtained from five embryos.Assessment of Pol II Ser5P and Pol II Ser2P immunofluorescence signal intensities at the location of super‐enhancer probe sets. Genomic target regions are sorted left‐to‐right by descending Pol II Ser5P signal. Intensities are normalized by the whole nucleus median level, shown is the median with 95% bootstrap confidence intervals. *N* = 2 embryos imaged per probe set, yielding *n* = 67, 66, 91, 63, 53, 70, 93, 63, 71, 78, 59, 58, 103, 55, 65, 127 oligopaint foci for the probe sets SE1, SE2, SE3, SE4, SE5, SE6, SE7, SE8, SE9, SE10, SE11, SE12, *cdc25b*, *celf1*, *crsp7*, *rnf19a*, respectively. Each probe set contained approximately 500 probe sequences, covering 50 kb sequence length, images acquired by instant‐SIM (genomic regions see Appendix Fig S8, probe sets see Materials and Methods).Representative images of indicated oligopaint probe sets and immunofluorescence signal, two examples are shown for SE5 and *crsp7* to illustrate positioning outside a Pol II Ser5P cluster and inclusion into a cluster. Shown are single z‐sections with segmentation outlines of the oligopaint signal.

The formation of macromolecular clusters in transcriptional regulation involves sequence‐specific interactions of proteins with DNA and also specific types of chromatin (Cho *et al*, 2018; Chong *et al*, 2018; Sabari *et al*, 2018; Gibson *et al*, 2019; McSwiggen *et al*, 2019; Shrinivas *et al*, 2019; Li *et al*, 2020; preprint: Trojanowski *et al*, 2021; Zuo *et al*, 2021). Paused Pol II has been specifically implicated in the interaction of enhancers and their target genes (Ghavi‐Helm *et al*, 2014; Bartman *et al*, 2016; Espinola *et al*, 2021; Ing‐Simmons *et al*, 2021). In the case of prominent and long‐lived Pol II clusters, seen in our work and in mESCs previously (Cho *et al*, 2018), a role was suggested for genomic regions that contain many gene regulatory modules, sometimes called super‐enhancers (Sabari *et al*, 2018; Guo *et al*, 2019). To assess the role of super‐enhancer regions in the establishment of Pol II Ser5P clusters, we followed previously published work, using ChIP‐seq data to identify super‐enhancers in the zebrafish embryo (Bogdanović *et al*, 2012; Pérez‐Rico *et al*, 2017). The analysis was based on the histone 3 lysine 27 acetylation (H3K27ac) mark, which distinguishes active from poised regulatory regions (Creyghton *et al*, 2010). Indeed, we found examples of super‐enhancers with elevated levels of Pol II Ser5P (Fig 4A). Super‐enhancers with low levels of Pol II Ser5P as well as genes with elevated Pol II Ser5P levels could also be found (Fig 4B). A comprehensive comparison reveals that super‐enhancers cover a continuum of Pol II Ser5P levels, with some super‐enhancers exhibiting up to 10‐fold higher Pol II Ser5P levels compared to the overall genomic background (Fig 4C). This reanalysis of ChIP‐seq data suggests that, indeed, some super‐enhancers might contribute to structures with high levels of Pol II Ser5P.

To assess how the inferred association between super‐enhancers and structures with high Pol II Ser5P levels translates into three‐dimensional organization, we carried out three‐color STED microscopy of Pol II Ser5P, the H3K27ac mark, and bulk DNA (Fig 4D). In the obtained images, the H3K27ac mark was enriched inside large Pol II Ser5P clusters (Fig 4E). This enrichment was observed despite an overall reduction in bulk DNA content in these clusters, implying a selective retention of chromatin regions with the H3K27ac mark (Fig 4E). A relation between H3K27ac‐enriched chromatin and large Pol II Ser5P clusters was further supported by a positive correlation of H3K27ac levels with the cluster size (Fig 4E). Nevertheless, H3K27ac signal occurred also outside the large Pol II Ser5P clusters, so that only a part of the overall signal was associated with these large clusters (Fig 4D). For large clusters, high H3K27ac signal was also correlated with increased solidity (Fig 4E). These observations are in line with our ChIP‐seq analysis, suggesting that some of the H3K27ac‐marked chromatin regions associate with and potentially contribute to the formation of Pol II Ser5P clusters.

To address the association of specific genomic regions with Pol II Ser5P clusters, we applied DNA fluorescence *in situ* hybridization (FISH) based on the oligopaint approach (Beliveau *et al*, 2012). Specifically, we designed probe sets against genomic regions containing super‐enhancers with high Pol II Ser5P ChIP‐seq signal, super‐enhancers with low Pol II Ser5P signal, and super‐enhancers that are only H3K27ac‐marked at another stage of development, as well as genes with high H3K27ac and Pol II Ser5P signal (Fig 4C and Appendix Fig S8). As expected, super‐enhancers with high Pol II Ser5P ChIP‐seq signal also exhibited high Pol II Ser5P signal at the location of the oligopaint fluorescence label (Fig 4F). All other classes of regions exhibited lower levels of Pol II Ser5P intensity, except for the gene *crsp7* (Fig 4F). High Pol II Ser5P intensities were associated with small distance to the nearest Pol II Ser5P cluster (Fig 4F). These results indicate that super‐enhancers and some genes with high Pol II Ser5P signal in ChIP experiments indeed associate with clusters of recruited Pol II.

Some super‐enhancer regions with high Pol II Ser5P intensities (SE2, SE4, SE5, SE6) exhibited increased Pol II Ser2P intensities (Fig 4F). These super‐enhancer regions were marked by Pol II Ser5P ChIP‐seq signal that was confined to only a small sub‐part of the super‐enhancer region (Appendix Fig S8). These confined ChIP‐seq profiles appeared similar to the profile at the *crsp7* gene and distinct from the more extensive profiles at super‐enhancers with low Pol II Ser2P intensity (SE1, SE3; Appendix Fig S8). The super‐enhancers with these more confined Pol II Ser5P profiles as well as the gene *crsp7* were further away from Pol II Ser5P clusters than those super‐enhancers with low Pol II Ser2P signal (Fig 4F). Visual inspection indicated that all super‐enhancers with high Pol II Ser5P (and the gene *crsp7*) could fully integrate into Pol II Ser5P clusters, whereas those super‐enhancers where the Pol II Ser5P ChIP‐seq profile is sharp (and *crsp7*) could also localize to the cluster surface in conjunction with Pol II Ser2P spots (Fig 4G). These observations imply that super‐enhancers and some genes with high Pol II Ser5P levels can localize to the interior of clusters of recruited Pol II and, in conjunction with elongating Pol II, can alternatively localize to the cluster surface.

### A theoretical model of cluster formation by condensation on surfaces provided by regulatory chromatin regions

**Figure 5:**
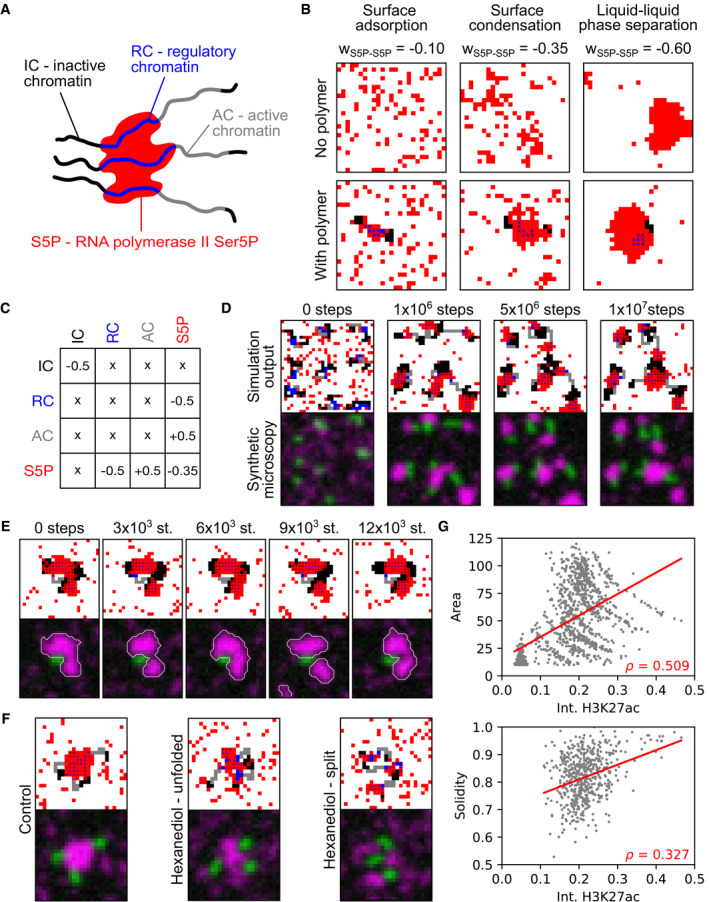
A lattice model exhibits key characteristics of liquid‐phase condensation with a polymeric subregion as a surface Sketch of cluster nucleation with the different species involved in the model.Examples of lattice configurations obtained from simulations containing only red particles with increasing self‐affinity (*w*_S5P‐S5P_, as indicated). Lattice simulations containing a polymer chain of length *L*_polymer_ = 20 with *N*_IC_ = 12 black monomers (black‐black affinity *w*_IC‐IC_ = −0.5) and *N*_RC_ = 8 blue monomers (blue‐red affinity *w*_RC‐S5P_ = −0.5, adjustment see Appendix Fig S10), same *w*_S5P‐S5P_ values as for simulations without polymer chain. All simulations on 25‐by‐25 lattices, *N*_S5P_ = 100 red particles, for adjustment of *N*_S5P_, see Appendix Fig S10A.Interaction matrix for different species in the lattice model. Affinity is represented by negative and repulsion by positive values.Long time behavior (total of 1 × 10^7^ iteration steps) of the model shown as lattice output and synthetic microscopy images, 30‐by‐30 lattice, *N*_S5P_ = 140, nine chains, extent of blue and gray regions chosen randomly for each chain at initialization.Time‐lapse showing transient separation and merging events of a S5P cluster. 25‐by‐25 lattice, *N*_S5P_ = 100, four chains.Simulated hexanediol treatment. 25‐by‐25 lattices, *N*_S5P_ = 100, three chains, *N*_RC_ = 8, *N*_AC_ = 6, modified parameter values in hexanediol simulations: *w*_S5P‐S5P_ = −0.15, *w*_S5P‐RC_ = −0.25.Correlations between H3K27ac intensity (RC) and cluster area (in units of lattice cells, *n* = 1,132) and cluster solidity (only including clusters with area greater 50, *n* = 551, *ρ* is the Pearson correlation coefficient, red line—linear fit to guide the eye). Analysis based on 30 simulations of 25‐by‐25 lattices, *N*_S5P_ = 100, four chains per lattice, extent of blue and gray regions randomly assigned per chain at initialization of each simulation.

Formation of macromolecular clusters at genomic target regions has recently been described using a model of liquid‐phase condensation on microscopic surfaces provided by polymers (Cho *et al*, 2018; Sabari *et al*, 2018; Shin *et al*, 2018; Li *et al*, 2020; preprint: Morin *et al*, 2020; Quail *et al*, 2021; preprint: Renger *et al*, 2021). To test whether such a model can reproduce the different cluster morphologies seen in our experiments, we implemented corresponding lattice kinetic Monte Carlo (LKMC) simulations (Larson *et al*, 1985; Miermans & Broedersz, 2020) (for details, see Materials and Methods and Appendix Fig S9A–F). The LKMC simulation framework has been used previously to simulate the dynamics of chromosomes and macromolecules involved in transcription (Miermans & Broedersz, 2020; Hilbert *et al*, 2021) and provides a coarse‐grained representation of the relevant macromolecules. Specifically, we introduced a “red” particle species that represents the material forming the clusters enriched in recruited Pol II (Fig 5A). In line with previous work (Cho *et al*, 2018; Guo *et al*, 2019), particles of this species exhibit self‐affinity (represented by a negative interaction energy, *w*_S5P‐S5P_ < 0) that can, given sufficiently high affinity and bulk concentration, support formation of a concentrated droplet phase via canonical LLPS (Fig 5B). Previous work, however, suggested that the formation of Pol II‐enriched clusters does not proceed by canonical LLPS, but rather occurs by condensation specifically on regulatory sites within chromatin (Cho *et al*, 2018; Sabari *et al*, 2018; McSwiggen *et al*, 2019; Shrinivas *et al*, 2019). We therefore used polymer chains to represent chromatin (Fig 5A). These polymers contain “blue” subregions that represent regions of regulatory chromatin (RC) and have an affinity to the red particles (*w*_RC‐S5P_ < 0, see Fig 5B). The polymers also contain neutral “black” segments, which have no affinity to red particles (IC, for inactive chromatin). Inactive chromatin segregates from other nuclear components, which was modeled by self‐affinity of these inactive regions (*w*_IC‐IC_ < 0) (Falk *et al*, 2019; Hilbert *et al*, 2021). The addition of a polymer containing a regulatory region can facilitate cluster formation even under conditions where red particles would otherwise not phase‐separate (Fig 5B, for adjustment of the concentration of red particles and *w*_RC‐S5P_, see Appendix Figs S10A and B, and S11). This behavior is typical of liquid‐phase condensation on a surface (Cahn, 1977; Ebner & Saam, 1977; Pandit *et al*, 1982; preprint: Morin *et al*, 2020; Quail *et al*, 2021). To complete our model, we introduced one more type of polymer region that represents chromatin harboring elongating Pol II (colored gray, Fig 5A). Based on the observation that elongating Pol II is excluded from droplets enriched in recruited Pol II (Hu *et al*, 2009; Guo *et al*, 2019; Li *et al*, 2019), we introduce repulsion between gray subregions and red particles (*w*_AC‐S5P_ > 0, Fig 5C). The inactive chromatin regions were placed next to the blue‐gray domain. The combination of affinity parameters formed an affinity matrix, which provides an overview of the coarse‐grained material properties that determine the organization in our theoretical model (Fig 5C).

The assumptions underlying our model were chosen so that several of our experimental observations can be expected to be reproduced. To compare simulation output to microscopy data, we produced synthetic microscopy images. To generate Pol II Ser5P and Pol II Ser2P intensity images, we applied a limited resolution filter and detector noise to the lattice distribution of red and gray particles, respectively. Similar to our time‐lapse microscopy, these synthetic images showed that recruited Pol II forms a long‐term stable pattern of clusters without apparent growth or ripening (Fig 5D). The repeated connection and splitting of parts of clusters seen in time‐lapse microscopy were also visible in simulations (Fig 5E). One key property of surface condensation is that weaker interactions within the liquid phase reduce the size of the condensed phase, while particles will still coat condensation surfaces (preprint: Morin *et al*, 2020; Quail *et al*, 2021). We see that, indeed, reduction of red‐red affinity markedly reduces the area of clusters formed from red particles, while particles still coat the polymer chains (Fig 5F). This effect is in line with the observations upon hexanediol treatment (Fig 3A–D) and underscores the contribution of interactions within the liquid phase to the integrity of large clusters of recruited Pol II. Another key property of surface condensation is that the size of condensates depends on the amount of available surface (preprint: Morin *et al*, 2020). Indeed, our simulations show that cluster area positively correlates with the level of regulatory chromatin (Fig 5G). This correlation is also in line with the correlation between H3K27ac levels and cluster area in our experiments (Fig 4E). Lastly, higher levels of regulatory chromatin in large clusters were positively correlated with cluster solidity (Fig 5G), which also agrees with our experimental observations (Fig 4E). Taken together, simulations of the lattice model reproduce key behaviors expected for a scenario where clusters of recruited Pol II are formed by surface condensation on regulatory chromatin.

Beyond the behaviors that could be directly compared to our experimental observations, our theoretical model can illustrate further differences between a canonical LLPS scenario and surface condensation. To this end, we extracted synthetic microscopy images of the distribution of red particles from simulations without and with a polymer chain that can serve as a condensation surface (Appendix Fig S12). In the absence of a polymer chain, a dilute and a dense phase could be detected over a range of bulk concentrations of the red particles (Appendix Fig S12). Within the bulk concentration range in which two phases coexist, the concentration in both phases was approximately constant, despite changes in bulk concentration (gray‐shaded region in Appendix Fig S12). The bulk concentration range of coexistence was extended when the interaction between red particles was made stronger via adjustment of *w*_S5P‐S5P_ (Appendix Fig S12). These behaviors are strongly indicative of canonical LLPS (Alberti *et al*, 2019). Upon addition of a polymer chain, the high concentration phase could already be detected at lower bulk concentrations (additional blue‐shaded region in Appendix Fig S12). Such formation of clusters at sub‐saturated bulk concentrations in the presence of a polymer surface can be used as a clear indicator of surface‐mediated condensation. In experiments where the polymer surface cannot be removed or deactivated, one can still see that, in this surface condensation regime, the concentration of the dilute phase increases approximately proportionally with the bulk concentration, despite the presence of a second, dense phase (red line within the blue‐shaded regions in Appendix Fig S12). This analysis indicates two behaviors that distinguish the surface condensation regime from the canonical LLPS regime: (i) cluster formation at sub‐saturated bulk concentration and (ii) an increase in dilute phase concentration with bulk phase concentration in the presence of a dense phase.

### Tests of the theoretical model clarify the roles of recruited and elongating Pol II

**Figure 6:**
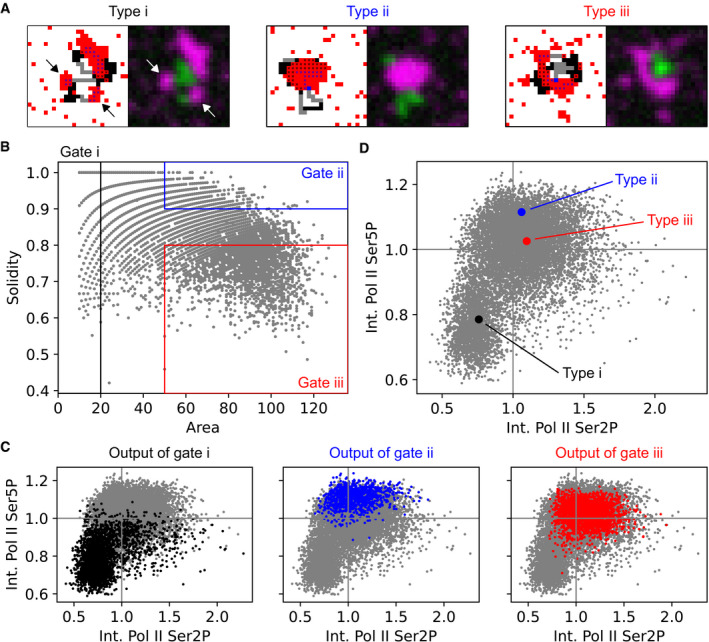
Lattice simulations reproduce the relation between Pol II CTD phosphorylation and cluster morphology Example lattice configurations for all three cluster morphology cluster types (i–iii) are shown as lattice simulation output and the corresponding synthetic microscopy images.Area and solidity of individual clusters, with gate regions for the typical morphologies i–iii. Clusters were segmented based on Pol II Ser5P intensity (total 11,248 clusters). For each simulation, four chains with regions of randomly assigned length of *N*_RC_∈{0,2,4,6,8} blue monomers and *N*_AC_∈{0,3,6} gray monomers were placed, total chain length *L*_polymer_ = 20. 2,808 clusters in gate i, 1,021 clusters in gate ii, and 2,469 clusters in gate iii.The Pol II Ser5P and Pol II Ser2P intensities (mean intensity across all pixels inside a given cluster's segmentation mask) of the clusters in gates i–iii (color), ungated cluster population in gray. Intensities were scaled by the population median.Pol II Ser5P and Pol II Ser2P levels of the gated clusters in panel (C) plotted over the ungated population of clusters, median with 95% bootstrap confidence interval. For all types (i–iii), the confidence interval is hidden by the median data point.

To proceed from construction to testing of our theoretical model, we assessed the relation between cluster morphologies and levels of recruited and elongating Pol II. To this end, we simulated clusters formed from polymer chains with blue and gray regions of randomly varied extent. These simulations indeed produced configurations that resemble the cluster types i‐iii seen in our microscopy images (Fig 6A). The synthetic microscopy images allowed us to apply the same morphology‐based gating into type i‐iii clusters as in the real microscopy data (Fig 6B). By this analysis, we found a similar relationship of Pol II Ser5P and Pol II Ser2P levels to cluster type as for our experimental data (Fig 6C and D). This agreement supports our model, in the sense that recruited Pol II drives cluster formation by condensation on regulatory chromatin, whereas chromatin harboring elongating Pol II drives unfolding of clusters.

Our theoretical model includes interactions of red particles with regulatory chromatin as well as particle–particle interactions. Several reports on the role of transient particle–chromatin binding (Cho *et al*, 2018; Chong *et al*, 2018; Sabari *et al*, 2018; Gibson *et al*, 2019; McSwiggen *et al*, 2019; Shrinivas *et al*, 2019; Li *et al*, 2020; preprint: Trojanowski *et al*, 2021; Zuo *et al*, 2021) raise the question of whether a “binding‐only” model is sufficient to explain cluster formation. To implement such a binding‐only scenario, we removed interactions between red particles (*w*_S5P‐S5P_ = 0) and restrained particle–chromatin interactions to be strictly local. Without further change to any of the other interactions, the size of the clusters is markedly reduced relative to the full model (Appendix Fig S13A). A 10‐fold increase in particle affinity (*w*_RC‐S5P_ = −5.0) for regulatory chromatin can compensate for the reduction in cluster size, but the relationship between cluster size (area) and morphology (solidity) is changed relative to the full model (Appendix Fig S13B). In particular, large clusters in the binding‐only model exhibit markedly reduced solidity, both for unmodified and 10‐fold increased particle–chromatin affinity (Appendix Fig S13C). A binding‐only model can thus explain cluster formation in general, but does not reproduce the large and compact clusters (type ii morphology) seen in our data (Fig 2B–E). Our full theoretical model, which includes interactions between the particles that bind to regulatory chromatin, does produce such large clusters with compact morphologies. Accordingly, our investigation implies that clusters form by association of recruited Pol II with regulatory chromatin, and are enlarged and compacted by interactions amongst the particles that form these clusters.

### Effects of transcription inhibitors on cluster shape are reproduced by a model based on surface condensation and unfolding by exclusion

**Figure 7:**
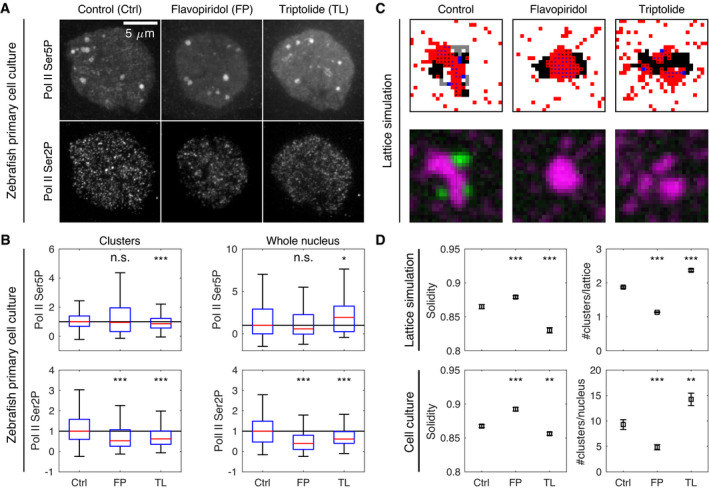
Lattice simulations reproduce the effect of transcription inhibitors on cluster morphology Representative micrographs showing Pol II Ser5P and Pol II Ser2P in primary cell cultures obtained from zebrafish embryos and treated for 30 min with control media (Ctrl), flavopiridol (FP, 1 μM), or triptolide (TL, 500 nM). Cell cultures were fixed at the end of treatment, labeled by immunofluorescence, and microscopy images were acquired by instant‐SIM. Shown are maximum‐intensity intensity projections, same color scaling used across conditions.Pol II Ser5P and Pol II Ser2P intensities at Pol II Ser5P clusters and throughout entire nuclei, standard boxplots. *** indicates *P* < 0.0003, * indicates *P* < 0.017, n.s. indicates *P* ≥ 0.017, significance levels Bonferroni‐corrected for multiple testing (two‐tailed permutation test for differences from control, data obtained from three independent sets of experiments; Pol II Ser5P at clusters: *P* = 0.08, *P* < 0.0001, *n* = 1,534, 716, 1,682 clusters; Pol II Ser2P at clusters: *P* < 0.0001, *P* < 0.0001, *n* = 1,534, 716, 1,682 clusters; Pol II Ser5P throughout nuclei: *P* = 0.17, *P* = 0.014 with *n* = 165, 148, 118 nuclei; Pol II Ser2P throughout nuclei: *P* < 0.0001, *P* = 0.0001 with *n* = 165, 148, 118 nuclei; data obtained from three independent experiments). For additional properties and actinomycin D treatment, see Appendix Fig S14A.Examples of lattice configurations obtained from simulations with modifications that mimic inhibitor treatments. For flavopiridol treatment, no gray regions were assigned. For triptolide, no gray regions were assigned, the Pol Ser5P self‐affinity (*w*_S5P‐S5P_ = −0.25) and affinity to regulatory regions (*w*_RC‐RC_ = −0.25) were reduced, and the number of red particles was increased from 100 to 120.Cluster solidity and the number of clusters obtained from lattice simulations and microscopy images of cell cultures, mean±SEM. Quantification was based on synthetic microscopy images, *n* = 1,000 images analyzed per condition. For lattice simulations, *** indicates *P* < 0.0005; for cell cultures, *** indicates *P* < 0.0003, ** indicates *P* < 0.003 (simulations, solidity: *P* < 0.0001, *P* < 0.0001 with *n* = 1,000, 1,000, 1,000; simulations, number of clusters: *P* < 0.0001, *P* < 0.0001 *n* = 1,000, 1,000, 1,000; cell culture, solidity: *P* < 0.0001, *P* = 0.0007 with *n* = 1,514, 703, 1,631 clusters; cell culture, number of clusters: *P* < 0.0001, *P* = 0.001 with *n* = 165, 148, 118 nuclei).

To further test the interplay between surface condensation and cluster unfolding described by the theoretical model, we applied flavopiridol and triptolide, two transcription inhibitors with distinct effects on Pol II initiation and pause release (Fig 7A, comprehensive assessment in Appendix Fig S14A, additional example images Appendix Fig S15) (Bensaude, 2011; Jonkers *et al*, 2014). In line with its known effect, the CDK9 inhibitor flavopiridol reduced Pol II Ser2P levels at clusters as well as throughout the nucleus within 30 min of treatment (Fig 7B) (Bensaude, 2011). Triptolide is an inhibitor of the ATPase activity of XPB, the helicase/translocase subunit of TFIIH, and has multiple effects on Pol II (Titov *et al*, 2011; Wang *et al*, 2011; Manzo *et al*, 2012; Jonkers *et al*, 2014; Steurer *et al*, 2018). Triptolide treatment is known to result in an overall degradation of Pol II, which also occurred within the 30‐min triptolide treatment in our experiments (Appendix Fig S16A and B). This degradation proceeds via Pol II Ser5 hyperphosporylation, which was seen in our experiments as an increase of Pol II Ser5P levels throughout the nucleus (Fig 7B). The increased Pol II Ser5P levels, however, do not lead to increased levels of transcription elongation. Instead, the inhibition of the helicase/translocase activity of TFIIH hinders the progression of Pol II into elongation, seen in our experiments as a reduction of Pol II Ser2P at clusters as well as throughout the nucleus (Fig 7B). As the progression toward elongation is hindered, Pol II Ser5P is released from chromatin and becomes mobile to explore the nuclear space, seen in our experiments as decreased Pol II Ser5P levels at clusters (Fig 7B). This assessment indicates that flavopiridol and triptolide primarily affect Pol II pause release and initiation, respectively.

We implemented the effect of both inhibitors in the lattice simulations to assess the resulting changes in cluster morphology. To mimic the effect of flavopiridol, we replaced all gray polymer regions (elongated gene bodies) with black polymer regions (inactive chromatin) (Fig 7C). The modified simulations showed fewer and rounder clusters of Pol II Ser5P, as was also seen in the experimental data for this inhibitor (Fig 7D). Our model thus accurately reproduced the effect of flavopiridol on cluster shape and pinpoints the contribution of transcribed gene bodies to the unfolding of clusters of recruited Pol II.

To mimic the effect of triptolide, we also removed gray regions, additionally reduced the affinity amongst red particles (*w*_S5P‐S5P_) and between red and blue particles (*w*_S5P‐RC_) to mimic the loss of Pol II Ser5P from chromatin, and increased the number of red particles (representing Pol II SerP) to mimic the hyperphosphorylation seen in our experiments (Fig 7C). The modified simulations showed a higher number of clusters, which were more unfolded, as was also seen in the experimental data for this inhibitor (Fig 7D). Our model thus also reproduced the effect of triptolide on cluster shape and underlines the role of Pol II Ser5P affinity in maintaining the integrity of clusters of recruited Pol II.

Considering that the model is based on affinity differences related to Pol II CTD phosphorylation, it is expected that treatments that act not primarily through changes in this phosphorylation should not be captured by the model. To test this limitation, we applied actinomycin D, which inhibits transcription by intercalating with DNA (Bensaude, 2011) (Appendix Fig S15). The phosphorylation changes associated with actinomycin D treatment were similar to the effect of flavopiridol: Pol II Ser5P at clusters was not changed, whereas Pol II Ser2P levels were decreased (Appendix Fig S14A). The changes in cluster shape upon actinomycin D treatment, however, were different from the model results for flavopiridol: Solidity decreased and the number of clusters increased, representative of cluster unfolding and splitting, resembling triptolide treatment (Appendix Fig S14A). Accordingly, our theoretical model cannot coherently explain the effects of actinomycin D, suggesting that the model's explanatory power is limited to inhibitors that perturb transcriptional regulation primarily via Pol II CTD phosphorylation.

We propose that cluster morphology is shaped by the interplay of surface condensation and cluster unfolding. These processes should, in essence, apply independently of specific circumstances, for example, the specific cell type. We thus repeated the inhibitor experiments in a human cell line (THP‐1, undifferentiated, Appendix Fig S15). Whereas clusters were smaller and fewer in number, the changes in phosphorylation levels and morphology of clusters upon triptolide and flavopiridol treatment directly corresponded to those in zebrafish cells (Appendix Fig S14B). Again, the application of actinomycin D resulted in a morphological change (increased solidity) that could not be explained by changes in phosphorylation levels (no change, Appendix Fig S14B). These results demonstrate that our conclusions can be transferred to at least one other cell type.

## Discussion

In this study, we investigated how recruited and elongating Pol II contribute to the morphology of macromolecular clusters enriched in Pol II. Our findings indicate that formation of these clusters can be understood as the condensation of a liquid on surfaces provided by regulatory chromatin regions. Based on recent *in vitro* experiments, surface condensation can serve as a model for the formation of a liquid film on microscopic condensation surfaces provided by DNA (preprint: Morin *et al*, 2020; Quail *et al*, 2021; preprint: Renger *et al*, 2021). Surface condensation has been extensively characterized outside of biology as a process in which affinity for a surface allows formation of growth‐limited condensates from a sub‐saturated liquid phase (Cahn, 1977; Ebner & Saam, 1977; Pandit *et al*, 1982). Surface condensation can be distinguished from canonical LLPS. Canonical LLPS occurs when a liquid phase exceeds the saturation concentration, so that droplets can form spontaneously or by nucleation, stabilize upon exceeding a critical radius, and undergo fusion and coarsening toward increasing sizes (Bracha *et al*, 2018; Narayanan *et al*, 2019). Nucleation and coarsening dynamics in canonical LLPS can be influenced by the involvement of genomic regions and transcriptional activity (Berry *et al*, 2015; Shin *et al*, 2018). In these cases, droplets nevertheless exhibited several hallmarks of LLPS: smooth surfaces, fusion, and ripening. In other cases of formation of macromolecular clusters with the involvement of specific genomic regions, however, the absence of smooth droplet surfaces as well as a limitation of the growth of clusters indicate processes that differ from canonical LLPS (Cho *et al*, 2016, 2018; Sabari *et al*, 2018; Shrinivas *et al*, 2019; Li *et al*, 2020; Rippe, 2021). Our explanation of Pol II clustering in zebrafish embryos by surface condensation on regulatory chromatin might illustrate an explanation for cluster formation also in these and related biological systems.

A second conclusion suggested by our study is that chromatin regions containing elongating Pol II are excluded from the Pol II Ser5P‐enriched condensate, resulting in an unfolded morphology of clusters with high levels of elongation. This exclusion might be an explanation for previous observations of unfolded Pol II clusters with transcribing Pol II at their surfaces (Eskiw *et al*, 2008). Our conclusions correspond well with another recently proposed model, where RNA produced at gene regulatory elements supports the formation of condensates, whereas RNA produced during elongation of gene bodies can drive their dissolution (Nozawa *et al*, 2017; Yin *et al*, 2020; Henninger *et al*, 2021; preprint: Shao *et al*, 2021). Such an RNA‐mediated role of elongation in unfolding of macromolecular assemblies is also in line with previous work implicating RNA in the unfolding of chromatin regions harboring transcribed genes (Nozawa *et al*, 2017; Michieletto & Gilbert, 2019; Yin *et al*, 2020; Hilbert *et al*, 2021; Lu *et al*, 2021). Complex morphologies of phase‐separated droplets can also occur in multi‐component condensates (Feric *et al*, 2016). Such a multi‐component perspective might be applicable to Pol II clusters, considering that the phosphorylation states of recruited and elongating Pol II result in mutual spatial exclusion (Hu *et al*, 2009; Lu *et al*, 2018; Guo *et al*, 2019; Li *et al*, 2019). Recruitment and elongation would thus occur in close‐by, though spatially segregated, compartments, which are tied to each other through common chromatin templates. This idea has been proposed for Mediator‐Pol II clusters in mESCs (Cho *et al*, 2018), and our work might provide a theoretical model for such a compartmentalization. The idea also fits with the recent observation of chromatin domains that move as connected units (Zidovska *et al*, 2013; Shaban *et al*, 2018; Ashwin *et al*, 2019).

In our time‐lapse recordings during interphase, Pol II clusters maintained different types of morphologies over 10 min and longer. Similarly, it was previously observed that nuclear bodies and their three‐dimensional organization remain stable over long times once they are established after cell division (Tsukamoto *et al*, 2000). Also, transcription largely shuts down during mitosis in zebrafish blastula cells during cell division (Hilbert *et al*, 2021). It would therefore be interesting to assess how Pol II clusters are reestablished following cell division. The reshaping and re‐establishment of Pol II foci have recently also been connected to ATP‐dependent catalytic processes associated with nuclear actin and myosin (preprint: Baarlink *et al*, 2017; preprint: Hari‐Gupta *et al*, 2020; Wei *et al*, 2020b). A fine‐grained analysis of fluctuations of cluster morphologies might allow an assessment of how far active catalytic processes result in characteristic non‐equilibrium fluctuation signatures (Battle *et al*, 2016). Taken together, there is indication of a number of catalytic and mechanochemical processes that might contribute to the establishment and changes of Pol II cluster morphology and await further investigation.

We categorized cluster morphologies based on size (area) and one characteristic that captures the apparent degree of unfolding (solidity). This characterization was sufficient to establish a direct relationship between Pol II phosphorylation and cluster morphology. These findings warrant a more comprehensive morphological characterization. Especially those clusters with complex morphologies—resembling, for example, donuts, croissants, or jalebis—provide an intriguing variety of shapes to support further studies. Amongst these, the donut‐shaped clusters that persist for 10 min and longer are most intriguing, and might be connected to the looping of transcription termination sites to the promoter region (O'Sullivan *et al*, 2004; Tan‐Wong *et al*, 2008) or the overall gene body (Bonev *et al*, 2017). Considering that large and persistent Pol II clusters were initially observed in pluripotent cells (Cho *et al*, 2016), they might be specific to developmental gene regulation. This view is supported by the implication of paused and elongating Pol II in the clustering of genes and enhancers in early development of *Drosophila* and mice (Ghavi‐Helm *et al*, 2014; Bonev *et al*, 2017; Espinola *et al*, 2021; Ing‐Simmons *et al*, 2021).

Our model of cluster formation is coarse‐grained in nature, compressing the molecular reality of the biological cell into a small number of generalized components and affinities. For example, the control of Pol II initiation, pausing, and pause release proceeds along numerous steps, and the list of the involved regulatory factors is continuously expanding (Kimura *et al*, 2002; Darzacq *et al*, 2007; Steurer *et al*, 2018; Li *et al*, 2019). Two key regulators that were identified in zebrafish embryos are p300 and BRD4, which are associated with the H3K27ac active chromatin mark (Chan *et al*, 2019; Sato *et al*, 2019). H3K27ac has also been identified as a crucial requirement for pause release into elongation (Stasevich *et al*, 2014) as well as a direct modifier of chromatin phase separation behavior (Gibson *et al*, 2019). Additional assessment of these regulators with respect to the morphology of Pol II‐enriched clusters should thus provide a more comprehensive understanding.

Finally, the question remains as to how far the observed clusters and their spatial organization relate to the control of transcription. One key issue is the control of genes by enhancers over sequence distances of tens or even hundreds of kilobases. Proximity of an enhancer to a target gene in three‐dimensional space can, for example, trigger Pol II pause release (Bartman *et al*, 2016). Transient proximal placement of an enhancer within a distance of a few 100 nm is sufficient to induce transcription, whereas persistent association and direct molecular binding seem not to be required (Chen *et al*, 2018; Li *et al*, 2020). In line with these observations, the clusters of recruited Pol II in our work exhibit diameters of a few 100 nm, contain genomic regions that harbor enhancers, and are only occasionally visited by genes that undergo transcription. Together with *in vitro* observations of the zipping together of DNA strands by a liquid phase (Quail *et al*, 2021; Zuo *et al*, 2021), our work implies that this loose engagement of genes with enhancers might be facilitated by liquid bridges. During the time of engagement, such bridges would permit the transfer of transcription factors that are collected via the enhancer to the regulated gene, where they can be exploited for transcriptional activation even after the liquid bridge is broken (Cho *et al*, 2018; Brandão *et al*, 2021; preprint: Shao *et al*, 2021; preprint: Trojanowski *et al*, 2021; Xiao *et al*, 2021; preprint: Zuin *et al*, 2021). Liquid bridges between different regions of the genome could also serve as mechanical connections, contributing to the 3D organization of chromatin within and in the neighborhood of Pol II clusters (Nozaki *et al*, 2017; Nagashima *et al*, 2019).

## Materials and Methods

### Zebrafish husbandry

Fish were raised and bred according to local regulations in the fish facility of the Institute of Biological and Chemical Systems. Embryos were obtained by spontaneous mating. Embryos were dechorionated with Pronase, washed with E3 embryo medium, and subsequently kept in agarose‐coated dishes in 0.3× Danieau's solution at 28.5°C.

### Imaging of Pol II phosphorylation states in live zebrafish embryos

Covalently labelled antigen binding fragments (Fab) were injected into the yolk of dechorionated embryos at the single cell stage. Per embryo, 1 nl of Fab mix (0.2 μl 1% Phenol Red, 1.5 μl A488‐labeled anti‐Pol II Ser2P Fab, 2.3 μl Cy3‐labeled anti‐Pol II Ser5P Fab, Fab stock concentration ≈ 1 mg/ml) was injected. Embryos were mounted at the high stage in 0.7% low melting point agarose in 0.3× Danieau's solution in ibidi 35 mm imaging dishes (#1.5 selected glass cover slips). Embryos for additional fixed imaging were taken from those injected for live imaging, transferred to a fixation solution at the sphere stage (2% formaldehyde, 0.2% Tween‐20 in 0.3x Danieau's embryo media), left to fix at 4°C overnight, washed three times with Dulbecco's formulation PBS with 0.1% Tween‐20 (PBST), and mounted for imaging in VectaShield H‐1000 using #1.5 selected glass cover slips.

### Primary cell culture from zebrafish embryos

Fish embryos were collected in the oblong stage and moved to low‐retention microcentrifuge tubes. The embryos were deyolked through vortexing in deyolking buffer (55 mM NaCl, 1.75 mMKCl, 1.25 mM NaHCO_3_). Afterward, 1 ml PBS (Dulbecco's formulation) with 0.8 mM CaCl_2_ was added to the samples and incubated for 30 min. Inhibitors were introduced to PBS before distribution to individual culturing tubes. Samples were fixed by addition of 330 μl of 8% Formaldehyde in PBS with 0.8 mM CaCl_2_ to each tube. Tubes were immediately spun down at 800 *g* and left for 15 min at room temperature, and then, the liquid was replaced by 8% formaldehyde in PBS + CaCl_2_, left at room temperature for further fixation for at least 20 min.

### THP‐1 cell culture

Undifferentiated cells from the human monocytic cell line THP‐1 were generously provided by the Weiss laboratory, Institute of Biological and Chemical Systems, Karlsruhe Institute of Technology (Fritsch‐Decker *et al*, 2018). Cells were transferred into low‐retention microcentrifuge tubes directly before experimental treatment, inhibitors were applied by spike‐in and incubated for 30 min at room temperature, and fixation was carried out identically to primary zebrafish cell cultures.

### Inhibitor treatment

All inhibitors were resuspended in DMSO to recommended effective concentrations (Bensaude, 2011). Flavopiridol hydrochloride hydrate (F3055, Sigma‐Aldrich) was resuspended to a stock concentration of 12.5 mM and diluted 1:12,500 to an effective concentration of 1 μM. Actinomycin D (A1510, Sigma‐Aldrich) was resuspended to an initial concentration of 1 mg/ml and diluted 1:200 to an effective concentration of 5 μg/ml. Triptolide (T3652, Sigma‐Aldrich) was resuspended to a stock concentration of 10 mM and diluted 1:20,000 to an effective concentration of 500 nM. The effectiveness of all inhibitors was verified on the basis of Pol II phosphorylation changes at the whole nucleus level (Appendix Fig S14). Alpha‐amanitin (A2263, Sigma‐Aldrich) was micro‐injected into the yolk (1 nl per embryo) at a concentration of 0.2 mg/ml (dissolved in water) at the 1‐cell stage (Joseph *et al*, 2017; Hilbert *et al*, 2021). Whole embryo flavopiridol treatment was carried out by adding 10 μM flavopiridol to the embryo media (Vopalensky *et al*, 2018).

### Hexanediol treatment

3% w/v in PBS + 0.8 mM CaCl_2_ for the last 5 min of 30‐min primary cell culture, then fixation and staining with regular immunofluorescence for instant‐SIM.

### Whole embryo immunofluorescence

Whole embryo samples were obtained by fixing sphere‐stage embryos over the following night at 4°C (2% formaldehyde, 0.2% Tween‐20 in 0.3× Danieau's embryo media). Animal cap parts of these samples were permeabilized in 0.5% Triton X‐100 in PBS for 15 min at room temperature, washed three times with PBST for 10 min, and blocked in 4% BSA in PBST for at least 30 min at room temperature. Primary antibodies were applied over the following night at 4°C in 4% BSA in PBST. Secondary antibodies were applied over the following night at 4°C in 4% BSA in PBST. Primary and secondary antibodies were removed by washing three times with PBST for 5 min. After washing out the secondary antibodies, the samples were again fixed with 4% formaldehyde for 15 min for long‐term retention of antibody staining. In most cases, these post‐fixed embryo samples were free of yolk, and any remaining pieces of yolk were manually removed with fine forceps while transferring samples through three washes of PBST in glass dishes. The deyolked animal caps were mounted using selected #1.5 cover slips. The antibodies, mounting media, and DNA stains used in the different experiments are listed below.

### Cell culture immunofluorescence

Fixed cell cultures were processed for the entire immunofluorescence procedure in the low‐retention microcentrifuge tubes in which they were cultured. Cells were permeabilized with 0.5% Triton X‐100 in PBS for 10 min, washed three times with PBST, and blocked with 1 ml of 4% BSA in PBST for 30 min. Primary antibodies were applied over the following night at 4°C in 4% BSA in PBST. Secondary antibodies were applied over the following night in 4% BSA in PBST. Primary and secondary antibodies were removed by washing three times with PBST. After washing out the secondary antibodies, the samples were again fixed with 8% formaldehyde in PBS for 15 min for long‐term retention of antibody staining. Samples were washed another three times with PBST and then mounted using 30 μl of VectaShield H‐1000 supplemented with a 1:2,500 dilution of Hoechst 33342 (stock concentration 20 mM) using selected #1.5 cover slips. The antibodies used in the different experiments are listed below.

### STEDD sample preparation

Samples for STEDD imaging (Fig 2) were prepared from whole embryos. Primary antibodies (see Table 1): mouse anti‐Pol II Ser5P (4H8, 1:300), rabbit anti‐Pol II Ser2P (EPR18855, 1:2,500). Secondary antibodies (see Table 2): goat anti‐mouse conjugated with STAR RED (1:1,000), donkey anti‐rabbit conjugated with Alexa 488 (1:2,000). Samples were mounted in TDE‐O (Abberior).

**Table 1:** List of primary antibodies. All primary antibodies used for immunofluorescence in this studies were monoclonal and are commercially available.

Target	Type	Clone	Supplier	Cat. No.	Lot No.
Pol II S5P	Mouse IgG	4H8	Abcam	ab5408	GR205997‐15, GR3325973‐3
Pol II S5P	Rat IgG	3E8	Active motif	61986	10618002
Pol II S2P	Mouse IgM	H5	Biolegend	920204	B223109
Pol II S2P	Rabbit IgG	EPR18855	Abcam	ab193468	GR240664‐4, GR240664‐6
Pol II pan	Mouse IgG	8WG16	Invitrogen	MA1‐26249	VJ3115733
H3S28P	Rat IgG	HTA28	Abcam	ab10543	GR3219690‐4
H3K27ac	Rabbit IgG	EP16602	Abcam	ab177178	GR320298

**Table 2:** List of secondary antibodies. All secondary antibodies used for immunofluorescence in this studies were polyclonal and are commercially available.

Antibody	Species	Fluorophore	Supplier	Cat. No.
Anti‐mouse IgG	Goat	Alexa 488	Invitrogen	A11001
Anti‐mouse IgG	Goat	Alexa 594	Invitrogen	A11005
Anti‐mouse IgG	Goat	STAR ORANGE	Abberior	STORANGE‐1001‐500UG
Anti‐mouse IgG	Goat	STAR RED	Abberior	2‐0002‐011‐2
Anti‐mouse IgM	Goat	Alexa 594	Invitrogen	A21044
Anti‐rabbit IgG	Donkey	Alexa 488	Invitrogen	A21206
Anti‐rabbit IgG	Goat	Alexa 594	Invitrogen	A11037
Anti‐rabbit IgG	Goat	STAR 520 SXP	Abberior	ST520SXP‐1002‐500UG
Anti‐rabbit IgG	Goat	STAR ORANGE	Abberior	STORANGE‐1002‐500UG
Anti‐rat IgG	Goat	Alexa 594	Invitrogen	A11007
Anti‐rat IgG	Goat	Alexa 647	Invitrogen	A21247

### Two‐color STED sample preparation

Samples for two‐color STED imaging (Appendix Fig S6) were prepared from whole embryos. Two sets of primary and secondary antibodies were used (see Table 1 and Table 2). The first set of antibodies (AB set 1) contained as primary antibodies: mouse IgG anti‐Pol II Ser5P (4H8, 1:300), rabbit IgG anti‐Pol II Ser2P (EPR18855, 1:300). Secondary antibodies: goat anti‐mouse conjugated with STAR RED (1:300), goat anti‐rabbit conjugated with STAR ORANGE (1:300). The second set of antibodies (AB set 2) contained as primary antibodies: rat IgG anti‐Pol II Ser5P (3E8, 1:300), mouse IgM anti‐Pol II Ser2P (H5, 1:300). Secondary antibodies: goat anti‐rat conjugated with Alexa 647 (1:300), goat anti‐mouse IgG conjugated with Alexa 594 (1:300). Samples were mounted in TDE‐O (Abberior).

### Sample preparation from hexanediol and inhibitor treated cell cultures

Samples were obtained from cell cultures treated with hexanediol (Fig 3) or different transcription inhibitors (Appendix Figs S10, S14 and S15). Primary antibodies (see Table 1): mouse anti‐Pol II Ser5P (4H8, 1:1,000), rabbit anti‐Pol II Ser2P (EPR18855, 1:1,000), rat anti‐H3S28P (HTA28, 1:10,000). Secondary antibodies (see Table 2): goat anti‐mouse conjugated with Alexa 488 (1:1,000), goat anti‐rabbit conjugated with Alexa 594 (1:1,000), goat anti‐rat conjugated with Alexa 647 (1:1,000).

### Three‐color STED sample preparation

Samples for three‐color STED imaging (Fig 3D and E) were prepared from whole embryos. Primary antibodies (see Table 1): mouse anti‐Pol II Ser5P (4H8, 1:300), rabbit anti‐H3K27ac (EP16602, 1:300). Secondary antibodies (see Table 2): goat anti‐mouse conjugated with Alexa 594 (1:300), goat anti‐rabbit conjugated with STAR 520 SXP (1:300). Samples were mounted in glycerol with 10 μM JF646‐Hoechst (gift from the Lavis Lab) (Legant *et al*, 2016; Spahn *et al*, 2019; Zhang *et al*, 2019).

### Oligopaint FISH sample preparation

Samples for combined oligopaint DNA FISH and immunofluorescence (Fig 4F and G) were prepared from whole embryos. Primary antibodies (see Table 1): mouse anti‐Pol II Ser5P (4H8, 1:300), rabbit anti‐Pol II Ser2P (EPR18855, 1:300). Secondary antibodies (see Table 2): goat anti‐mouse conjugated with STAR RED (1:300), goat anti‐rabbit conjugated with Alexa 488 (1:300).

### Sample preparation for not phospho‐specific visualization of Pol II

Samples for the assessment of general Pol II levels after triptolide and alpha‐amanitin treatment (Appendix Fig S16A and B) were prepared from primary zebrafish cell cultures. Primary antibodies (see Table 1): mouse anti‐Pol II (pan CTD, 8WG16, 1:1,000). Secondary antibodies (see Table 2): goat anti‐mouse conjugated with Alexa 594 (1:1,000).

### ChIP‐seq analysis

Raw ChIP‐seq reads were obtained for Pol II Ser5P at dome (GSE4426 (Zhang *et al*, 2014)), H3K27ac at dome (GSE32483 (Bogdanović *et al*, 2012)), input at dome (GSE84602 (Meier *et al*, 2018)), H3K27ac at 80% epiboly (GSE32483 (Bogdanović *et al*, 2012)), and input at 80% epiboly (GSE41458 (Winata *et al*, 2013)). Raw reads were aligned to the zebrafish genome (danRer10) using Bowtie 2 (Langmead *et al*, 2009), filtering out reads with more than 1 mismatch using samtools (Li *et al*, 2009). For coverage tracks, bigWig files were generated and tracks were produced using pyGenomeTracks (Lopez‐Delisle *et al*, 2021). Pol II Ser5P and H3K27ac peaks were called using *macs2 callpeak* from MACS2 (Zhang *et al*, 2008), with Input as control and reads extended to the predicted fragment length from *macs2 predictd*. Identification of super‐enhancers followed previous work in zebrafish (Pérez‐Rico *et al*, 2017), using the program ROSE (Lovén *et al*, 2013; Whyte *et al*, 2013) on the H3K27ac peaks. This removes peaks within 2.5 kb of a transcription start site, stitches together the remaining peaks if they are closer than 12.5 kbs, and identifies super‐enhancers from the resulting list after ranking them by their H3K27ac signal. All underlying pipelines and scripts are provided as a Zenodo repository, see Data availability.

### Oligopaint DNA FISH

Genome homology region oligos for the entire zebrafish genome were used as provided by OligoMiner (Beliveau *et al*, 2018). Six loci with a strong Pol II Ser5P signal in the super‐enhancer window were selected from the list of identified super‐enhancers at the dome stage (SE1–SE6). Two sets of controls were selected: three super‐enhancer loci without a strong Pol II Ser5P signal (SE7–SE9), and three super‐enhancer loci from 80% epiboly, which are not identified as super‐enhancers at the dome stage (SE10–SE12). Additionally, four gene loci with strong H3K27ac signal and strong Ser5P signal in the promoter (*crsp7*, *celf1*) or gene body (*cdc25b*, *rnf19a*) were selected. Around each of these 16 loci, regions ranging between 25 and 100 kb were chosen, so as to obtain at least 300 homology oligos per region. In total, for the 16 regions, 5,989 oligopaint homology oligos were obtained. Primers were designed with the OligoLego tool (Nir *et al*, 2018), and joined to the homology oligos and streets (to which primers bind) to construct oligopaint probes that formed the oligopaint library. The primers were first validated to be potential PCR primers, filtered out to ensure that they do not adopt secondary structures, screened in pairs to avoid cross‐talk when hybridizing, and finally aligned against the zebrafish genome to ensure that none of them align with the genome. A penalty matrix was then built to determine compatibility of possible street pairs. The final oligopaint library was then compiled by appending the streets and the hybridizing oligos in the required configuration, consisting of a universal mainstreet (to which the universal forward primer binds), a locus‐specific mainstreet (to which a locus‐specific forward primer binds), a different universal mainstreet (to which a fluorescent forward primer binds), the homology region oligo (that hybridizes to the target loci), and a universal backstreet (to which the universal reverse primer binds). The oligopaint library was synthesized by Twist Biosciences. All underlying pipelines, scripts, and oligo tables are provided as a Zenodo repository, see Data availability.

#### PCR amplification of oligopaint library

The synthesized oligopaint library was resuspended in 10 mM Tris buffer (pH 8.0) to a final concentration of 20 ng/μl. The oligopaint library amplification was performed by PCR, using following reagents: 1 μl 10 μM universal forward primer, 1 μl 10 μM reverse primer, 1 μl dNTPs (New England BioLabs, N0447S), 0.5 μl Q5 High‐Fidelity DNA Polymerase (New England BioLabs, M0491S), 5 μl Q5 Buffer, 5 μl GC enhancer, 11 μl ddH_2_O, and 0.5 μl oligopaint library. The PCR cycles were as follows: incubation at 98°C for 3 min, followed by twelve cycles of 20 s at 98°C, 15 s at 53°C, and 15 s 72°C, with a final elongation for 1 min at 72°C.

#### Cleaning after oligopaint library amplification

The amplified oligopaint library was cleaned using DNA Clean & Concentrator‐5 kit (Zymo Research, DCC‐5). 25‐μl PCR product was mixed with 175 μl Zymo DNA binding buffer, transferred to a Zymo DCC‐5 column, and spun at 16,000 *g* for 1 min. Next, 200 μl DNA wash buffer was added to the column and centrifuged at 16,000 *g* for 1 min. The wash step was repeated. The flow‐through was discarded, and the column was spun at 16,000 *g* for 1 min once more. The column was transferred to a clean 1.5‐ml tube, and 30 μl ddH_2_O was added. The column was incubated at room temperature for 1 min and centrifuged at 16,000 *g* for 1 min. The concentration was measured using a NanoDrop device.

#### PCR amplification of oligopaint probes

For probe amplification, a working concentration of 2 ng/μl of the oligopaint library was prepared. The oligopaint probes were amplified using PCR. The amplification mix was prepared as follows: 2.5 μl 10 μM gene‐specific forward primer, 2.5 μl 10 μM reverse primer with T7 promoter sequence (T7 promoter sequence: TAATACGACTCACTATAGGG), 2.5 μl dNTPs, 0.5 μl Q5 High‐Fidelity DNA Polymerase, 10 μl Q5 Buffer, 10 μl GC enhancer, 19.5 μl ddH_2_O, and 2.5 μl oligopaint library. The PCR cycles were as follows: incubation for 5 min at 98°C, followed by 43 cycles of incubation at 98°C for 30 s, 58°C for 30 s, and 72°C for 15 s, with a final elongation for 5 min at 72°C.

#### Cleaning after oligopaint probe amplification

The oligopaint probes were cleaned using the DNA Clean & Concentrator‐5 kit (Zymo Research, DCC‐5). 50 μl PCR product was mixed with 350 μl Zymo DNA binding buffer, transferred to a Zymo DCC‐5 column, and spun at 16,000 *g* for 1 min. Afterward, 200 μl DNA Wash Buffer was added to the column and centrifuged at 16,000 *g* for 1 min. The wash step was repeated. The flow‐through was discarded, and the column was spun at 16,000 *g* for 1 min. The column was transferred to a clean 1.5‐ml tube, and 11 μl ddH_2_O was added. The samples were incubated at room temperature for 1 min and centrifuged at 16,000 *g* for 1 min.

#### T7 Reaction—*in vitro* transcription and fluorophore attachment

The amplified probe set was *in vitro* transcribed using a HiScribe T7 Quick High Yield RNA Synthesis Kit (New England BioLabs, E2050S). Each probe set was prepared as follows: 10 μl PCR product, 10 μl NTP buffer mix (from HiScribe kit), 2 μl T7 Polymerase mix (from HiScribe kit), 0.5 μl Recombinant RNasin (Promega, N2511), and 7.5 μl ddH_2_O. The samples were incubated at 37°C in the PCR machine for 4 h. For reverse transcription and fluorophore attachment, M‐MuLV Reverse Transcriptase (New England BioLabs, M0253L) was used. Each probe was prepared as follows: 7 μl dNTPs (New England BioLabs, N0447S), 7 μl 10× M‐MuLV Buffer, 10 μl of 100 μM A594‐labeled forward primer, 1.2 μl M‐MuLV enzyme, 1.4 μl recombinant RNasin, 13.4 μl nuclease‐free water, and 30 μl RNA from the T7 reaction. The reaction mix was incubated at 50°C for 2 h.

#### Cleaning of the oligopaint probes

To obtain ready‐to‐use oligopaint probes, the reverse transcription products were cleaned up using DNA Clean & Concentrator‐25 kit (Zymo Research, DCC‐25). 140 μl Oligo binding buffer (Zymo Research, D4060‐1‐40) was added to the reverse transcription reaction product, and the sample was mixed. To each sample, 560 μl of 96% ethanol was added and the sample was mixed. The solution was transferred into a Zymo DCC‐25 column and spun at 16,000 *g* for 1 min. The flow‐through was discarded. Afterward, 750 μl DNA Wash Buffer was added on the column and spun at 16,000 *g* for 1 min. The wash step was repeated. The flow‐through was discarded, and the column was transferred to a clean 1.5‐ml tube. DNA was eluted in 30 μl ddH_2_O, and the concentration was measured using a NanoDrop device. The concentration of all purified probes was higher than 3,900 ng/μl.

#### Sample permeabilization

Animal caps of fixed embryos were permeabilized in 0.5% Triton X‐100 in PBS for 15 min. The animal caps were washed once with 1 ml PBST for 2 min and subsequently treated with 0.1 M HCl for 5 min. The samples were washed twice with 1 ml 2× saline sodium citrate buffer with 1% Tween‐20 (2 × SSCT). Subsequently, the samples were washed with 2 × SSCT + 50% formamide solution for 2 min in room temperature, and once with 2xSSCT+50% formamide at 60°C for 20 min.

#### Sample denaturation and hybridization

Liquid was removed and hybridization mix was added, consisting of: 50 μl formamide, 25 μl 4× hybridization buffer (40% dextran sulfate, 8 × SSC, 0.8% Tween‐20), 4 μl 10 μg/μl RNase A, 10 μM oligopaint probes labeled with Alexa 594, and ddH_2_O added to reach a total volume of 100 μl. Samples were denatured at 90°C for 3 min and hybridized over the following night at 37°C.

#### Post‐hybridization washes

The samples were washed four times with 2 × SSCT at 60°C for 5 min. 2 × SSCT was added and incubated for 5 min at room temperature twice. Before proceeding with the immunofluorescence protocol, the samples were washed three times for 5 min in PBST. These procedures were followed by the steps described in the immunofluorescence section.

### Instantaneous structured illumination microscopy (instant‐SIM)

Microscopy data from live whole embryos and inhibitor‐treated, fixed cells were recorded using a VisiTech iSIM high‐speed super‐resolution confocal microscope based on the instant‐SIM principle (York *et al*, 2013). The microscope was built on a Nikon Ti2‐E stand. A Nikon Silicone Immersion Objective (NA 1.35, CFI SR HP Plan Apochromat Lambda S 100XC Sil) was used for live imaging, and a Nikon Oil Immersion Objective (NA 1.49, CFI SR HP Apo TIRF 100XAC Oil) was used for fixed cell imaging. Excitation lasers at 405, 488, 561, and 640 nm were used, and illumination and acquisition settings were kept constant across all samples of a given experimental repeat. Color channels were recorded on two cameras simultaneously for increased speed during live imaging, and sequentially to avoid cross‐talk during fixed cell imaging.

### Stimulated emission double depletion microscopy

Microscopy data from animal caps of fixed whole embryos were recorded using a custom‐built STEDD microscope, as previously described (Zhang *et al*, 2019). The STEDD principle allows suppression of low‐frequency image components as well as out‐of‐focus light and aberrant signal from reexcitation effects (Gao & Nienhaus, 2017; Gao *et al*, 2017). Here, STEDD‐resolved images were recorded using excitation by a 640 nm pulsed laser (675/55 nm (center/width) detection band‐pass filter), depletion by a titanium‐sapphire depletion laser tuned to 779 nm, and focused through an oil‐immersion objective (HCX PL APO CS 100×/1.46, Leica). The confocal image was acquired in an additional scan in the same focal plane, using a 473 nm excitation laser (520/50 nm (center/width) detection band‐pass filter) without additional depletion.

### Two‐ and three‐color stimulated emission depletion microscopy

Data from animal caps were recorded with a Leica TCS SP8 STED microscope with a 775‐nm depletion line and a white light laser with adjustable emission wavelengths, using a motorized‐correction 93× NA 1.30 glycerol objective (HC PL APO 93×/1.30 GLYC motCORR), two HyD detectors for two‐color detection, and an additional photomultiplier tube (PMT) detector for three‐color detection.

Two‐color detection based on the fluorophores STAR RED and STAR ORANGE used the following settings: color channel 1—excitation wavelength 638 nm, detection window 648–750 nm, detector gating 0.9–12 ns; color channel 2—excitation wavelength 590 nm, HyD detector window 600–630 nm, detector gating 0.3–9 ns. The STED depletion was set to 100% 3D‐STED. Two‐color detection based on the fluorophores Alexa 594 and Alexa 647 used the following settings: color channel 1—excitation wavelength 594 nm, HyD detector window 604–640 nm, detector gating 0.7–12 ns; color channel 2—excitation wavelength 640 nm, HyD detector window 650–720 nm, detector gating 0.3–9 ns. The STED depletion was set to 25% 3D‐STED.

Three‐color detection was based on the DNA stain JF646‐Hoechst, the fluorophore Alexa 594, and the large Stokes shift fluorophore STAR 520 SXP, using the following settings: channel 1—excitation wavelength 640 nm, HyD detector window 661–741 nm, time gating window 1.5–9 ns; channel 2—excitation wavelength 590 nm, HyD detector window 600–660 nm, time gating window 1–9 ns; channel 3—excitation wavelength 514 nm, PMT detector window 540–600 nm, ungated detection. The STED depletion power was set to 25% 3D‐STED.

### Image analysis—general overview

Image analysis was carried out using FIJI (Schindelin *et al*, 2012) and CellProfiler (McQuin *et al*, 2018) combined with Python for data handling, as well as MatLab combined with the Open Microscopy Environment BioFormats importer functionality provided by bfmatlab (Goldberg *et al*, 2005). The following provides an overview of the key image analysis steps carried out for all figures included in this study. The underlying raw image data and all pipelines and scripts are provided as Zenodo repositories indicated in each section.

### Image analysis—relationship of Pol II phosphorylation and cluster morphology in STEDD microscopy data

These analyses (Fig 2) were carried out in MatLab. The raw data and analysis scripts are available via Zenodo, see Data availability. Nuclear segmentation masks were obtained by Gaussian blur (*σ* = 1.2 μm) and Otsu thresholding of the Pol II Ser5P channel. Pol II Ser5P clusters and Pol II Ser2P spots were segmented by local background subtraction (Gaussian blur image with *σ* = 0.4 μm subtracted), followed by global robust background thresholding (0.5 and 0.25 Standard Deviations), respectively. For each Pol II Ser5P cluster, mean intensity, area, and solidity were extracted. For Pol II Ser2P spots, only the mean intensity was extracted due to the lower confocal resolution relative to the STEDD data.

### Image analysis—changes in Pol II Ser2P upon flavopiridol treatment of whole embryos

These analyses (Appendix Fig S5) were carried out in MatLab. The raw data and analysis scripts are available via Zenodo, see Data availability. Nuclei were segmented by Otsu thresholding of the blurred (Gaussian kernel with *σ* = 0.3 μm) and background‐subtracted (Gaussian kernel with *σ* = 5 μm) Pol II Ser5P channel. Cytoplasmic background was subtracted using secondary masks, which were obtained by dilation to 1.0 and 1.5 μm of the primary nuclear masks.

### Image analysis—Pol II phosphorylation and cluster morphology in instant‐SIM data

These analyses (Appendix Figs S3, S14 and S15) were carried out in CellProfiler. The raw data and analysis pipeline and scripts are available via Zenodo, see Data availability. Z‐stacks were maximum‐intensity‐projected in FIJI, including a range of 25 slices visually chosen for best image quality from a given z‐stack. The further 2D analysis was implemented as a CellProfiler pipeline. Specifically, a two‐step approach was used, where first cell nuclei and subsequently Pol II clusters inside nuclei were segmented based on the Pol II Ser5P signal. Nuclei segmentation masks were obtained by global Otsu thresholding. Cytoplasmic masks were generated by outward dilation (25 pixels) from the nuclear masks. Pol II clusters inside nuclei were obtained by enhancing the Pol II Ser5P channel (speckle enhancement) and global robust background thresholding (5.5 standard deviations). For each cluster, the mean Pol II Ser5P and Pol II Ser2P intensities (cytoplasmic background subtracted on per‐nucleus basis), cluster area, and cluster solidity were extracted. The mean intensity of Pol II Ser5P and Pol II Ser2P in the nuclei, cytoplasm, and in single clusters was measured. The geometric properties—solidity, area—were measured for each cluster. All clusters smaller than four pixels were discarded. Further data processing and graph preparation were done in Python. Data from fixed cells were analyzed in the same way, except that, as a first step, the additional color channels with DNA and Histone 3 serine 28 phosphorylation (H3Ser28P, used to detect cells in prophase) signal were used to establish Otsu‐threshold masks for nuclear segmentation and prophase exclusion, respectively. In the robust background segmentation of Pol II Ser5P clusters, 6.5 standard deviations were chosen for zebrafish primary cell cultures, 8 standard deviations for THP‐1 cell cultures. A comparison of our analysis based on two‐dimensional, maximum‐intensity‐projected images with an analysis of full three‐dimensional stacks showed a good correlation between both approaches (Appendix Fig S2A and B).

### Image analysis—analysis of Pol II Ser5P / Ser2P colocalization in two‐color STED data

These analyses (Appendix Fig S6) were carried out in MatLab. The raw data and analysis pipeline and scripts are available via Zenodo, see Data availability. Images were two‐dimensional and contained one nucleus per image. The nucleus was segmented by Otsu thresholding of the blurred Pol II Ser5P channel (*σ* = 1.2 μm). Pol II Ser5P and Pol II Ser2P foci were segmented by application of a robust background threshold within the segmented nucleus (2.0 and 3.0 standard deviations, respectively). The Pol II Ser5P channel was background‐subtracted prior to segmentation (Gaussian blur with *σ* = 0.3 μm). Mean intensities of clusters were calculated using Ser5P segmentation masks and normalized against whole nucleus median intensity on a per‐nucleus level. Overlap was quantified by use of the Pol II Ser5P and Pol II Ser2P segmentation masks, and quantified in terms of percentage of all Pol II Ser2P mask pixels in a given nucleus.

### Image analysis—Pol II Ser5P clusters and Pol II Ser2P spots after hexanediol treatment

These analyses (Fig 3) were carried out in MatLab. The raw data and analysis pipeline and scripts are available via Zenodo, see Data availability. Z‐stacks were maximum‐intensity‐projected in FIJI, including a range of 25 slices visually chosen for best image quality from a given z stack. The further analysis was carried out in MatLab. Nuclei in a given z projection were segmented by Otsu thresholding of the blurred Pol II Ser5P channel (*σ* = 1.2 μm). Pol II Ser5P clusters and Pol II Ser2P foci were segmented by application of a robust background threshold within the segmented nucleus (4 and 4 standard deviations, respectively). Both channels were background‐subtracted prior to segmentation (Gaussian blur with *σ* = 0.5 μm). Area and solidity of a given object were calculated from Pol II Ser5P and Pol II Ser2P segmentation masks.

### Image analysis—H3K27ac and DNA distribution with respect to Pol II Ser5P clusters

These analyses (Fig 4D and E) were carried out in MatLab. The raw data and analysis pipeline and scripts are available via Zenodo, see Data availability. STED reexcitation signal in the DNA channel was reduced by subtracting a Gaussian‐blurred (*σ* = 0.1 μm) image recorded with only the STED laser activated. Images were two‐dimensional and contained one nucleus per image. The nucleus was segmented by Otsu thresholding of the blurred DNA channel (*σ* = 1.2 μm). Pol II Ser5P foci were segmented by application of a robust background threshold within the segmented nucleus (2.5 standard deviations). The Pol II Ser5P channel was background‐subtracted prior to segmentation (Gaussian blur with *σ* = 2 μm). Mean intensities were calculated from Pol II Ser5P segmentation masks and normalized against whole nucleus median intensity on a per‐nucleus level.

### Image analysis—oligopaint signals in relation to Ser5P clusters

These analyses (Fig 4F and G) were carried out in MatLab. The raw data and analysis pipeline and scripts are available via Zenodo, see Data availability. All analysis operations were carried out under consideration of three‐dimensional organization. Each image stack typically contained several nuclei, which were segmented by Otsu thresholding of the blurred (Gaussian kernel with *σ* = 1 μm) and background‐subtracted (Gaussian kernel with *σ* = 10 μm) Pol II Ser5P channel. Pol II Ser5P clusters and oligopaint‐labeled regions were segmented by application of a robust background threshold within the segmented nuclei (2 and 6 standard deviations, respectively). The Pol II Ser5P channel was background‐subtracted (Gaussian kernel with *σ* = 3 μm) prior to segmentation. The oligopaint channel was background‐subtracted (Gaussian kernel with *σ* = 5 μm) and smoothed (Gaussian kernel with *σ* = 0.1 μm) prior to segmentation. Mean intensities were calculated from oligopaint segmentation masks and normalized against whole nucleus median intensity on a per‐nucleus level. The distance between oligopaint‐labeled regions and Pol II Ser5P clusters was assessed for each oligopaint‐labeled region, using the Euclidean distance to the nearest Pol II Ser5P cluster. Euclidean distance was calculated between centroids of segmentation masks.

### Statistics

Box plots conform to standard practice (median, quartiles as boxes, range as whiskers, outliers removed outside of 1.5 times interquartile range extension). Statistical significance was indicated for differences of mean or median (as indicated) relative to the control or comparison condition, two‐tailed permutation test; *, **, and *** indicate *P* < 0.05, *P* < 0.01, and *P* < 0.001, respectively; and n.s. indicates no statistically significant difference (*P* ≥ 0.05). Significance levels were Bonferroni‐corrected for multiple comparisons (division by number of comparisons), leading to lowered significance levels as indicated. The permutation test is based on a computational resampling procedure, which we sampled to an accuracy of *P* = 0.0001, lower values are stated as *P* < 0.0001.

### Lattice model

#### Lattice kinetic Monte Carlo model

We describe the Pol II cluster morphologies observed in zebrafish experiments using a simple physical model, which is limited to the most essential components: particles corresponding to a Pol II Ser5P‐rich component (in the following abbreviated as Pol II particles) and linear polymer chains with different subregions corresponding to chromatin. To obtain spatial configurations of this system, we used a rejection‐free lattice kinetic Monte Carlo (LKMC) algorithm. LKMC algorithms, generally speaking, are suited to simulate coarse‐grained stochastic non‐equilibrium systems. The rejection‐free algorithm (Appendix Fig S9A) is similar to the Gillespie algorithm (Gillespie, 1977). At the beginning of a simulation, by checking the system configuration and nearest neighbors of every particle within the system, a rate catalog with all possible transitions is created, providing also the total system rate as the sum of all transition rates. This initial cataloging step is followed by a Monte Carlo (MC) routine that is repeated *N* times. During each step, one of the previously defined transitions is randomly selected while associating transitions with a higher rate with a higher likelihood of occurrence. The transition is then performed and changes the system state. This is followed by a local update of the possible transitions in the affected lattice area, the total rate of the system, and the system time. This simulation paradigm has been used to model surface catalysis processes (Hoffmann *et al*, 2014) and slip‐link DNA systems with DNA polymers and ring proteins (Miermans & Broedersz, 2020). The initialization of the simulations proceeds similar to the latter work, but instead of ring proteins uses the Pol II particles in addition to the polymers.

#### Initial configuration

Chromatin is modeled as a connected polymer chain with different internal states: inactive (black), regulatory (blue), and active (gray). Exclusion from occupied volume is assumed, so that chains can only undergo a limited type of moves that maintain chain connectivity. Pol II particles (single lattice sites, red) can freely diffuse in space and interact with different affinities *w_i_* with the chromatin polymer and other Pol II particles. The different interspecies affinities of Pol II particles to specific subregions of a chain allow us to study the formation of Pol II clusters in the framework of microphase separation. At the beginning of a given simulation, the Pol II particles are randomly distributed on the 25 × 25 lattice. The chromatin polymer was placed in different initial configurations (e.g., single chain, cross of four chains, four parallel chains, four chains organized as random walks).

The monomers making up chromatin chains were assigned to the different colors, giving contiguous sections of black polymer (number of monomers: *N*_IC_), blue monomers (*N*_RC_), and gray monomers (*N*_AC_). The number of red particles (*N*_S5P_) can be varied.

#### Pol II particle and polymer move set

After initialization and each time step, the rate catalog is updated. To find all possible transitions of the system for the rate catalog, we first have to define the allowed move set for every species. The Pol II particles are allowed to move to one of its eight nearest neighbors in one MC step (Appendix Fig S9D, left). The polymer is simulated as a connected and self‐avoiding chain. We therefore use the common Verdier‐Stockmayer move set, consisting of end‐bond flip, kink‐jump, and a crankshaft move (Appendix Fig S9D, right) (Verdier & Stockmayer, 1962; Hilhorst & Deutch, 1975; Miermans & Broedersz, 2020). Movements to positions outside the lattice are not considered.

The end‐bond flip can occur only for the first or last monomer of the polymer and moves the first/last monomer to any lattice site neighboring the second/second‐last particle of the polymer, changing the angle between the old and new positions by 90 degrees. For a kink jump, a monomer is moved to the opposite side of a corner formed by the preceding monomer, the monomer itself, and the subsequent monomer. To reach ergodicity of the system, a third move, called the crankshaft move, is added (Hilhorst & Deutch, 1975). While the end‐bond flip and the kink jump move only one monomer, the crankshaft move changes the position of two successive monomers.

#### Rate catalog

To draw up the rate catalog, we first browse all Pol II‐particle positions within the lattice and test the occupation of its eight nearest neighbors. Those that are not occupied by the same species are possible directions for transitions within the system. Together with the defined rate coefficient, *k_p_* = 0.1, and the Arrhenius equation,

$$k=k_{p}\cdot e^{‐(E_{2}‐E_{1})},$$

the rate for each transition can be determined. The energies before (*E*_1_) and after (*E*_2_) are stated in units of *k_B_T*, where *T* is the system's temperature, and are determined under consideration of the different interspecies affinities between the swapped particles and their nearest neighbors (Appendix Fig S9C). Where a nearest neighbor position is located outside the lattice, no energy contribution is added for that neighbor. For every Pol II‐particle transition, the old and new position and also the rate for the transition are added to the rate catalog. The transition rate is also added to the overall system rate *k*_total_.

As a second step, the same procedure is carried out for moves of the polymer chain. Initially, the position within the polymer and also the position of the previous and subsequent monomers are checked. Depending on the position and configuration, only certain movements of the monomer are possible. As with the Pol II particles, it must then be checked whether the possible new position resulting from the move is already occupied by another monomer. If this is not the case, the Arrhenius equation and the rate coefficient for a single move (same as *k*_kink_) are used to determine the rate of the transition. Since the crankshaft move affects two monomers at the same time, this coefficient is smaller compared to the other moves, *k*_crank_ = *k*_kink_⋅log 4/3 (Miermans & Broedersz, 2020). The polymer transitions are also added to the rate catalog. In addition to the positions and rates, the specific type of movement is added. Furthermore, for the crankshaft move both positions before and after the respective move are saved. Now all required properties of the system are defined and the main part of the Monte Carlo simulation can start.

#### Perform move (MC‐Step)

In this section, the core iteration step of the algorithm is described. At first, we draw a transition out of the previously defined rate catalog. This is done using a uniformly distributed random number *r*_1_ from the interval (0,1], the total rate *k*_total_, and the tower‐sampling method (Appendix Fig S9E). The transition is chosen using the quantity k* = *r*_1_⋅*k*_total_. In particular, by looping through all transitions within the catalog and summing up each individual rate *k_i_*, we identify a transition *j* with rate *k_j_* such that the relation

$$\sum_{i}^{j‐1}k_{i}<k^{*}\leq \sum_{i}^{j}k_{i}$$

is fulfilled. The chosen transition is then performed and the system lattice is updated. The system time is also updated by adding

$$\Delta t=‐\frac{\log(r_{2})}{k_{\mathrm{total}}}$$

with a second random number *r*_2_ from (0,1] to the current system time *t* (Miermans & Broedersz, 2020). This routine is performed *N* times.

#### Local update

At the end of each of the above iteration steps, the system rate catalog has to be updated. In the simplest approach, we delete the previous rate catalog and completely recalculate it. While formally correct, more computationally efficient alternative approaches are available (Miermans & Broedersz, 2020). Position changes in the simulation are only made in a confined area surrounding the particle that is moved. Accordingly, only the transitions falling within this area need to be updated. We therefore determine all transitions leading to a position within this area, or whose origin is within this area and update only these transitions. Since the longest transition within the system is performed by the crankshaft move with a distance of two lattice sites, the local update has to be performed in the region [*x* ± 4, *y* ± 4] (Appendix Fig S9F).

#### Parameter adjustment

Besides the self‐affinity of Pol II particles, *w*_S5P‐S5P_ (interaction energy measured in units of *k_B_T*; Fig 5), we also adjusted the number of Pol II particles, *N*_S5P_, and Pol II‐particle affinity to regulatory chromatin, *w*_RC‐S5P_. To adjust *N*_S5P_, we add a single polymer chain of length *L*_Polymer_ = 20 with black and blue subregions to the simulation (Appendix Fig S10A). We use the previously determined affinity *w*_S5P‐S5P_ = −0.35, and assign *w*_RC‐S5P_ = −0.5 as a preliminary value. We then varied the number of Pol II particles (*N*_S5P_ = 10, 25, 50, 100, 200). Since we planned to perform simulations with four or more polymers and different lengths of the blue regions, we choose *N*_S5P_ = 100 so as to provide enough material for cluster formation (Appendix Fig S10A). To assess the preliminary *w*_RC‐S5P_ = −0.5 value, we performed simulations containing a single polymer chain (*L*_Polymer_ = 8) that consists only of blue subregions, using again *w*_S5P‐S5P_ = −0.35 and the previously determined amount of Pol II particles. As we vary *w*_RC‐S5P_ = −0.1, −0.3, −0.5, −0.7, −1.0, we find that the only parameter for which not the whole polymer is covered by Pol II particles (blue still visible) is *w*_RC‐S5P_ = −0.1 (Appendix Fig S10B). For all tested values, cluster formation occurs, so that we continued using the preliminary value *w*_S5P‐S5P_ = −0.5, which falls in the middle region of the tested interval.

Although a main aim of our model was to describe the physical mechanism of cluster formation, and not to reproduce experimental data in absolute numbers, we briefly comment on how biologically realistic the assigned parameter values are. The affinities, which determine much of the model behavior, are closely comparable to those used in our previous work (Hilbert *et al*, 2021), where we describe the organization of euchromatin as a result of RNA polymerase II transcriptional activity. Also similar to this work, we can approximately set the distance between two lattice sites equivalent to the edge length of a pixel in our instant‐SIM microscopy images (≈ 65 nm). The area of large simulated clusters (≈ 100 lattice sites, see Fig 6B) thus corresponds to ≈ 0.43 μm^2^, which agrees very well with areas of large clusters seen in our experiments (Fig 2C). Further, by assigning regulatory and active regions of similar length to the chromatin chains, the relative lengths seen in our ChIP‐seq analysis are reproduced (Fig 4A and B).

#### Image processing

Synthetic microscopy images were obtained by converting lattice distributions of red monomers, gray polymer regions, and blue polymer regions into matrices with values 0 (unoccupied) or 1 (occupied), which were blurred with a Gaussian kernel (*σ* = 1, in units of pixels). For these synthetic color channels, artificial detector noise was added in the form of Poisson‐distributed random numbers (mean *λ* = 5, divided by 100 before addition). Objects corresponding to Pol II Ser5P clusters were obtained from the blurred distributions of red particles using a segmentation threshold of 0.35 followed by connected‐component analysis (8‐connectivity, components with less than 10 pixels were excluded). Distributions of area, solidity, and intensity of these objects were obtained by sampling simulations at different time points (ergodic sampling). The numerical simulation and the analysis of synthetic images were carried out using Python.

## Author contributions

Conceptualization: AP, TK, GUN, VZ, LH; Investigation: AP, TK, WZ, RP, IM, AN, MS, LH; Methodology: AYK; Formal analysis: AP, TK, RP, LH; Software: AP, TK, RP, LH; Supervision: GUN, VZ, LH; Writing—original draft: AP, TK, LH; Writing—review & editing: AP, TK, WZ, RP, IM, GUN, VZ, LH.

## Conflict of interest

The authors declare that they have no conflict of interest.

## Supporting information



Appendix

## Data Availability

The raw data, analysis scripts, and simulation code used in this study are provided for public access via Zenodo under the DOIs stated below.
Data set and derived images for Fig 1: https://doi.org/10.5281/zenodo.4974360 Data set and analysis scripts for Fig 2: https://doi.org/10.5281/zenodo.4973061 Data set and analysis scripts for Fig 3A–D: https://doi.org/10.5281/zenodo.4973197 Data set and derived images for Fig 3E and F: https://doi.org/10.5281/zenodo.4973309 Scripts and analysis results for Fig 4A–C, Appendix Figs S7 and S8, and the design and application of the oligopaint library: https://doi.org/10.5281/zenodo.4972246 Data set and scripts for Fig 4D and E: https://doi.org/10.5281/zenodo.4972656 Data sets and scripts for Fig 4F and G. Analysis scripts and extracted image data: https://doi.org/10.5281/zenodo.4973181 Image data for super‐enhancers SE1‐SE6: https://doi.org/10.5281/zenodo.4973507 Image data for super‐enhancers SE7‐SE12: https://doi.org/10.5281/zenodo.4973510 Image data for four genes: https://doi.org/10.5281/zenodo.4973522 Data sets and scripts for the theoretical model, relating to Figs 5, 6, 7 and Appendix Figs S9–S13: https://doi.org/10.5281/zenodo.5203821 Data sets and scripts for Fig 7 and Appendix Figs S3A–C, S14 and S15: https://doi.org/10.5281/zenodo.4972466 Image data used to prepare Appendix Fig S1: https://doi.org/10.5281/zenodo.4974449 Data set and scripts for Appendix Fig S2: https://doi.org/10.5281/zenodo.4983658 Data set and scripts for Appendix Fig S3D–G: https://doi.org/10.5281/zenodo.5230667 Data set and scripts for Appendix Fig S4: https://doi.org/10.5281/zenodo.5229601 Image data and scripts for Appendix Fig S5: https://doi.org/10.5281/zenodo.5229988 Data set and scripts for Appendix Fig S6: https://doi.org/10.5281/zenodo.5229952 Data set and scripts for Appendix Fig S16: https://doi.org/10.5281/zenodo.4972011
