# Supplementary material for: RNA polymerase II clusters form in line with surface condensation on regulatory chromatin
Source: Mol Syst Biol. 2021 Sep 27;17(9):e10272. doi: 10.15252/msb.202110272 (PMC8474054; doi:10.15252/msb.202110272)
Supplement: Supplementary file 1 — Appendix [file MSB-17-e10272-s002.pdf]

Appendix

RNA polymerase II clusters form in line with surface condensation on regulatory chromatin

Agnieszka Pancholi[1,2,*], Tim Klingberg[3,4,*], Weichun Zhang[5,6], Roshan Prizak[2], Irina Mamontova[2], Amra Noa[2], Marcel Sobucki[2], Andrei Yu Kobitski[5], Gerd Ulrich Nienhaus[2,5,6,7,@], Vasily Zaburdaev[3,4,@], Lennart Hilbert[1,2,@]

[1]Zoological Institute, Department of Systems Biology and Bioinformatics, Karlsruhe Institute of Technology, 76131 Karlsruhe, Germany; [2]Institute of Biological and Chemical Systems – Biological Information Processing, Karlsruhe Institute of Technology, 76344 Eggenstein-Leopoldshafen, Germany; [3]Department of Biology, Friedrich-Alexander-Universität Erlangen-Nürnberg, 91058 Erlangen, Germany; [4]Max-Planck-Zentrum für Physik und Medizin, 91058 Erlangen, Germany; [5]Institute of Applied Physics, Karlsruhe Institute of Technology, 76128 Karlsruhe, Germany; [6]Institute of Nanotechnology, Karlsruhe Institute of Technology, 76344 Eggenstein-Leopoldshafen, Germany; [7]Department of Physics, University of Illinois at Urbana-Champaign, Urbana, IL 61801, United States of America; *These authors contributed equally; @Corresponding authors: uli@uiuc.edu, vasily.zaburdaev@fau.de, lennart.hilbert@kit.edu

Table of Contents

Fig. S1, page 2
Fig. S2, page 3
Fig. S3, page 4
Fig. S4, page 5
Fig. S5, page 6
Fig. S6, page 7
Fig. S7, page 8
Fig. S8, page 9
Fig. S9, page 10
Fig. S10, page 11
Fig. S11, page 12
Fig. S12, page 13
Fig. S13, page 14
Fig. S14, page 15
Fig. S15, page 16
Fig. S16, page 17

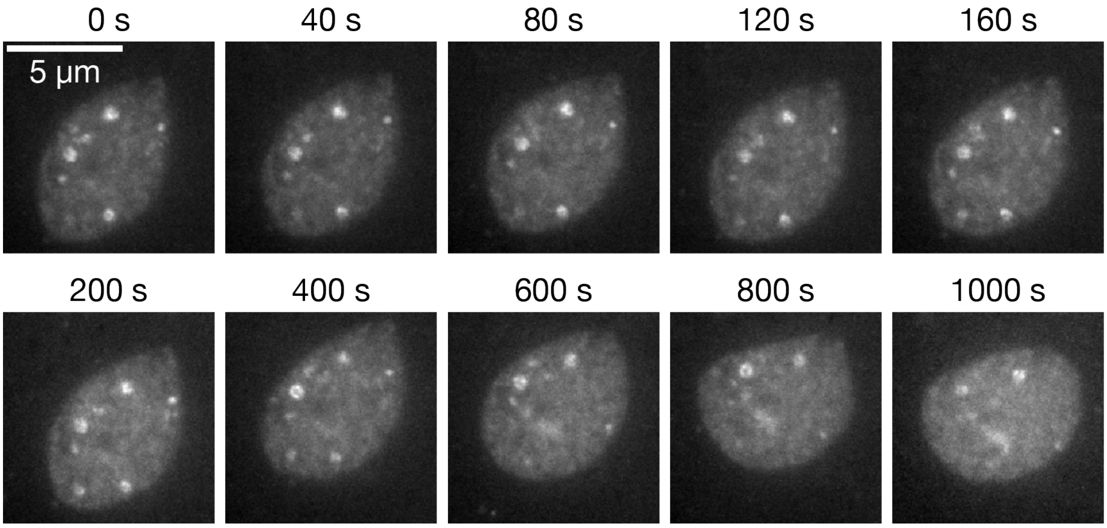

Fig. S1: **Time-lapse imaging of paused Polymerase II clusters.** Clusters were visualized in live zebrafish embryos (sphere stage) using antigen-binding fragments (Fab, Cy3-labeled) of antibodies against the RNA polymerase II carboxy-terminal domain serine 5 phosphorylation. The time-lapse images are maximum-intensity projections from a data set with 40 s time resolution. Images were chosen at selected time points to show short-term and long-term behavior. The same persistence of cluster morphologies was observed in time lapse sequences recorded from three embryos in parallel in the same experiment, and in a second independent repeat of this experiment.

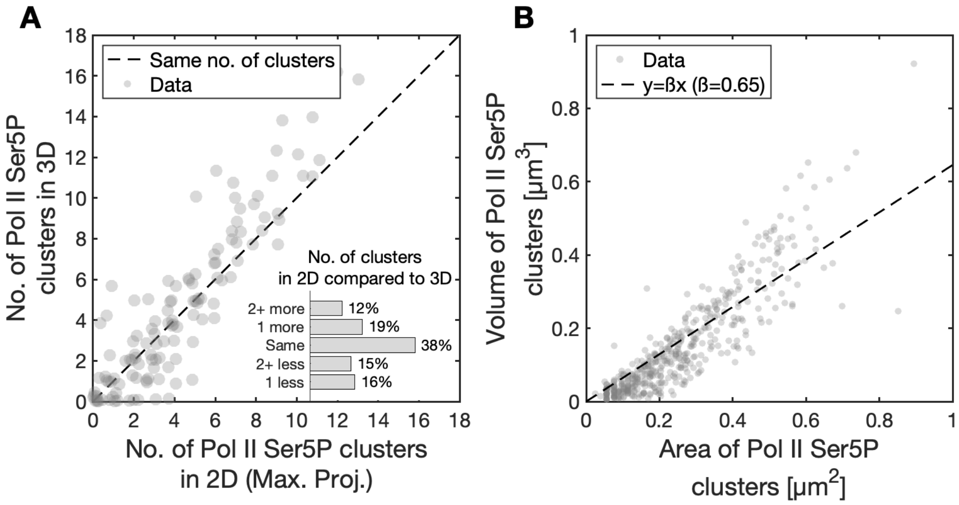

Fig. S2: **Comparison of 3D vs. 2D segmentation and analysis of polymerase clusters.** A) To test whether our analysis of maximum-intensity-projected, two-dimensional (2D) microscopy data is representative of three-dimensional (3D) organization, we compare the number of Pol II Ser5P clusters detected per nucleus in both cases. A plot of the numbers from 2D segmentation based on maximum-intensity projection (x-axis) and from 3D segmentation (y-axis) shows only small deviations from the dashed diagonal line (indicating perfect agreement). In the inset, we show the fraction of nuclei in which the number of clusters in the 2D and 3D methods are the same, differ by just one cluster, or by two and more clusters. The two methods result in a similar number of clusters: 38% of the nuclei have same number, and a total of 72% of nuclei differ in the 2D and 3D clusters at most by one cluster. The clusters were segmented using a global Robust Background Threshold (3 standard deviations for 2D projection and 4.5 standard deviations for the 3D image) on the masked images. B) Comparison of the areas and volumes of the clusters obtained from 2D segmentation and 3D segmentation, respectively. For each nucleus, we obtained a set of common clusters in 2D and 3D by comparing their x, y positions (ignoring the z position of the 3D clusters) and picking those that overlap. For these clusters, a direct comparison is possible, and we can see that volume and area are highly correlated. Overall, this comparison of 3D and 2D Pol II Ser5P clusters shows that 2D segmentation using maximum-intensity projections results in a similar number and size of clusters when compared to a 3D analysis, justifying our approach of using the 2D method for our main analysis.

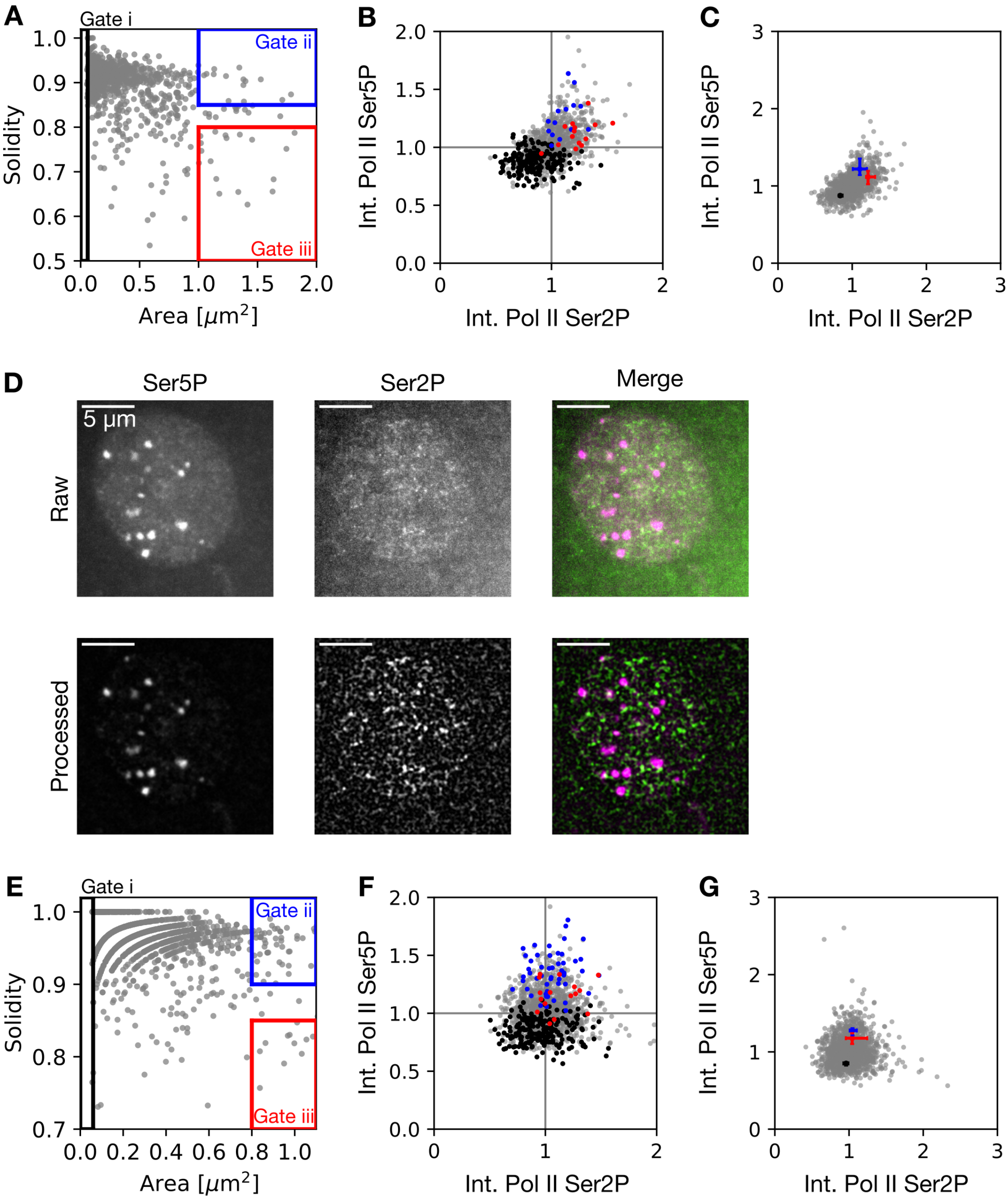

Fig. S3: **Cluster morphology types are also detected in live embryos and fixed embryos injected with antigen-binding fragments.** A) Cluster properties were extracted from images obtained from live zebrafish embryos (sphere stage) using antigen-binding fragments (for details, see Materials and Methods). Area and solidity of the individual clusters, with gates showing type i, ii and iii clusters. B) Phosphorylation levels (serine 2 and serine 5 phosphorylation) of the individual clusters (mean fluorescence intensity). The values are normalized to the median of each analyzed nucleus. Number of nuclei: $n = 109$. Number of clusters: $n = 959$. C) Median Pol II Ser2P and Ser5P intensities with 95% bootstrap confidence intervals (10,000 resamples). D) Representative maximum-intensity projection of a nucleus of an embryo injected with antigen-binding fragments prior to fixation. Raw data were processed by local background subtraction and smoothing. E-G) Same analysis as in panels A-C, applied to image data obtained from fixed embryos injected with antigen-binding fragments. Number of nuclei: $n = 177$. Number of clusters: $n = 1326$.

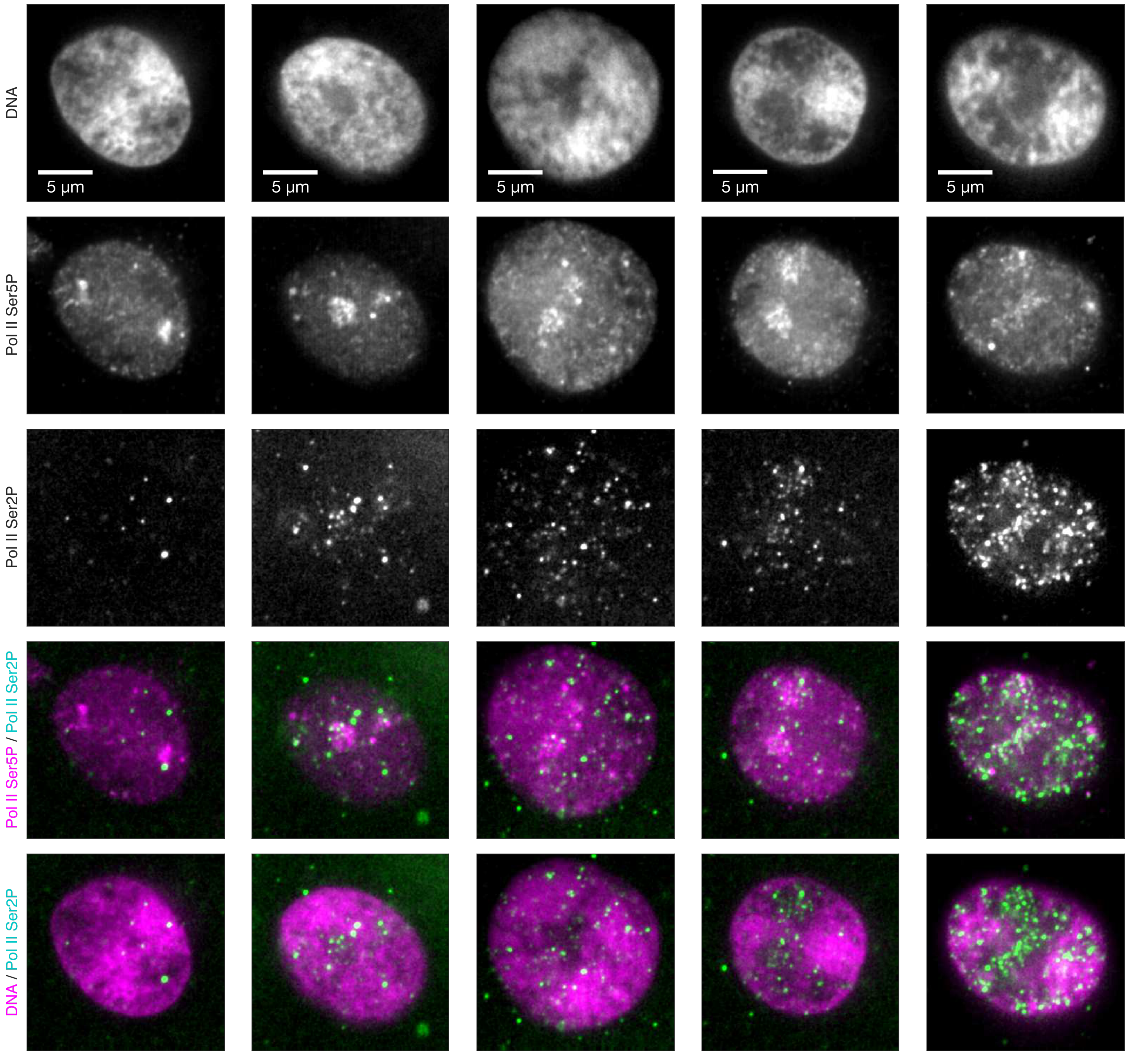

Fig. S4: **Recruited and elongating RNA polymerase II is detected in microRNA miR-430-associated foci.** Micrographs of nuclei in a zebrafish embryo fixed in the oblong stage, showing prominent clusters of recruited RNA polymerase II (Pol II Ser5P) and elongating RNA polymerase II (Pol II Ser2P) associated with the transcription of the miR-430 microRNA cluster. Images recorded by instant-SIM microscopy, single z-sections are shown, Pol II Ser5P and Pol II Ser5P labeled via immunofluorescence (antibody set 1), DNA labeled with Hoechst 33342. Images were sorted by eye, based on the apparent progress of the transcription onset and DNA domain coarsening that follows cell division.

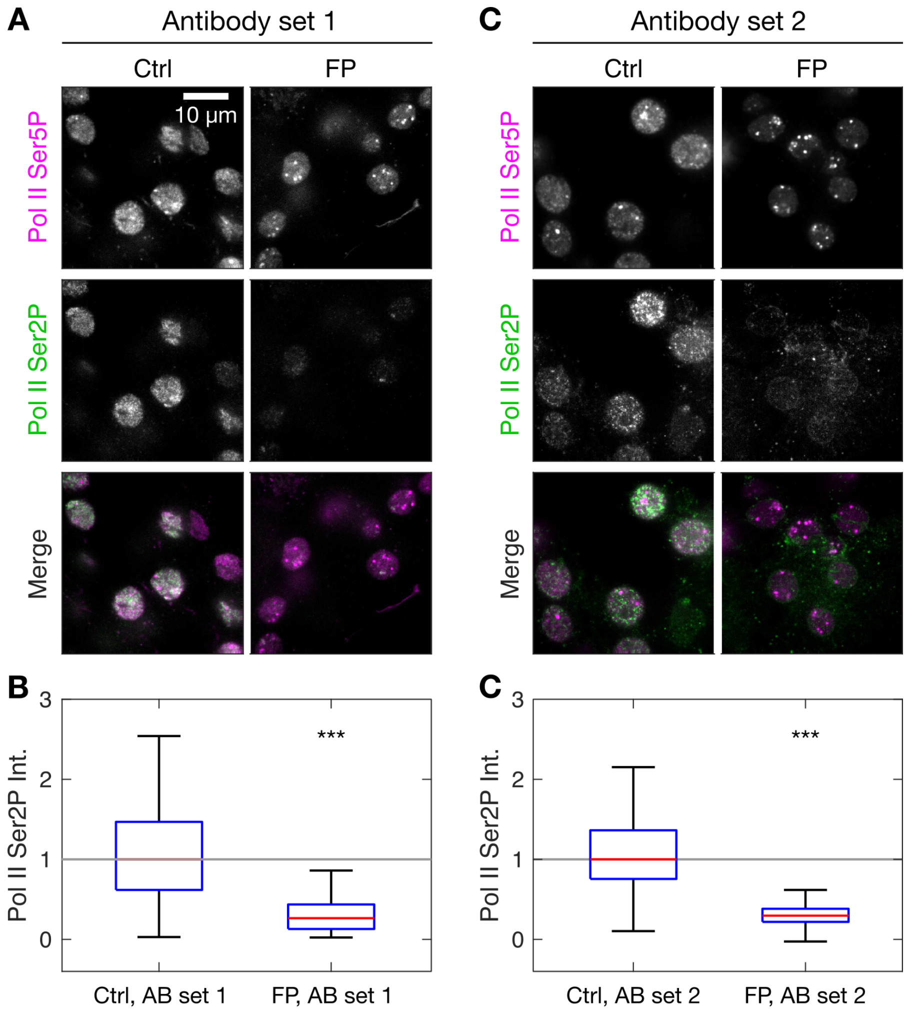

Fig. S5: **Verification of RNA polymerase II serine 2 phosphorylation immunodetection.** A) Representative micrographs of fixed sphere-stage embryos that were untreated (Ctrl) or treated with flavopiridol (FP, 10 μM, 90 min) and labeled by immunofluorescence. Images are single instant-SIM confocal sections. Main antibody set (AB set 1): primary antibodies – mouse IgG anti-Ser5P, rabbit IgG anti-Ser2P; secondary antibodies – anti-mouse IgG STAR RED, anti-rabbit IgG STAR ORANGE. B) Quantification of mean Pol II Ser2P intensities inside nuclei, AB set 1. Segmentation based on Pol II Ser5P channel, intensity values normalized to the median of the control condition. Two-tailed permutation test for Pol II Ser2P intensity differences, *** indicates $P < 0.001$ (P $<$ 0.0001, $n = 409{,}91$ nuclei from five and three embryos). C) Same as panel A, but for an alternative set of primary and secondary antibodies (AB set 2): primary antibodies – rat IgG anti-Ser5P, mouse IgM anti-Ser2P; secondary antibodies – anti-rat IgG Alexa 647, anti-mouse IgM Alexa 594. D) Same quantification as in panel B, but for AB set 2 (P $<$ 0.0001, $n = 106{,}147$ nuclei from two and three embryos.)

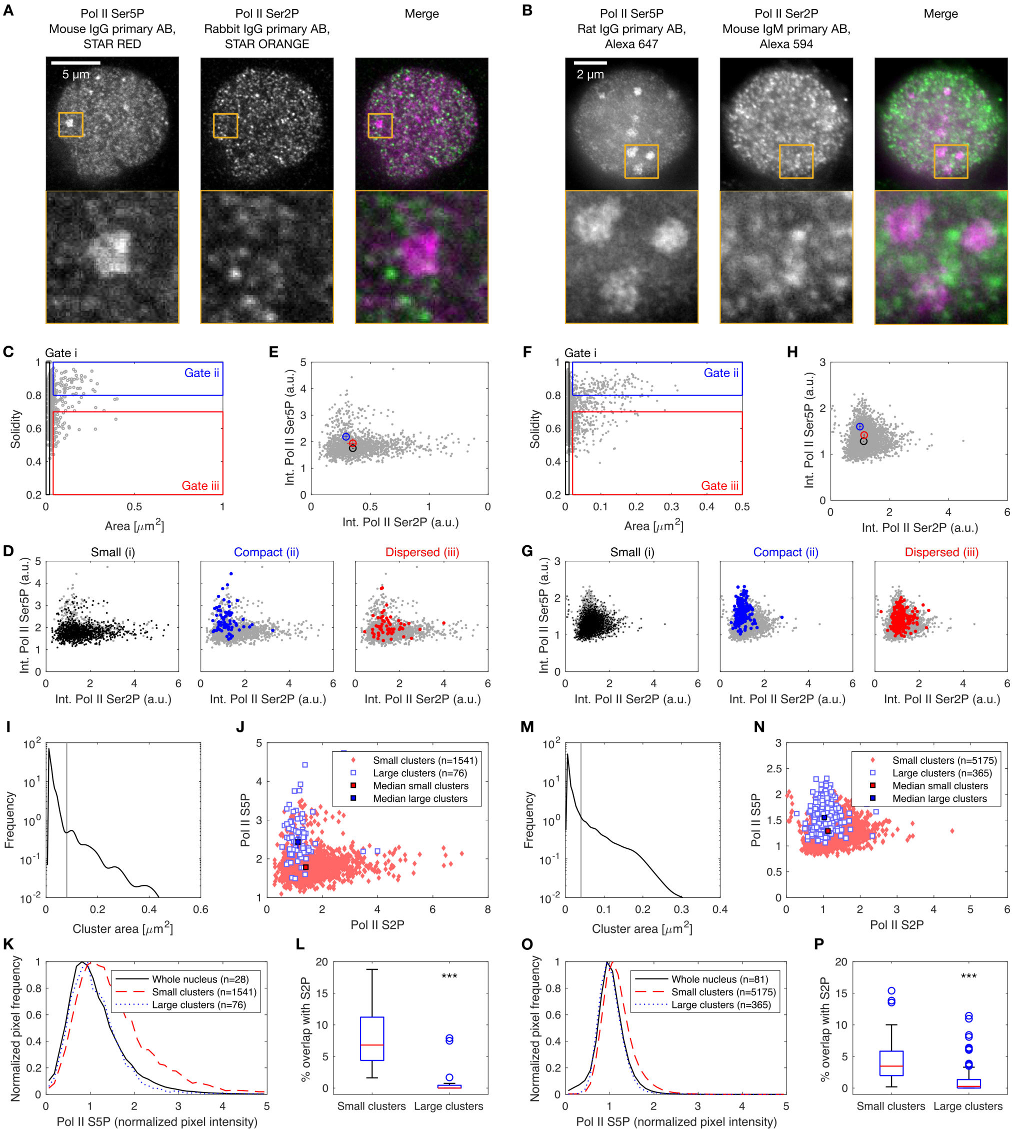

Fig. S6: **Assessment of Pol II Ser5P and Pol II Ser2P localization by 3D-STED with two alternative sets of primary antibodies and fluorophores.** A) Representative two-color 3D-STED section through a nucleus in a fixed sphere-stage zebrafish embryo. Immunofluorescence based on the primary antibody combination used throughout this study for combined Pol II Ser5P and Pol II Ser5P detection (AB set 1, rabbit mono-clonal IgG anti-Pol II Ser2P and mouse mono-clonal IgG anti-Pol II Ser5P) and secondary antibodies conjugated with the STED-optimized fluorophores STAR RED and STAR ORANGE, respectively. 100% 3D-depletion was used. B) Same as panel A for immunofluorescence with an alternative combination of primary antibodies (AB set 2, mouse IgM anti-Pol II Ser2P and rat IgG anti-Pol II Ser5P) and secondary antibodies conjugated with the STED-compatible fluorophores Alexa 594 and Alexa 647, respectively. 3D-STED depletion reduced to 25% to compensate for weaker fluorophore signal. C) Area and solidity of clusters detected in the Pol II Ser5P channel overlaid with gating areas for cluster types i-iii. Maximum area for gate i: $0.02\,\mu\text{m}^2$, minimum area for gates ii and iii: $0.04\,\mu\text{m}^2$, maximum solidity for gate iii: 0.7, minimum solidity for gate ii: 0.8. D) Mean Pol II Ser5P and Pol II Ser2P intensity of all clusters (gray) overlaid with the intensities of those clusters within the gates applied in panel C. E) Median Pol II Ser5P and Pol II Ser2P intensities of clusters within the gates applied in panel C with bootstrap 95%-confidence intervals (10,000 resamples). F-H) Same as panels C-E, but for AB set 2. Modified maximum area for gate i: $0.01\,\mu\text{m}^2$, modified minimum area for gates ii and iii: $0.02\,\mu\text{m}^2$. I) Probability distribution of the area of Pol II Ser5P clusters. A cut-off at $0.08\,\mu\text{m}^2$ divides two populations visible in the logarithmic plot. J) $n = 1541$ small and $n = 76$ large clusters were extracted from images of $n = 28$ nuclei from three embryos. K) Per-pixel Pol II Ser2P intensity levels throughout the entire nucleus and within large and small Pol II Ser5P clusters. L) Percentage of all Pol II Ser2P-positive pixels (robust background threshold with 3.0 standard deviations after background subtraction with range $1.0\,\mu\text{m}$) overlapping with small or large clusters. Two-tailed permutation test for differences in overlap, *** indicates $P < 0.001$, ($P < 0.0001$, $n = 28$ nuclei). M-P) Same as panels I-L but for antibody set 2. Area cut-off $0.04\,\mu\text{m}^2$, $n = 5175$ small and $n = 365$ large clusters from $n = 81$ nuclei from two embryos ($P < 0.0001$, $n = 81$ nuclei).

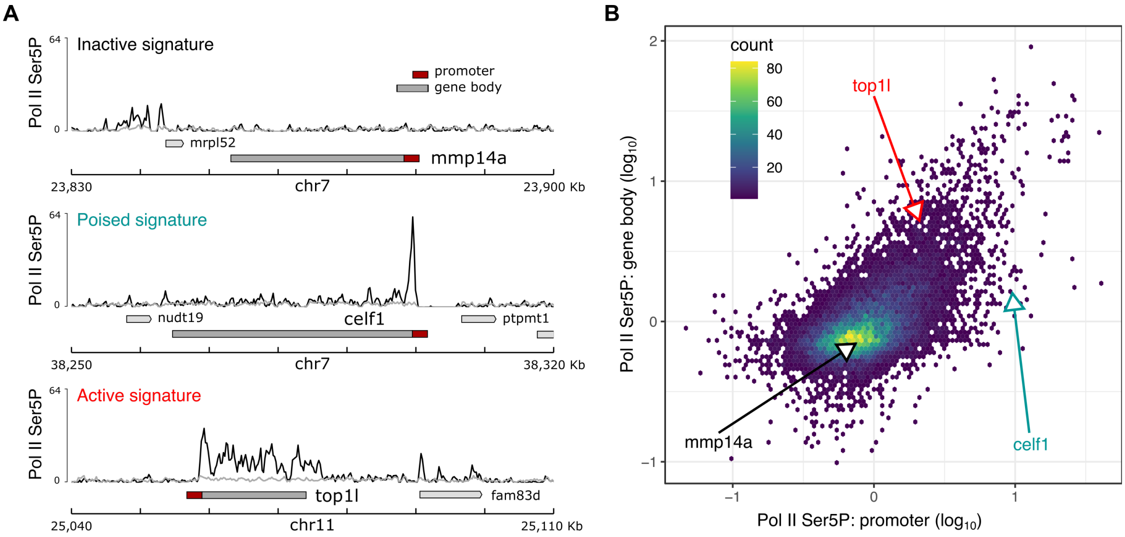

Fig. S7: **Genes with high levels of recruited RNA polymerase II include poised and active genes.** A) Representative coverage tracks of Pol II Ser5P on the promoter (red, 2,000 kb upstream and 200 bases downstream of the TSS) and gene body (dark grey, 200 bases downstream to the TTS) displaying three types of signatures, obtained from aligning Pol II Ser5P ChIP-seq reads from the dome stage on the zebrafish genome. The gene *mmp14a* displays an inactive signature - no signal on either the promoter or on the gene body. The gene *celf1* displays a poised signature - strong Pol II Ser5P signal on the promoter, but not on the gene body. The gene *top1l* displays an active signature - strong Pol II Ser5P signal on both the gene body and the promoter. B) Heat map depicting the counts of genes with different strengths of Pol II Ser5P signal on the promoter and on the gene body for all annotated zebrafish genes. The three genes from panel A are marked. Inactive genes like *mmp14a* have a low Pol II Ser5P signal both on the promoter and the gene body. Such genes make up the bulk of all genes. Poised genes like *celf1* have a strong Pol II Ser5P signal on the promoter but not on the gene body. Active genes such as *top1l* have a strong Pol II Ser5P signal both on the promoter and on the gene body.

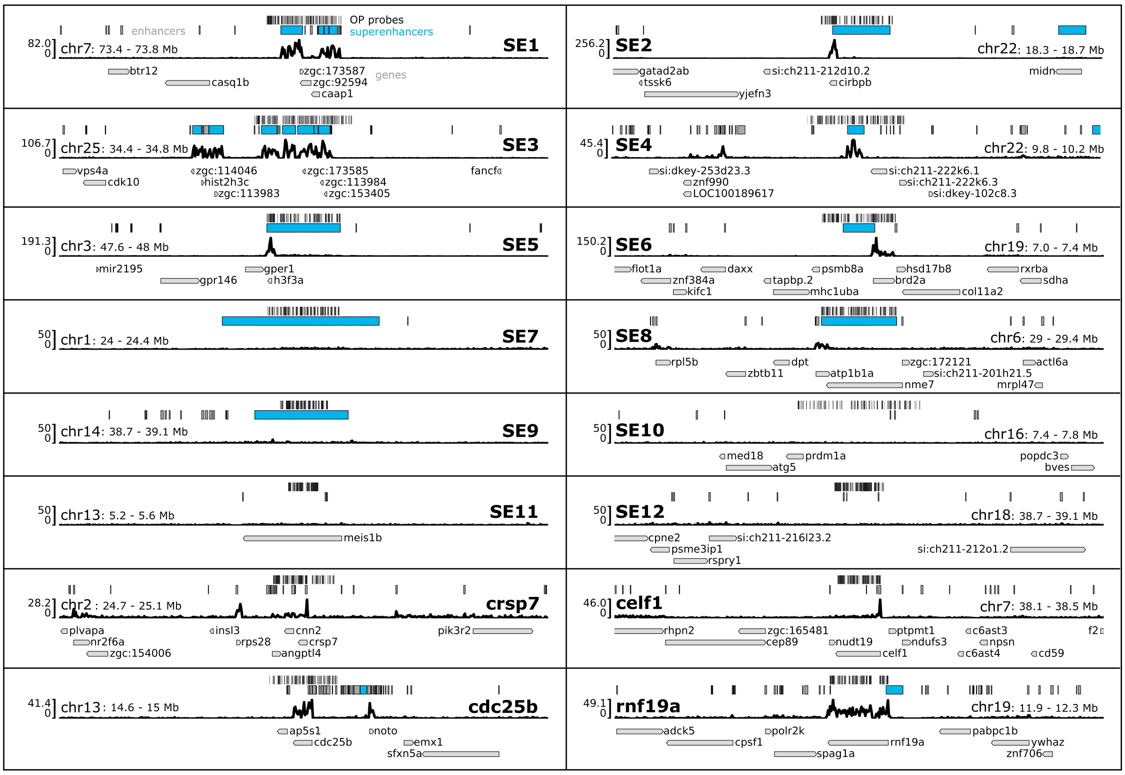

Fig. S8: **Overview of genomic regions labeled by oligopaint probe sets.** Shown are the oligopaint probe sets, identified super-enhancers and enhancers at the dome stage (based on H3K27ac signal), Pol II Ser5P ChIP-seq signal strength, and the genomic context in the 400 kb region around the oligopaint regions. SE1-6 are super-enhancers at the dome stage with a strong Pol II Ser5P signal overlapping the super-enhancer, SE7-9 are super-enhancers at the dome stage without a strong Pol II Ser5P signal, SE10-12 are super-enhancers at the 80% epiboly stage but not at the dome stage, and *crsp7*, *celf1*, *cdc25b*, and *rnf19a* are four genes with a strong H3K27ac signal in the gene body or the promoter regions. The genes *crsp7* and *celf1* have Pol II Ser5P signal only in the promoter, and the genes *cdc25b* and *rnf19a* have Pol II Ser5P signal on the gene body.

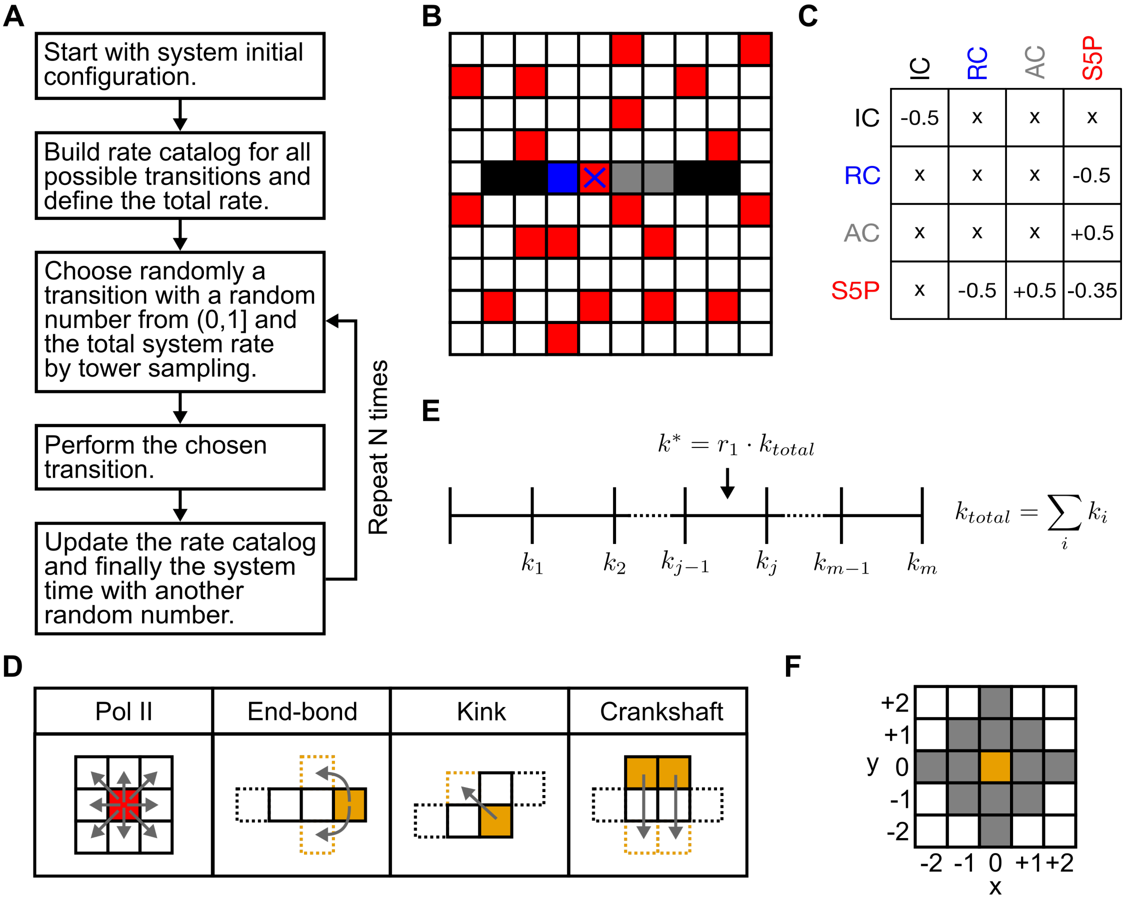

Fig. S9: **Lattice Kinetic Monte Carlo (LKMC) model summary.** A) Algorithm overview. B) Illustration of a possible initial configuration containing a single polymer and all species within the system (black, blue, gray and red). C) Overview table of the interspecies affinity parameters. Table entries represent w values, crosses correspond to no interaction ($w = 0$). D) Possible move types for the different species. Pol II can move to all its eight nearest neighbours. Chains representing chromatin can undergo the Verdier-Stockmayer move set. E) Tower-sampling method to choose a transition. F) Minimal area for the local update of possible transitions.

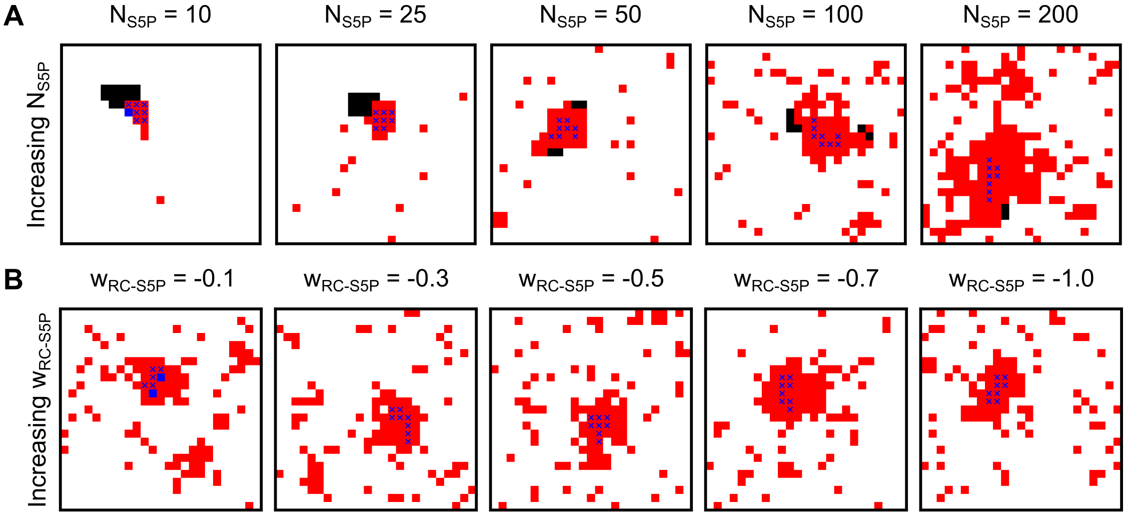

Fig. S10: **Parameter adjustment.** A) Adjustment of N_{S5P}. A single polymer chain of length $L_{Polymer} = 20$, containing eight blue particles was placed in the simulation. $w_{S5P-S5P} = -0.35$ and $w_{RC-S5P} = -0.5$ were held constant. A range of $N_{S5P} = 10, 25, 50, 100, 200$ was used in different simulations as indicated. B) Adjustment of w_{RC-S5P}. A single polymer chain of length $L_{Polymer} = 8$ with only blue particles was placed in the simulation. $w_{S5P-S5P} = -0.35$ and $N_{S5P} = 100$ were held at constant values. A range of $w_{RC-S5P} = -0.1, -0.3, -0.5, -0.7, -1.0$ was used in different simulations as indicated.

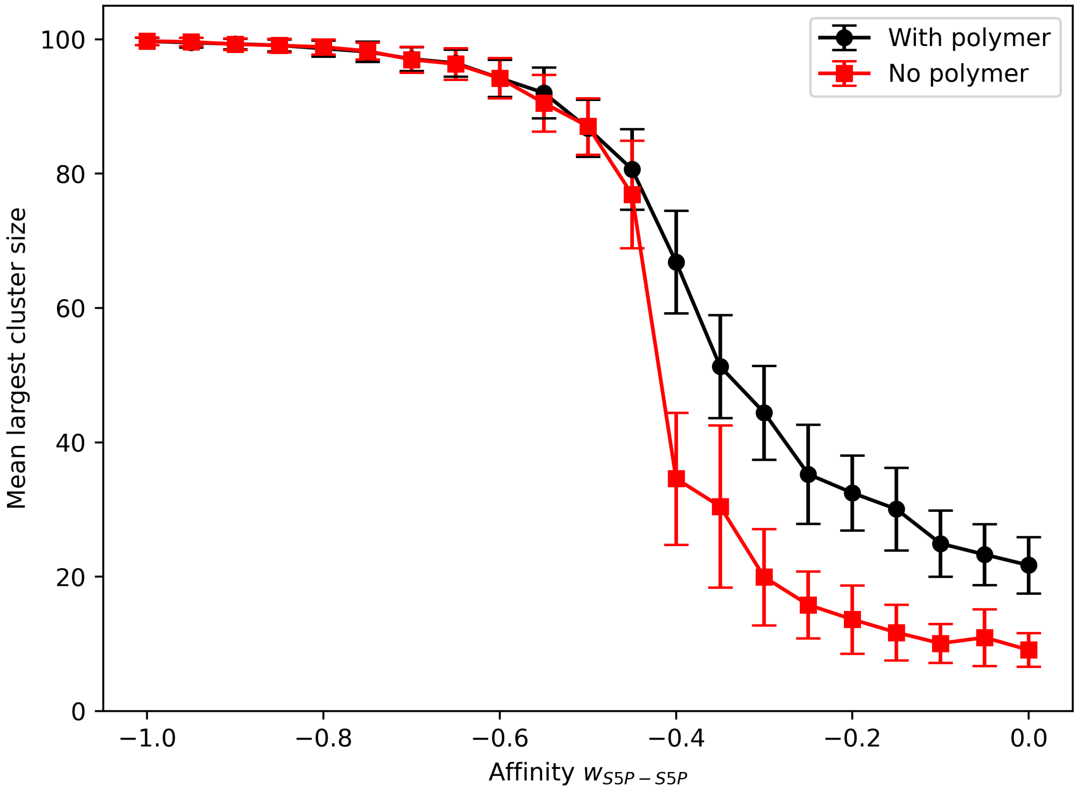

Fig. S11: **Dependence of cluster formation on particle-particle interactions and the presence of a polymer as a condensation surface.** Mean largest cluster size (shown as number of particles in the largest contiguous cluster) of simulations with varying affinity $w_{S5P-S5P} \in [-1.0, 0.0]$. Total number of red particles is constant ($N_{S5P} = 100$) and 200 snapshots were obtained from the time series of a given simulation to calculate the mean of each affinity parameter. Error bars represent standard deviation. Simulations were performed for the case with no polymer (red, only S5P particles) and with a single polymer (black, $L_{polymer} = 20$, $N_{RC} = 8$ and $N_{IC} = 12$).

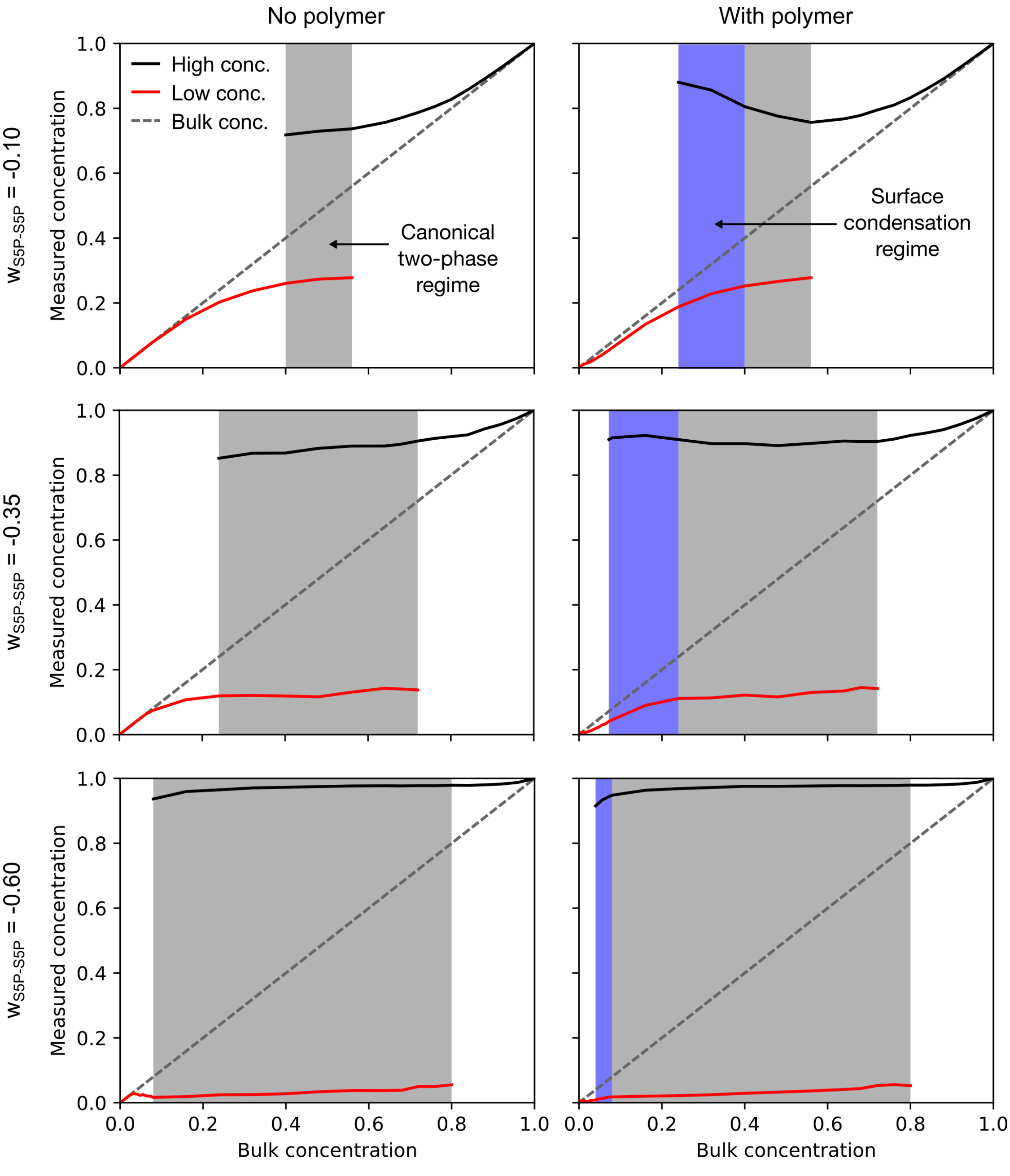

Fig. S12: **Presence of low and high concentration phases in lattice model simulations with and without polymer surface.** Lattice (25×25) simulations of S5P ($N_{S5P} \in [0{,}625]$) without and with polymer ($L_{polymer} = 20$, $N_{RC} = 8$, $N_{IC} = 12$), $w_{RC-S5P} = -0.5$, and $w_{S5P-S5P} = -0.1, -0.35, -0.6$ as indicated). Simulation results are processed by applying a Gaussian filter, phase masks are obtained by thresholding (cut-off 0.5) and subsequent erosion of masks with a square structural element of 3×3 pixels. The bulk concentration is indicated for reference (calculated by dividing N_{S5P} by number of total lattice sites). Shaded areas indicate the canonical two-phase regime (gray, obtained empirically from simulations without polymer) and the surface condensation regime (blue, obtained empirically from simulations with polymer).

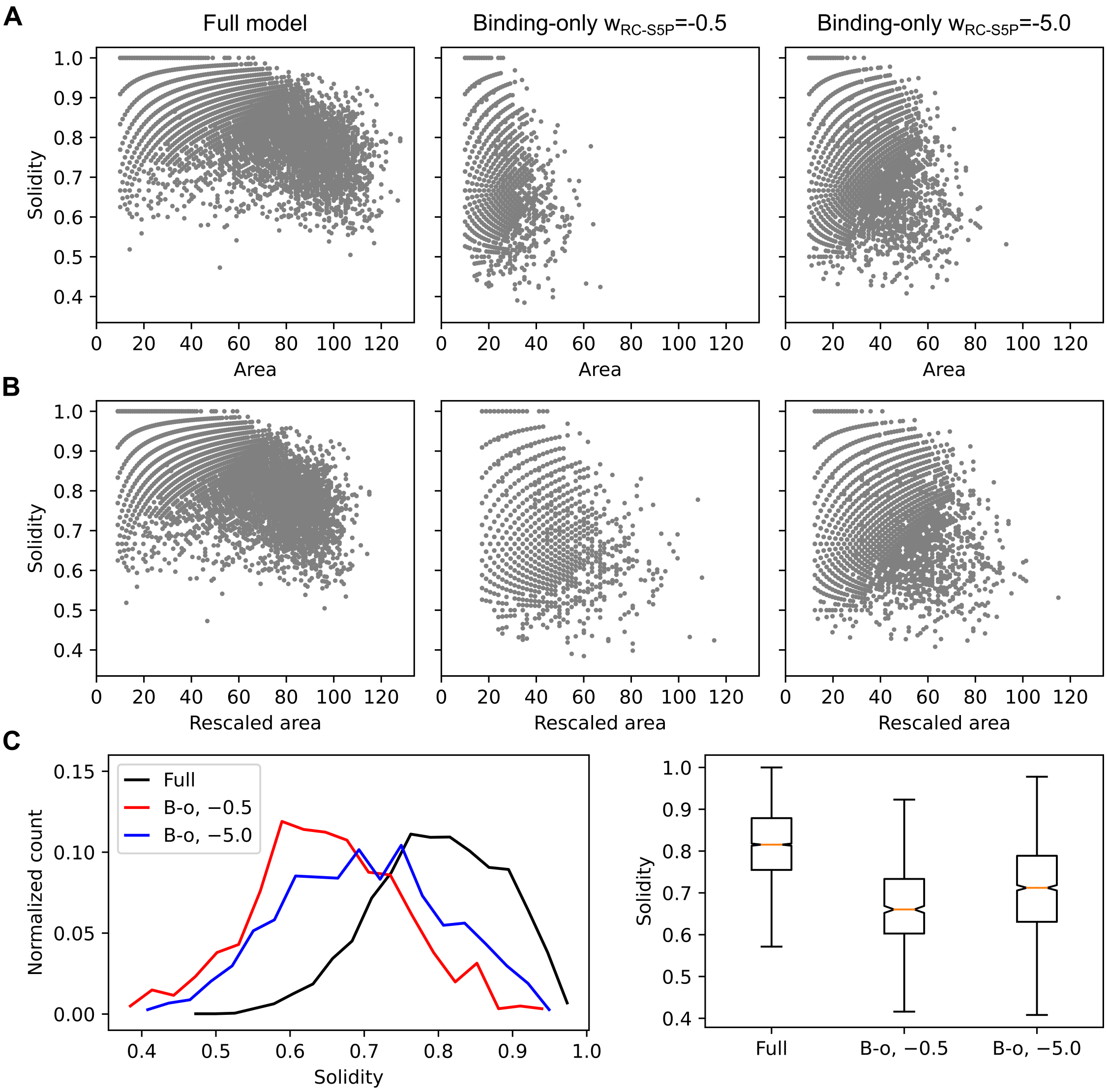

Fig. S13: **Comparison of the full theoretical model and the binding-only model.** A) Scatter plot of cluster solidity vs. cluster area for the full model (original parameters $w_{RC-S5P} = -0.5$ and $w_{S5P-S5P} = -0.35$) and the binding-only model ($w_{S5P-S5P} = 0$ for both cases and $w_{RC-S5P} = -0.5$ or -5.0 as indicated). B) Rescaled scatter plots for the data shown in panel A. Cluster area was linearly rescaled so that the mean value of the top 1% percent cluster area was the same as in the full model simulation. Solidity was not rescaled. C) The solidity of large clusters (rescaled area > 50) visualized as a histogram (plotted as line chart, left) and a boxplot (right).

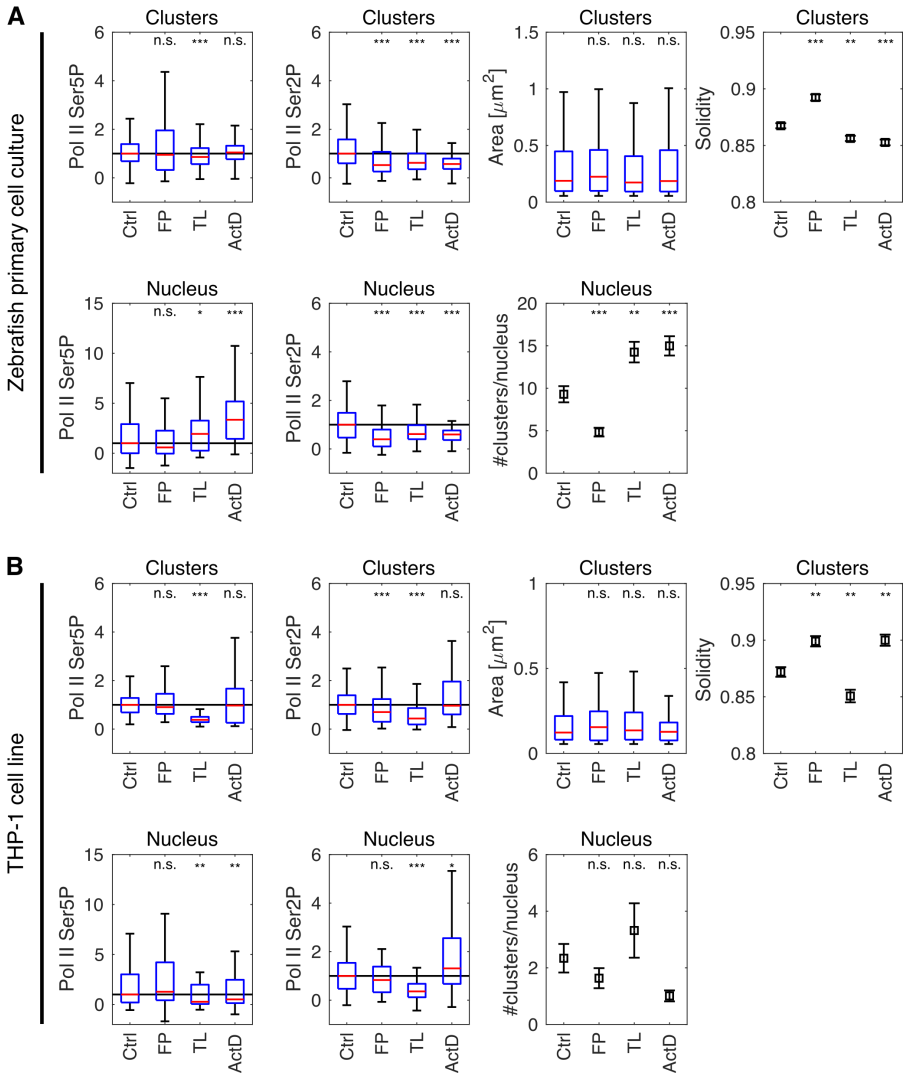

Fig. S14: **Quantification of inhibitor effects.** A) Statistical assessment of changes in primary cell cultures established from oblong stage zebrafish embryos, treated with the transcription inhibitors flavopiridol (FL, 1 μM), triptolide (TL, 500 nM), and actinomycin D (ActD, 5 μg/ml) for 30 min, fixed and labeled by immunofluorescence against RNA polymerase II Ser5P and Pol II Ser2P. Assessed quantities were mean Pol II Ser5P and Pol II Ser2P intensities at Pol II Ser5P clusters, area of clusters, mean Pol II Ser5P and Pol II Ser2P intensities throughout the nucleus (all shown as standard boxplots without outliers), solidity of clusters, and number of clusters per nucleus (mean±SEM, used instead of boxplots and medians to overcome effects of quantized values). Intensity values were normalized by the median intensity of the control samples of a given experimental repeat, as indicated by lines at intensity level 1. *** indicates $P < 0.0003$, ** indicates $P < 0.003$, * indicates $P < 0.017$, n.s. indicates $P \geq 0.017$, significance levels Bonferroni-corrected for multiple testing (Pol II Ser5P, clusters: $P = 0.08$, $P < 0.0001$, $P = 0.03$; Pol II Ser2P, clusters: $P < 0.0001$, $P < 0.0001$, $P < 0.0001$; Area of clusters: $P = 0.02$, $P = 0.08$, $P = 0.92$; Solidity of clusters: $P < 0.0001$, $P = 0.0007$, $P < 0.0001$; Pol II Ser5P, nucleus: $P = 0.17$, $P = 0.014$, $P < 0.0001$; Pol II Ser2P, nucleus: $P < 0.0001$, $P = 0.0001$, $P < 0.0001$; Number of clusters: $P < 0.0001$, $P = 0.001$, $P = 0.0002$; two-tailed permutation test for differences from the control condition, $n = 1534$, 716, 1682, 1604 clusters for intensity calculation, $n = 1514$, 703, 1631, 1567 clusters for solidity calculation, and $n = 165$, 148, 118, 107 nuclei per condition from three independent experimental repeats). B) Corresponding properties of clusters in undifferentiated THP-1 cells treated exactly like zebrafish primary cell culture. (Pol II Ser5P, clusters: $P = 0.12$, $P < 0.0001$, $P = 0.63$; Pol II Ser2P, clusters: $P < 0.0001$, $P < 0.0001$, $P = 0.67$; Area of clusters: $P = 0.03$, $P = 0.38$, $P = 0.92$; Solidity of clusters: $P = 0.002$, $P = 0.00033$, $P = 0.001$; Pol II Ser5P, nucleus: $P = 0.21$, $P = 0.001$, $P = 0.0028$; Pol II Ser2P, nucleus: $P = 0.10$, $P < 0.0001$, $P = 0.004$; Number of clusters: $P = 0.36$, $P = 0.33$, $P = 0.03$; $n = 414$, 152, 302, 117 clusters for intensity calculation, $n = 402$, 150, 294, 114 clusters for solidity calculation, and $n = 177$, 93, 91, 116 nuclei from three independent experimental repeats.)

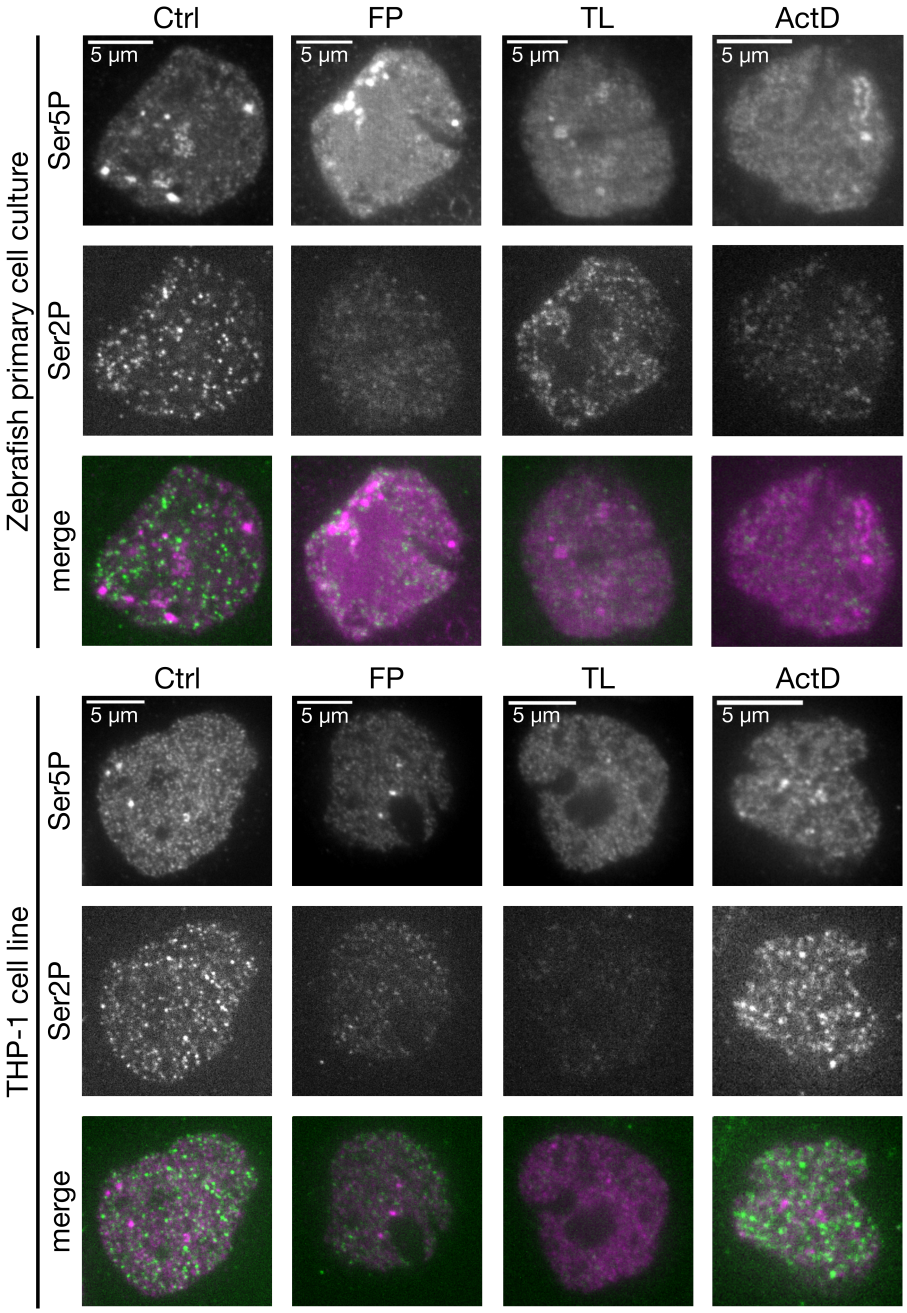

Fig. S15: **Example images of clusters after inhibitor treatment.** Representative mid-nuclear section nuclei in zebrafish primary cell culture (upper three rows) and THP-1 cell culture (lower three rows). Cells were treated with control media (Ctrl), flavopidirol (FP), triptolide (TL) or actinomycin D (ActD) for 30 min, and subsequently stained for Ser5 and Ser2 phosphorylation of RNA polymerase II by indirect immunofluorescence.

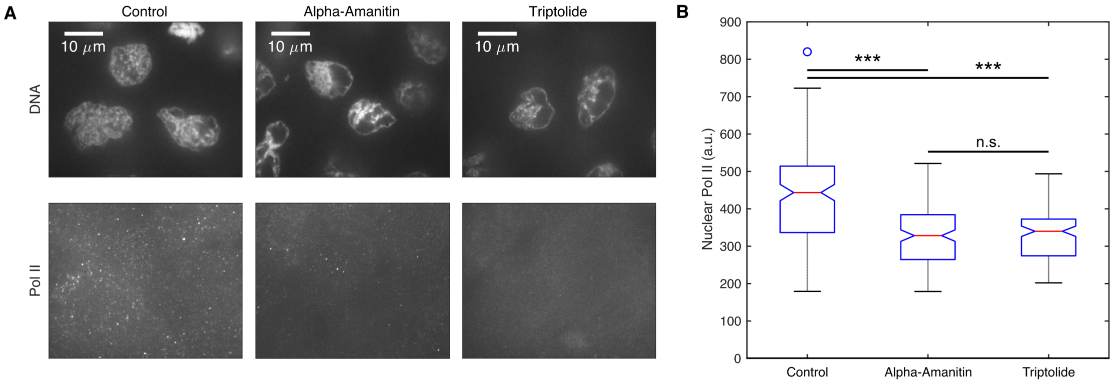

Fig. S16: **Triptolide treatment induces degradation of RNA polymerase II.** A) Representative images of primary cell cultures labeled by immunofluorescence against RNA polymerase II (non phospho-specific anti-Pol II mouse monoclonal antibody 8WG16). Overall loss of Pol II upon alpha-amanitin treatment as well as triptolide treatment can be seen. Primary cell cultures, alpha-amanitin injected at single cell stages, triptolide added at time of culture preparation, culturing time until fixation was 30 min. Single z-sections, DNA labeled with Hoechst 33342. B) Nuclear intensity of Pol II signal, *** indicates P<0.0003, n.s. indicates $P \geq 0.017$ (two-tailed permutation test, significance levels Bonferroni-corrected for multiple testing), $P < 0.0001$,$P < 0.0001$,$P = 0.42$, $n = 164$, 150, 124 nuclei from $N = 3$ independent samples per condition.
