# Supplementary material for: RNA polymerase II clusters form in line with surface condensation on regulatory chromatin
Source: Mol Syst Biol. 2021 Sep 27;17(9):e10272. doi: 10.15252/msb.202110272 (PMC8474054; doi:10.15252/msb.202110272)
Supplement: Supplementary file 2 [file MSB-17-e10272-s001.pdf]

RNA polymerase II clusters form in line with surface condensation on regulatory chromatin

Agnieszka Pancholi, Tim Klingberg, Weichun Zhang, Roshan Prizak, Irina Mamontova, Amra Noa, Marcel Sobucki, Andrei Kobitski, G. Ulrich Nienhaus, Vasily Zaburdaev, and Lennart Hilbert
DOI: 10.15252/msb.202110272

Corresponding author(s): Lennart Hilbert (lennart.hilbert@kit.edu) , G. Ulrich Nienhaus (uli@uiuc.edu), Vasily Zaburdaev (vasily.zaburdaev@fau.de)

Review Timeline:

Submission Date:	3rd Feb 21
Editorial Decision:	23rd Mar 21
Revision Received:	24th Jun 21
Editorial Decision:	2nd Aug 21
Revision Received:	26th Aug 21
Accepted:	10th Sep 21

Editor: Maria Polychronidou

Transaction Report:

(Note: With the exception of the correction of typographical or spelling errors that could be a source of ambiguity, letters and reports are not edited. Depending on transfer agreements, referee reports obtained elsewhere may or may not be included in this compilation. Referee reports are anonymous unless the Referee chooses to sign their reports.)

1st Editorial Decision 23rd Mar 2021

Thank you again for submitting your work to Molecular Systems Biology. I would like to apologise for the delay in sending you a decision on your work. Unfortunately, despite several reminders we have not managed to obtain a report from reviewer #3. In the interest of time, we have now decided to proceed with making a decision based on the two available reports. As you will see below, the two reviewers acknowledge that the presented findings seem relevant and timely. They raise however a series of concerns, which we would ask you to address in a major revision.

I think that the recommendations of the reviewers are rather clear. Therefore, I do not see the need to repeat the points listed below. All issues raised by the reviewers need to be satisfactorily addressed. Please contact me in case you would like to discuss in further detail any of the issues raised.

On a more editorial level, we would ask you to address the following points.

REFEREE REPORTS

Reviewer #1:

Pancholi and Klingberg et al. use live-cell imaging of Pol II labelled with microinjected phosphoCTD-specific Fab fragments against Ser5P and Ser2P and computational modeling to analyze the distribution of Pol II in the nucleus of early-stage zebrafish embryos. They find that the distribution of the Ser5P and Ser2P forms of Pol II is not uniform - but clustered, similar to what others have found previously in human cell lines using immunofluorescence microscopy. Their analysis shows that both modification types appear in separate but adjacent clusters and identifies three distinct morphology types based on the respective levels for Ser5P and Ser2P Pol II. Using computational coarse-grained modeling and Monte Carlo simulations, the authors build a computational model of Ser5P-Pol II LLPS to propose the hypothesis that chromatin could act as a scaffold to facilitate Pol II

clustering even at subcritical concentrations - a process that others have previously termed 'wetting'. The authors then use their model to predict synthetic microscopy images, which they find resemble the cluster types detected in cells and to predict the effect of the two transcription inhibitors triptolide and flavopridol.

Given the fields enthusiasm regarding the role of phase separation during transcription regulation, the manuscript addresses an important and interesting topic in a timely manner. The approach to combine the experimental interrogation of Pol II distribution with computational modeling is certainly attractive for a cross-disciplinary readership of a journal like Molecular Systems Biology. Overall, the manuscript is well written and the authors do a good job in explaining their approach in a way also understandable to readers from foreign fields. In some points, however, conclusions are reached without exploring alternative models or validating them thoroughly in the experimental set-up. In particular, for their computational analysis the authors assume that all detected Ser5P and Ser2P Pol II clusters are formed by phase separation without exploring alternatives. While it has been shown in vivo (as the authors mention) and biochemically that Ser5P and Ser2P forms of CTD do not self-interact to undergo phase separation in isolation and require additional proteins to form (heterotypic) condensates, the authors neglect this in their simplified model and focus exclusively on Pol II. The authors thus need to make an additional effort - under consideration of the following points - before the manuscript is considered for publication in Molecular Systems Biology.

1. The authors refer to the forms of Pol II detected with the Ser5P antibody as recruited Pol II, while they refer to the forms of Pol II detected with the Ser2P antibody as elongating Pol II. Although enabling many breakthrough discoveries, the characterization of CTD phosphorylation by using 'phosphorylation mark-specific' antibodies is facing a somewhat ambivalent history due to unneglectable factors such as pronounced cross-activity or shielding of epitopes by CTD binding proteins that has affected some interpretations within the transcription field. Unfortunately, the authors do not describe in the appropriate part of the methods section, which antibodies they used for the analysis (Hybridoma clone, Supplier, Lot number etc.). To justify referring to the detected forms as recruited and elongating Pol II, the authors should ideally establish that Ser5P Pol II clusters indeed co-localize with the promoter or 5'-region of transcription units, while at the same time Ser2P Pol II clusters do not. This could be done for example by using fluorescence in situ hybridization. Such an experiment would also help to clarify another aspect, namely to establish that the detected Pol II clusters are actually chromatin-associated and this way provide more evidence for a role of chromatin in Pol II cluster assembly.
2. The authors observe that Ser5P and Ser2P Pol II form discrete clusters with virtually no overlap. Extensive ChIP studies with phosphospecific CTD antibodies have shown that elongating Pol II is modified on both Ser5P and Ser2P over a substantial part of the gene body in various organisms (from yeast to human). In this context, how do the authors explain the almost switch-like transition they observe in their data? To which cluster does Pol II translocate that is phosphorylated concomitantly on Ser5P and Ser2P?
3. The extended Zebrafish CTD with its 52x repeats has a length of roughly about 150 nm and can theoretically bind multiple (maybe up to a dozen) antibody molecules simultaneously. While it appears likely that even the very small (maybe ~100 nm, e.g. Fig. 2B) type I-clusters are composed of multiple Pol II molecules, it seems that the authors cannot really exclude that the signal they detect stems from just a single Pol II molecule which binds multiple Fab fragments simultaneously. The fluorescence intensity of the cluster is thus not necessarily proportional to the number of Pol II molecules. The authors should clarify this limitation of their methodology in the manuscript text or provide experimental data that disproves it.
4. The authors should establish that Ser5P and Ser2P Pol II type i-iii clusters which they use to build their computational LLPS model actually recapitulate properties of bona fide condensates, and are

not merely result from multiple closely spaced Pol II molecules transcribing along a gene upon a transcriptional burst. Did the authors observe fusion events between different Pol II clusters in live-cell microscopy or explore the effect of 1,6-hexanediol on the Pol II condensates?

5. "The effect on phosphorylation levels of Pol II clusters was in line with expectations: triptolide reduced the Pol II Ser5P and Pol II Ser2P intensities, while flavopiridol reduced Pol II Ser2P levels (Fig. 5)." Within the referred Fig. 5, the authors show the exact opposite result of what they describe in the main text. Fig. 5A shows that treatment with triptolide (TL) reduced Ser2P levels while not affecting Ser5P levels and that treatment with flavopiridol (FP) reduced Ser2P as well as Ser5P levels. The authors need to resolve this.

6. In addition to the rapid inhibition of XBP, prolonged exposure to triptolide (>1 h) also triggers proteasomal degradation of Pol II. Did the authors verify that the loss of CTD phosphorylation signal actually quantitatively results from inhibition and not from degradation of Pol II at such comparably high concentrations used in the experiment? In this regard, the authors unfortunately do not provide any information in the methods section about the length of the inhibitor treatment prior to imaging. To establish that the effect of triptolide on CTD phosphorylation levels is not caused by Pol II degradation, the authors can compare Pol II levels of inhibitor and mock treated cells. This is important as the authors build their computational model on these results and could be simply verified by Western blotting using CTD phosphorylation-independent Pol II antibodies.

7. In their model figure in Fig. 5B, the authors show that the droplet containing Ser5P forms upstream of the transcription start site (TSS). In contrast, a plethora of previous biochemical studies have established that incorporation of Pol II into the pre-initiation complex at the promoter requires an unphosphorylated CTD and only upon transcription initiation the CTD becomes phosphorylated on Ser5 through the CDK7 kinase which is part of TFIIH. The schematic model is thus somewhat misleading. The Ser5P droplet would need to be positioned within the gene body between the TSS and the pause site (a genetic region of ~50-100 bp, which assuming a Pol II footprint of ~35 bps, equals a space for maybe about 2-3 initiated Pol II molecules). Please clarify.

8. Please fix the arrangement/order of panels showing images of Ser5P and Ser2P. For example in Fig. 2A, Ser5P is the first microscopy image followed by Ser2P, while in Fig. 2B the authors start with Ser2P followed by Ser5P. This hampers readability and is confusing for the reader. As during the transcription cycle, Ser5P signal precedes and is mechanistically required for Ser2P, I would thus suggest arranging all the panels in this logical order.

9. The first subpart of the results section concludes with the statement that "recruited Pol II forms [...] clusters with a rich array of morphologies that appear to vary with the levels of Pol II Ser2 and Ser5 phosphorylation [...]". The following subpart of the results section starts with the redundant caption: "Levels of recruited and elongating Pol II correlate with cluster morphology". The authors should rephrase wording of the different sections according to what unique information they provide or combine them into a single section.

10. In Fig. 2G, both the x- and y-axis are labelled with "Recruited Pol II". The authors need to correct this mistake.

11. Although the experiments using transcription inhibitors cover 2 of 6 (1/3) main figures and about a similar amount of the manuscript text, the authors do not mention this at all in the abstract. The abstract should present essential parts and clearly communicate the major results.

12. Caption, Fig. 5: "Characterization of kinase inhibitor effects". Triptolide is not a kinase inhibitor, it inhibits the ATPase activity of XPB, the helicase/translocase subunit of TFIIH and thus dsDNA unwinding within the initiation complex. The authors need to correct this.

Reviewer #2:

The manuscript by Pancholi and Klinberg et al analyze Pol II recruitment in exhibited morphologies

of clusters using live-cell and super-resolution microscopy in zebrafish embryos along with lattice simulations of condensation. For simulations, they studied liquid phase condensation on block copolymers in place of chromatin. Using structured illumination microscopy (SIM) and stimulated emission double depletion microscopy (STEDD) helped them to observe Pol II association with large clusters, and elongating Pol II with dispersed clusters, as previously has been shown. Post-transcriptional modifications (including phosphorylation of serine 5 and serine 2) that control the interaction of Pol II with clusters were used to further understand the morphologies of clusters at different stages. They claim their findings support previous claims about wetting of chromatin that might be similar to wetting behavior of liquid phase. Physicochemical understanding of wetting behavior of phase separated clusters could enable us to have a better understanding and control over the systems. Therefore, it is valuable to begin to elucidate the impact of physical characteristic of phase separated clusters in vivo, which is usually ignored in studies that more often focus on formation of clusters. This manuscript makes a nice start towards understanding how morphologies of phase separated systems are related on molecular level changes. I recommend publication after the following comments have been addressed:

Major:

1. There are significant work on wetting behavior and what could shape of phase separated droplets could tell us about the physical state they are in. Could you give us an explanation/interpretation what is different about type i, ii and iii? Why do you observe these different shapes? What type of changes could explain these differences?

2. Why is Pol II Ser2P levels are different than Pol II Ser5P level?

3. Could you explain what is the difference in lattice simulation output results of type i, ii and iii? What does it imply? Could you speculate?

Minor:

4. SI Figure 8 and 10 are never mentioned in the manuscript. If they are not significant and contributing the story, take them out. If not, please explain.

Reviewer #1:

Pancholi and Klingberg et al. use live-cell imaging of Pol II labelled with microinjected phosphoCTD-specific Fab fragments against Ser5P and Ser2P and computational modeling to analyze the distribution of Pol II in the nucleus of early-stage zebrafish embryos. They find that the distribution of the Ser5P and Ser2P forms of Pol II is not uniform - but clustered, similar to what others have found previously in human cell lines using immunofluorescence microscopy. Their analysis shows that both modification types appear in separate but adjacent clusters and identifies three distinct morphology types based on the respective levels for Ser5P and Ser2P Pol II. Using computational coarse-grained modeling and Monte Carlo simulations, the authors build a computational model of Ser5P-Pol II LLPS to propose the hypothesis that chromatin could act as a scaffold to facilitate Pol II clustering even at subcritical concentrations - a process that others have previously termed 'wetting'. The authors then use their model to predict synthetic microscopy images, which they find resemble the cluster types detected in cells and to predict the effect of the two transcription inhibitors triptolide and flavopridol.

Given the fields enthusiasm regarding the role of phase separation during transcription regulation, the manuscript addresses an important and interesting topic in a timely manner. The approach to combine the experimental interrogation of Pol II distribution with computational modeling is certainly attractive for a cross-disciplinary readership of a journal like Molecular Systems Biology. Overall, the manuscript is well written and the authors do a good job in explaining their approach in a way also understandable to readers from foreign fields. In some points, however, conclusions are reached without exploring alternative models or validating them thoroughly in the experimental set-up. In particular, for their computational analysis the authors assume that all detected Ser5P and Ser2P Pol II clusters are formed by phase separation without exploring alternatives. While it has been shown in vivo (as the authors mention) and biochemically that Ser5P and Ser2P forms of CTD do not self-interact to undergo phase separation in isolation and require additional proteins to form (heterotypic) condensates, the authors neglect this in their simplified model and focus exclusively on Pol II. The authors thus need to make an additional effort - under consideration of the following points - before the manuscript is considered for publication in Molecular Systems Biology.

We thank the referee for their thorough and knowledgeable report. We have addressed all concerns, leading to a more comprehensive perspective on the roles of Pol II Ser5P and Pol II Ser2P in the cluster formation process.

In direct response to the referee's summary, we want to state that we are aware that the Pol II Ser5P mark is primarily a marker, not a constituent, of condensates that contain numerous other molecular components. We have indicated this more prominently in the graphical synopsis and the illustration in Fig. 1, by a shaded region and the text labels in the figures. We also mention this point when we construct the model, referring to a liquid phase material enriched in recruited Pol II (lines 347-349):
"[...] we introduced a ``red'' particle species that represents the material forming the clusters enriched in recruited Pol II (Fig. 5A)."

1. The authors refer to the forms of Pol II detected with the Ser5P antibody as recruited Pol II, while they refer to the forms of Pol II detected with the Ser2P antibody as elongating Pol II. Although enabling many breakthrough discoveries, the characterization of CTD phosphorylation by using 'phosphorylation mark-specific' antibodies is facing a somewhat ambivalent history due to unneglectable factors such as pronounced cross-activity or shielding of epitopes by CTD binding proteins that has affected some interpretations within the transcription field. Unfortunately, the authors do not describe in the appropriate part of the methods section, which antibodies they used

for the analysis (Hybridoma clone, Supplier, Lot number etc.). To justify referring to the detected forms as recruited and elongating Pol II, the authors should ideally establish that Ser5P Pol II clusters indeed co-localize with the promoter or 5'-region of transcription units, while at the same time Ser2P Pol II clusters do not. This could be done for example by using fluorescence in situ hybridization. Such an experiment would also help to clarify another aspect, namely to establish that the detected Pol II clusters are actually chromatin-associated and this way provide more evidence for a role of chromatin in Pol II cluster assembly.

Antibodies: *We added clone number, catalog number, and for primary antibodies also lot numbers (new Tables 1 & 2, page 28). The antibody fragments (used only for live imaging) are produced by the Kimura lab and not commercially available. Please note that the antibodies for fixed imaging and the antibody fragments used for live imaging are different clones, so that these are already two different antibody sets, whose results are in agreement. We now added experiments with one more antibody against Pol II Ser5P, providing a third antibody to visualize cluster morphologies. This third antibody reproduced the relation of cluster morphology to Pol II phosphorylation seen with the other two antibodies against Pol II Ser5P (added to Fig. EV3). These additional experiments should largely rule out antibody-specific effects.*

Colocalization of Pol II Ser5P and Pol II Ser2P signal (new Figs. EV4 and EV5): *As suggested by the referee, we could also confirm the presence of Pol II Ser5P in the gene bodies of transcribed genes in a reanalysis of previously published ChIP-Seq data (new Fig. EV5). We therefore needed to understand how this can be reconciled with the apparent exclusion of Pol II Ser2P from clusters with high Pol II Ser5P signal. To assess both phospho-marks with improved imaging resolution (STEDD as contained in the original submission only super-resolved the Pol II Ser5P channel), we carried out two-color STED microscopy (new Fig. EV4). We find that mutual exclusion between Pol II Ser5P and high levels of Pol II Ser2P indeed occurs, but only for large clusters of Pol II Ser5P. Among the small clusters of Pol II Ser5P, there are some that display colocalization of high Pol II Ser5P and Pol II Ser2P levels, exactly as would be expected from our ChIP-seq analysis. The distinction of mutual exclusion for large clusters, and the possibility of colocalization in small clusters makes it a lot clearer what the cluster types I, II, and iii represent, so we are happy the referee asked us about this point.*

Clarification of underlying chromatin regions (ChIP-seq reanalysis, 3-color STED, and DNA-FISH, new Fig. 4): *Here, we followed up on the question about the chromatin regions underlying the Pol II Ser5P clusters, and also the suggestion to use DNA-FISH. Again, we first reanalyzed previously published ChIP-seq data, finding that some super-enhancer regions and genes with high levels of H3K27ac were highly enriched for Pol II Ser5P binding. To assess the relation between Pol II Ser5P, H3K27ac, and overall chromatin organization, we optimized our STED microscopy approach to achieve 3-color imaging. These new microscopy experiments revealed (i) that the overall chromatin density is reduced inside large Pol II Ser5P clusters, (ii) that H3K27ac is enriched inside large Pol II clusters despite the exclusion of bulk chromatin, indicating a selective localization of H3K27ac, and (iii) that Pol II Ser5P clusters that contain higher levels of H3K27ac are larger and more compact. Lastly, we implemented the oligopaint DNA-FISH approach (first application of that method in zebrafish, as far as we know) to sequence-specifically label representative super-enhancer regions and genes. Here, we find that super-enhancers with high Pol II Ser5P levels in ChIP-seq data, and some genes indeed associate with Pol II Ser5P clusters. The new data mentioned under this point are all presented in the newly added Fig. 4.*

Please note that the sequence resolution provided by the combination of our microscopy techniques with the DNA-FISH (required labeled regions: >30 kilobases) is insufficient to carry out the suggested experiment (5' vs gene body FISH combined with Pol II Ser5P and Pol II Ser2P

immunofluorescence). We hope, however, that the other experiments added under this point and below, in effect, remedy the concerns of specificity of the Pol II Ser5P and Ser2P antibodies.

2. The authors observe that Ser5P and Ser2P Pol II form discrete clusters with virtually no overlap. Extensive ChIP studies with phosphospecific CTD antibodies have shown that elongating Pol II is modified on both Ser5P and Ser2P over a substantial part of the gene body in various organisms (from yeast to human). In this context, how do the authors explain the almost switch-like transition they observe in their data? To which cluster does Pol II translocate that is phosphorylated concomitantly on Ser5P and Ser2P?

We revised our previous conclusion of a complete exclusion between Pol II Ser5P and Pol II Ser2P, and instead now conclude that transcribed genes are excluded from large clusters (type ii and iii), and can show up as type i clusters co-stained for Pol II Ser5P and Pol II Ser2P. This revised conclusion is in line with the referee's statement, and supported by our added reanalysis of ChIP-seq data and two-color STED microscopy (new Figs. EV4 and EV5), see also above response to point 1.

3. The extended Zebrafish CTD with its 52x repeats has a length of roughly about 150 nm and can theoretically bind multiple (maybe up to a dozen) antibody molecules simultaneously. While it appears likely that even the very small (maybe ~100 nm, e.g. Fig. 2B) type I-clusters are composed of multiple Pol II molecules, it seems that the authors cannot really exclude that the signal they detect stems from just a single Pol II molecule which binds multiple Fab fragments simultaneously. The fluorescence intensity of the cluster is thus not necessarily proportional to the number of Pol II molecules. The authors should clarify this limitation of their methodology in the manuscript text or provide experimental data that disproves it.

We added the following statement to the Results section (lines 151-154):
"Note that the Pol II CTD YSPTSPS array is repeated 52 times per Pol II complex in zebrafish, implying that (i) fluorescence intensity is not necessarily directly proportional to molecule numbers, and (ii) signal is amplified, so that spots might correspond to single genes, or even single polymerases."

Further, based on our new two-color STED microscopy of Pol II Ser5P and Pol II Ser2P (new Fig. EV4) and new analyses of Pol II Ser5P ChIP-seq data (new Fig. EV5), we would agree with the referee that it is quite possible that type i clusters simply represent single genes. We added an according explanation in the revised manuscript (202-219).

4. The authors should establish that Ser5P and Ser2P Pol II type i-iii clusters which they use to build their computational LLPS model actually recapitulate properties of bona fide condensates, and are not merely result from multiple closely spaced Pol II molecules transcribing along a gene upon a transcriptional burst. Did the authors observe fusion events between different Pol II clusters in live-cell microscopy or explore the effect of 1,6-hexanediol on the Pol II condensates?

We have added the new Fig. 3 to assess the possibility of bona fide LLPS - and argue, based on this figure, that bona fide LLPS must be rejected as an explanation of our observations.
First we applied hexanediol treatment that is frequently used to trigger the dissolution of liquid-like condensates. For Pol II Ser2P, we find small spots, which are unaffected by hexanediol treatment - most likely representing convoys of elongating Pol II that are fully engaged with the DNA template, just as described by the referee.For Pol II Ser5P, we find that clusters unfold and split into smaller clusters - instead of fully disappearing, as would be expected for bona fide LLPS.

Second, live imaging (in absence of hexanediol) shows (i) a pattern of small phase domains that remain stable for over ten minutes, as well as (ii) repeated connection-splitting cycles between neighboring segments of clusters. These observations are in strong discrepancy with an LLPS scenario, where small droplets dissolve over time and contribute to growth of larger droplets (Ostwald ripening) while coalescence of droplets is irreversible. Importantly, all these observations were reproduced by additional analyses of our lattice model (new panels in Fig. 5). We would like to point out that no changes needed to be made to our model assumptions or its parameters, the model already reproduced all these phenomena in its original formulation, we just needed to produce additional simulation outputs.

5. "The effect on phosphorylation levels of Pol II clusters was in line with expectations: triptolide reduced the Pol II Ser5P and Pol II Ser2P intensities, while flavopiridol reduced Pol II Ser2P levels (Fig. 5)." Within the referred Fig. 5, the authors show the exact opposite result of what they describe in the main text. Fig. 5A shows that treatment with triptolide (TL) reduced Ser2P levels while not affecting Ser5P levels and that treatment with flavopiridol (FP) reduced Ser2P as well as Ser5P levels. The authors need to resolve this.

We would like to refer to Fig. 7, panel B, which now shows the statistical analysis of the Pol II Ser5P and Ser2P levels in a boxplot display (previously contained only in a supplementary figure). These results are exactly what we describe in the main text, and hopefully this less convoluted type of plot, in combination with a statistical analysis, makes the described changes more evident.

6. In addition to the rapid inhibition of XBP, prolonged exposure to triptolide (>1 h) also triggers proteasomal degradation of Pol II. Did the authors verify that the loss of CTD phosphorylation signal actually quantitatively results from inhibition and not from degradation of Pol II at such comparably high concentrations used in the experiment? In this regard, the authors unfortunately do not provide any information in the methods section about the length of the inhibitor treatment prior to imaging. To establish that the effect of triptolide on CTD phosphorylation levels is not caused by Pol II degradation, the authors can compare Pol II levels of inhibitor and mock treated cells. This is important as the authors build their computational model on these results and could be simply verified by Western blotting using CTD phosphorylation-independent Pol II antibodies.

We usually apply transcription inhibitors such as triptolide for 30 minutes only, to avoid the types of side effects mentioned by the referee (only slow-acting compounds, such as alpha-amanitin, are applied for longer). We now mention this treatment duration also in the main results section (previously contained only in the methods in our original submission).

As suggested, we quantified Pol II degradation with a pan-Pol II antibody, and found that triptolide indeed leads to significant loss of Pol II (new Fig. EV11). At the same time, the overall Pol II Ser5P levels in the nucleus are increased, the Pol II Ser5P level at the clusters is mildly decreased, and Pol II Ser2P is down overall and in clusters (Fig. 7B). We went through the relevant literature (we added the citations Titov et al. 2011, Wang et al. 2011, Manzo et al. 2012) and these effects seem to be exactly what is expected: Pol II degradation, Ser5 hyper-phosphorylation, Pol II Ser5P release from chromatin, and inhibition of pause-release into elongation. We described these observations, and how they connect to the known mechanisms of action of triptolide, in the revised submission (lines 425-441).

7. In their model figure in Fig. 5B, the authors show that the droplet containing Ser5P forms upstream of the transcription start site (TSS). In contrast, a plethora of previous biochemical studies have established that incorporation of Pol II into the pre-initiation complex at the promoter requires an unphosphorylated CTD and only upon transcription initiation the CTD becomes phosphorylated

on Ser5 through the CDK7 kinase which is part of TFIIH. The schematic model is thus somewhat misleading. The Ser5P droplet would need to be positioned within the gene body between the TSS and the pause site (a genetic region of ~50-100 bp, which assuming a Pol II footprint of ~35 bps, equals a space for maybe about 2-3 initiated Pol II molecules). Please clarify.

The referee pointed out a misunderstanding on our end, and we corrected our Introduction and the scheme in Fig. 1 to indicate that Ser5 phosphorylation only occurs during initiation. Pol II Ser5P can then advance towards elongation, or can be prematurely released from chromatin and become part of the condensate. The scheme in Fig. 5 got deleted, as we completely removed that model in response to referee #3.

The placement of the condensate with respect to the gene promoter and gene body is a little more involved. As pointed out above by the referee, the condensate contains numerous other species of molecules, not only Pol II. Accordingly, the positioning of Pol II Ser5P on to the genomic sequence cannot be used directly to infer that placement. Instead, we rely on previous findings, stating that these types of condensates form in association (loose association, not necessarily prolonged binding) with regulatory regions. This aligns well with our findings, where only enhancers and genes without Ser2P can be inside large Ser5P clusters, suggesting a picture where the condensate does not extend far into the gene body. In our updated sketch in Fig. 1, the condensate therefore covers the promoter, and also the region where initiation proceeds that was pointed out by the referee.

8. Please fix the arrangement/order of panels showing images of Ser5P and Ser2P. For example in Fig. 2A, Ser5P is the first microscopy image followed by Ser2P, while in Fig. 2B the authors start with Ser2P followed by Ser5P. This hampers readability and is confusing for the reader. As during the transcription cycle, Ser5P signal precedes and is mechanistically required for Ser2P, I would thus suggest arranging all the panels in this logical order.

We apologize for this inconsistency, and now put Pol II Ser5P first, Pol II Ser2P second everywhere.

9. The first subpart of the results section concludes with the statement that "recruited Pol II forms [...] clusters with a rich array of morphologies that appear to vary with the levels of Pol II Ser2 and Ser5 phosphorylation [...]". The following subpart of the results section starts with the redundant caption: "Levels of recruited and elongating Pol II correlate with cluster morphology". The authors should rephrase wording of the different sections according to what unique information they provide or combine them into a single section.

This also is a good comment, we tried to make clearer what the point of each section is. The section headers are now the following:

"Recruited RNA polymerase II occurs in clusters exhibiting different types of morphologies." (page 5)

"Recruited Pol II is associated with large clusters, while elongating Pol II is located at the margins of unfolded large clusters." (page 7)

10. In Fig. 2G, both the x- and y-axis are labelled with "Recruited Pol II". The authors need to correct this mistake.

Indeed. We corrected this.

11. Although the experiments using transcription inhibitors cover 2 of 6 (1/3) main figures and about a similar amount of the manuscript text, the authors do not mention this at all in the abstract. The abstract should present essential parts and clearly communicate the major results.

The extensive revisions required rewriting of the abstract. The abstract now also mentions the inhibitor experiments.

12. Caption, Fig. 5: "Characterization of kinase inhibitor effects". Triptolide is not a kinase inhibitor, it inhibits the ATPase activity of XPB, the helicase/translocase subunit of TFIIH and thus dsDNA unwinding within the initiation complex. The authors need to correct this.

This was a mistake on our end, which we corrected. As mentioned for point 6, we generally revisited the role of triptolide, largely because of this comment. We are glad the referee mentioned this point, so we could correct our wrong understanding of the mechanism of action of triptolide.

Reviewer #2:

The manuscript by Pancholi and Klinberg et al analyze Pol II recruitment in exhibited morphologies of clusters using live-cell and super-resolution microscopy in zebrafish embryos along with lattice simulations of condensation. For simulations, they studied liquid phase condensation on block copolymers in place of chromatin. Using structured illumination microscopy (SIM) and stimulated emission double depletion microscopy (STEDD) helped them to observe Pol II association with large clusters, and elongating Pol II with dispersed clusters, as previously has been shown. Post-transcriptional modifications (including phosphorylation of serine 5 and serine 2) that control the interaction of Pol II with clusters were used to further understand the morphologies of clusters at different stages. They claim their findings support previous claims about wetting of chromatin that might be similar to wetting behavior of liquid phase. Physicochemical understanding of wetting behavior of phase separated clusters could enable us to have a better understanding and control over the systems. Therefore, it is valuable to begin to elucidate the impact of physical characteristic of phase separated clusters in vivo, which is usually ignored in studies that more often focus on formation of clusters. This manuscript makes a nice start towards understanding how morphologies of phase separated systems are related on molecular level changes. I recommend publication after the following comments have been addressed:

We thank the referee for their positive assessment, especially recognizing the value of addressing morphology as a read-out of potential physical mechanisms. We address all comments below.

Major:
1. There are significant work on wetting behavior and what could shape of phase separated droplets could tell us about the physical state they are in. Could you give us an explanation/interpretation what is different about type i, ii and iii? Why do you observe these different shapes? What type of changes could explain these differences?

Cluster types: *One aspect of type i, ii, and iii clusters that we address in the revision is, which parts of chromatin participate in cluster formation? We find that type i clusters are the only clusters that can contain elongating Pol II, likely representing single genes (new Figs. EV4 and EV5). Type ii clusters have very little elongating Pol II, but contain high levels of regulatory regions (enhancers with H3K27ac mark); type iii also have high H3K27ac, together with genes that are elongated (Pol II Ser2P) directly on their surface of, but not inside the Ser5P-rich condensate (new Fig. 4).*

Mechanism for different shapes: *We now take apart in more detail how our theoretical model results in the observed shapes of the different cluster types (extensions to Fig. 5). This should hopefully explain better how the interplay of surface condensation of the Ser5P-rich condensate (red) on regulatory regions (blue), attraction between chains via the red phase, and unfolding due to exclusion of elongated genes (gray) together give the range of morphologies seen in our experiments.*

Physical mechanisms*: We now more directly assess the physical nature of the surface condensation, employing hexanediol treatment and assessment of live imaging time lapses, as also suggested by referee #1 (newly added Fig.3). These new data indicate that while interactions that can drive phase separation play a role, canonical liquid-liquid phase separation (LLPS) alone cannot explain the observed behaviors - a condensation surface provided by regulatory chromatin is needed to allow the condensate formation.*

2. Why is Pol II Ser2P levels are different than Pol II Ser5P level?

The levels of Pol II Ser5P in each cluster seem to be determined by how much regulatory chromatin (H3K27ac, or blue regions in the model) is present (new Fig. 4E, in the model Fig. 5H). The level of Pol Ser2P seems to be determined by how many elongated genes are placed close to the cluster surface (improved panel Fig. 2F, in the model Fig. 6).

Within type ii and iii clusters (large clusters), the Pol II Ser5P and Ser2P signals are distributed into mutually exclusive patterns. In our experiments, as in previous work, this is observed quite evidently (new Fig. EV4). Previous work attributed this exclusion to changes in molecular affinities of the Pol II CTD upon differential phosphorylation, or the production of transcript RNA, leading to exclusion from the Ser5P-rich condensate. Our work is agnostic to the mechanism, and simply implements this exclusion in the model in line with the observations. This assumption, together with the polymer chain structure present in our model, is sufficient to recapitulate these patterns, and even behaviors in live imaging experiments, so it seems to be a sufficient explanation (new panels in Fig. 5).

3. Could you explain what is the difference in lattice simulation output results of type i, ii and iii? What does it imply? Could you speculate?

We partially addressed this under the points 1 and 2 above. In addition, to test whether the model really captures how Ser5P and Ser2P lead to cluster morphology, we applied the inhibitors flavopiridol and triptolide. The effect of these inhibitors on phosphorylation can be included in our lattice model, and the model then indeed predicts the changes in cluster morphologies as observed in our experiments (Fig. 7). For flavopiridol, all clusters become more similar to the type ii because of losing elongation. For triptolide, the adhesion between chromatin chains is reduced, resulting from a compromised affinity within the condensates and from the condensates to the regulatory chromatin. So, it seems that the different parts of our model (surface condensation, multiple chains, exclusion of elongated genes) are necessary but also sufficient to explain how different morphologies are formed in both normal conditions and drug treatments. This is explained also in the Results text associated with Fig. 7 (lines 442-458).

The referee also raises the interesting question of what the implications of our findings could be. This is of course the realm of speculation, but we followed the question in adding this last paragraph to the Discussion section (lines 578-598):
“Finally, the question remains as to how far the observed clusters and their internal organization relate to the control of transcription. One key issue is the control of genes by enhancers over sequence distances of tens or even hundreds of kilobases. Proximity of an enhancer to a target

gene in three-dimensional space can, for example, trigger Pol II pause release (Bartman2016). Transient proximal placement of an enhancer within a distance of a few 100 nm is sufficient to induce transcription, whereas persistent association and direct molecular binding seem not to be required (Chen 2018, Li 2020). In line with these observations, the clusters of recruited Pol II in our work exhibit diameters of a few 100 nm, contain genomic regions that harbor enhancers, and are only occasionally visited by genes that undergo transcription. Together with recent in vitro observations of the zipping together of DNA strands by a liquid phase (Quail 2020), our work implies that this loose engagement of genes with enhancers might be facilitated by liquid bridges. During the time of engagement, such bridges would permit the transfer of transcription factors that are collected via the enhancer to the regulated gene, where they can be exploited for transcriptional activation even after the liquid bridge is broken (Cho 2018, Xiao 2020, Zuin 2021, Brandao 2021, Shao 2021, Trojanowski 2021). Liquid bridges between different regions of the genome can also serve as mechanical connections, contributing to the 3D organization of chromatin within and in the neighborhood of Pol II clusters (Nozaki 2017, Nagashima 2019}).”

Minor:

4. SI Figure 8 and 10 are never mentioned in the manuscript. If they are not significant and contributing the story, take them out. If not, please explain.

The Fig. EV2 (previously SI Fig. 8) assesses that a 2D analysis can be compared with a full 3D image analysis. The Fig. EV7 (previously SI Fig. 10) explains the lattice model algorithm implementation. Both points are crucial to substantiate the technical approaches that support our story. We now made sure to reference all supporting figures correctly.

Reviewer #3:

Review for: RNA polymerase II clusters form in line with liquid phase wetting of chromatin.
In Pancholi, Klingberg et al., the authors use a combination of confocal and superresolution imaging, lattice Monte Carlo numerical simulations, and a compartment model to understand how the morphologies and patterns of Ser5phos-RNAPII and Ser2phos-RNAPII are established in the developing zebrafish embryo. The study addresses a topic that is timely, but there are a number of major weaknesses. They posit that the several types of morphologies of both Ser5Phos and Ser2Phos can be understood by assuming that RNAPII acts as a cohesive liquid phase in the nucleus that binds to chromatin and "wets" the surface. There is no evidence from the data presented that this is the right physical picture, and the authors heavily rely on evidence from other work suggesting a phase separation mechanism of transcriptional machinery. This is in principle reasonable, except that the main message of the paper is precisely that RNA pol II makes a liquid phase that wets chromatin.

We appreciate these comments, it is obvious the referee is quite knowledgeable of the topic overall and also the ongoing, more specific debates of our field. We are glad to discuss the mentioned weaknesses, and how we addressed them in our revisions.

Major comments:

1. My main major concern for the experimental part of the study is related to the antibody-based images generated in Figs. 1 and 2. In Fig. 1B, raw and processed images are presented for Pol II Ser2Phos and Pol II Ser5Phos for a representative sphere stage nucleus. The examples given in panel C demonstrate that there are different morphologies but in general it appears that the Ser2Phos and Ser5Phos are overlapping. In Fig. 2A, there are images of pol II Ser5Phos generated

using STEDD and Ser2Phos using confocal microscopy. These images are taken from a late blastula stage zebrafish embryo. Firstly, why weren't embryos from the same stage-sphere vs late blastula-used? Why was it important to use differently-staged embryos or dissociated cells? In addition, the localization of the Pol II Ser2P, visualized using the full-length antibody, in Fig. 2A is qualitatively different from the localization in Fig. 1B, visualized with the FAB fragment. How can you explain these differences? An important experimental control would be to also use the labeled FAB fragment with the fixed samples in the late blastula stage. The problem is, the model assumptions/rules depend on Ser5 and Ser2 not co-localizing (as observed in Fig. 1, where the signals clearly co-localize). It is difficult to see the model's merit in the absence of this assumption, and, to my mind, this point-that Ser5 and Ser2 do not co-localize-has not been clearly experimentally demonstrated.

This comment makes a number of very good points which we address below.

Developmental stage: *All embryos in our study were imaged at the sphere stage. The sphere stage occurs during the late blastula phase of development of the zebrafish embryo, so we used these expressions interchangeably. In other words, all embryos are assessed in the same stage throughout, only our nomenclature was inconsistent. We are sorry about the confusion and fixed this in the revised manuscript.*

Dissociation into primary cell cultures: *We dissociate embryos to obtain primary cell cultures for the purpose of applying inhibitors, as was done in our (Hilbert et al. Nature Comms 2021) and others (van Boxtel et al Dev Cell 2015) previous work. The inhibitors, when applied to intact embryos, often do not permeate the embryo, require 10-20 times the recommended concentration, or exhibit slow and inconsistent permeation. The dissociation into primary cultures, as stated, is standard procedure, and, as we assessed previously, does not interfere with cell division, chromatin organization, or transcriptional activity when following appropriately controlled protocols (Hilbert et al. Nature Comms 2021).*

Pol II Ser5P/Ser2P overlap in live imaging data: *All images in Fig. 1 are obtained from live embryos, using antibody fragments (Fab). In these images, the Pol II Ser2P signal is indeed overlapping with the clusters seen in the Ser5P channel. We did not discuss this overlap, because the Ser2P channel does not give sufficient signal-to-noise ratio, and accordingly insufficient effective image resolution to reliably assess overlap. This issue is due to the limited signal-to-noise ratio of the Pol II Ser2P Fab, that is in turn limiting the effective resolution, as is also documented in another study that uses the same Fab (Forero-Quintero et al. 2021). In the immuno-stainings, the signal-to-noise is much better, so the Pol II Ser2P channel can be better assessed. We address this point in the following statement (line 177-180, unchanged from original submission):*
"The improved signal-to-noise ratio relative to our live imaging data revealed an additional detail: the elevated Pol II Ser2P signal associated with type iii clusters was placed directly adjacent to, but did not strongly overlap with the Pol II Ser5P signal (Fig. 2B)."
The resolution in the Pol II Ser2P is yet further improved in the newly added two-color STED images (new Fig. EV4). Here, the exclusion between Pol II Ser5P and Pol II Ser2P is also apparent for type ii and iii clusters, while overlap can be seen for type i clusters (new Fig. 5), see also the point directly below.

Additional imaging to clarify Pol II Ser5P/Ser2P colocalization: *To further validate the assumption that Pol II Ser5P and Pol II Ser2P are mutually exclusive, we carried out STED imaging with full 3D-STED depletion for both Pol II Ser5P and Pol II Ser2P (new Fig. EV4). This approach does not deliver the pristine resolution of Pol II cluster morphology afforded by STEDD for the Pol II Ser5P channel, but instead reveals a much more detailed picture of the spatial relationship of the*

Ser5P and Ser2P signals with each other. Specifically, we find that Pol II Ser2P signal is almost completely excluded from large Pol II Ser5P clusters (type ii and iii), but super-imposes on a fraction of small Pol II Ser5P spots (type i). Thus type i clusters might well represent single genes located outside the large clusters.

Imaging of fixed, Fab-injected embryos: *The referee requested an analysis of Fab distribution in fixed samples. We fixed and imaged Fab-injected embryos. We show the results only here for the referee. The gating analysis suggests the same general relationship between cluster morphology and phosphorylation levels obtained by all other approaches (see Figure below). The image quality, however, was by far the worst out of all our different ways to image Pol II Ser5P and Ser2P, so that even when pooling from several embryos, only a small number of clusters could be reliably detected. We therefore did not want to include these data in the manuscript.*

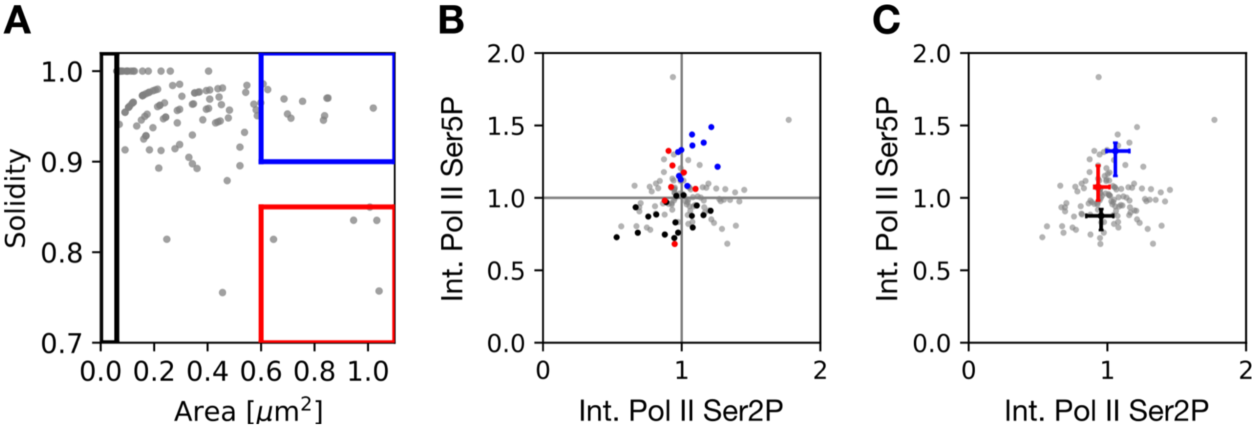

Gating analysis of cluster types i, ii, and iii, pooled from 5 fixed embryos that were injected with antibody fragments (Fabs). Analysis procedures and display identical to Fig. EV3.

2. The authors use the assumption that there is a repellant effect between Ser5Phos and Ser2Phos, a notion motivated by imaging data, and they also include modifications to the polymer chain in simulations to mimic the effect of active and inactive regions of chromatin. However, it is also unclear what is the value of such simulations, what do we learn from them? The ingredients of the simulation are just chosen such that Ser5Phos and Ser2Phos repel each other, and that they want to bind to different regions. The results of the simulations are then not really unexpected. I also couldn't find any quantitative comparison between the data and the simulation that would validate the main message of the paper, nor a prediction that challenges it. The accompanying 1d model is also too primitive for the purpose, why the capacity of the droplets saturates? Why don't they grow unbounded or what bounds their growth? Some authors have also recently challenged the notion that transcriptional machinery phase separates on chromatin, and instead classic binding and unbinding could explain these transcription hubs. This work relies too heavily on the assumption that the problem at hand involves liquids, but at the same time it is the conclusion they want to drive home. From the data and simulations presented this is just not proven.

Again, the referee makes a number of very good points, all of which we address below. Our additions provide a validation of all our model assumptions by experiments, as well as a list of numerous experiments that are reproduced by our model - without further adjustments to our model assumptions or parameters relative to our original submission.

Value of simulations: *Considering the referee's awareness of the current uncertainties regarding how molecular clustering, interactions that can drive phase separation, and chromatin are related, it*

should also be clear how important it is to address this very relationship. Clarifying this relationship, even only to some extent, seems highly valuable to us. Our study provides a model that not only agrees with a very comprehensive set of experimental data, but also explains how this relates to clustering, condensate formation, and the role of chromatin polymers. Every model starts with a set of initial assumptions informed by the experiments and a hypothesis about the major underlying physical mechanisms. This then can be tested by generating model predictions that should be verified in the corresponding perturbation experiments. Specifically, our model describes the interaction of a subsaturated liquid phase with a chromatin surface. This concept can reconcile two crucial observations from nuclear clusters: limited growth of condensates and dynamic merging and separation of clusters. Both observations are in contradiction with a liquid-liquid phase separation (LLPS) scenario that is currently predominantly discussed in chromatin literature. Our model explains these observations. Also, the concept of surface condensation is still extremely novel to chromatin biology/biophysics: there are three preprints that address this concept in vitro, currently under review or in press. Our model even extends on this new theory, adding the element of exclusion from the condensate, and thus is an essential tool helping to design proper tests of these surface-condensation concepts in vivo. In our understanding, using a model to critically test in vivo, in a vertebrate embryo, a new mechanism of organizing transcriptional regulation represents a highly valuable achievement of the model.

Model assumption of Pol II Ser5P/Ser2P repulsion: *This assumption stems from previous work that directly observed the mutual exclusion in vitro (Lu et al. Nature 2018) and in vivo (Guo et al. Nature 2019). To better back up this assumption in the context of our experimental model, we added new experiments, where we assess the separation of Pol II Ser5P and Ser2P in much more detail (new Figs. EV4, EV5). We also apply oligopaint DNA-FISH to demonstrate that Pol II Ser5P and Pol II Ser2P drive the inclusion and exclusion of chromatin regions, respectively (new Figs. 4A-C,F,G and EV6).*

Comparison of data and predictions relating to the main message of the paper: *The referee states they could not find a* "quantitative comparison between the data and the simulation that would validate the main message of the paper". *The main message of our model is that (1) clusters form by a combination of affinity of the Pol II Ser5P-rich phase for itself as well as for regulatory chromatin, and (2) that the Pol II Ser5P-rich clusters unfold due to the presence of transcribed genes. Both points are directly tested by the treatment with flavopiridol and triptolide, and the effects on cluster shape are predicted one-to-one by our model simulations (Fig. 7). In particular, flavopiridol removes the contribution of transcribed genes (block of pause-release), resulting in compaction of clusters, in the experiments and in the simulations. Triptolide removes the contribution of transcribed genes, and additionally reduces the affinity of the Pol II Ser5P for itself and for chromatin, resulting in unfolding and splitting of clusters, in the experiments and in the simulations. Taken together, these experiments and simulations directly assess the main mechanisms of surface condensation and exclusion of transcribed genes, and should therefore address this concern. These results were already contained in our original submission. We have now rearranged the results into one figure, so that they are easier to find (newly structured Fig. 7) and edited the Results text to make this point clearer (417-458).*

If the referee's statement refers to a lack of strictly quantitative data (lengths, cluster volumes, molecule counts, concentrations, kinetics), we fully agree that all our simulation results only connect to the experimental data in a semi-quantitative way (fold changes, correlations between different measurement variables, relative time scales, obvious behaviors such as lack of growth and droplet fusion). Given the context of a vertebrate embryo, such strictly quantitative comparisons are relatively hard to achieve, and we decided to instead aim for a comprehensive set of semi-quantitative comparisons. However, we should note that the important parameters of the model

such as interaction energies and size of a single lattice site related to microscopy resolution are very similar to the parameters of the models we previously used for description of microphase separation process in the context of the chromatin (Hilbert et al., Nature Comm 2021) (lines 1109-1121). This provides confidence that the model operates in the range of biologically relevant parameters.

Further, the referee states that there is no experiment that challenges our model. We disagree - already our original submission provided experiments with actinomycin D, an inhibitor with a mechanism of action not primarily related to Pol II CTD phosphorylation, and, consistent with our expectation, find that our model fails to predict the effects of this treatment on cluster shape (lines 459-472 in the revised manuscript).

Removal of 1D compartment model: *We agree with the referee that a 1D compartment model (Fig. 5 of the original submission) is insufficient to model spatial organization and removed this model.*

Limited growth of condensates / mechanism of cluster formation: *Already in our original submission, we proposed a mechanism that is distinct from canonical liquid-liquid phase separation, namely the phase condensation on a polymer surface at subsaturated bulk concentration (lines 353-368 in the revised manuscript). We now also explain that affinity within the liquid phase and the amount of available surface dictate the maximum size a cluster can grow to, which are hallmark features of this type of condensation (lines 387-399)). Further, we added new imaging data showing that, indeed, the concentration of enhancer chromatin in a given cluster is correlated with the size of that cluster (Fig. 4E), which is fully in line with this model (Fig. 5H). Also, we showed this limited growth scenario with additional live-imaging time-lapses (Fig. 3E). Another behavior visible in time-lapses are connection/splitting cycles between segments of the Pol II clusters (Fig. 3F). Both observations cannot result from canonical LLPS alone, but could be reproduced one-to-one by additional plots of our model simulations (Fig. 5E,F). We also mention in our introduction that the morphology of clusters cannot be explained by canonical LLPS alone (lines 103-106):*
"While LLPS was suggested as part of the mechanism for the formation of Pol II-enriched clusters, these clusters exhibit complex morphologies that deviate markedly from the round, droplet-like shapes typical of canonical LLPS (Brangwynne 2009, Eskiw 2008, Cho 2018, McSwiggen 2019).

The referee also correctly points out that there is an ongoing discussion of the mechanism underlying the formation of Pol II clusters in live cells. One point where discrepancies can be seen throughout the published literature is that, on the one hand, interactions typical of liquid-liquid phase separation seem to contribute to cluster formation (see above), and in vitro experiments support this picture. On the other hand, the observed clusters do not grow into well-defined droplets but are instead limited to small size, and are also granular and not smooth in morphology.

The cluster size is determined by the amount of available condensation surface, as was already shown in our originally submitted manuscript (Fig. 5B in the revised manuscript). Based on new live-imaging data (new Fig. 3), typical behaviors such as droplet fusion or Ostwald ripening cannot be observed. Instead complex cluster shapes are apparent, which maintain the same configuration over more than ten minutes. All of these observations are clearly in contrast with a typical liquid-liquid phase separation scenario. They are, however, all explained by our theoretical model in a consistent manner.

Transcription hubs from binding/unbinding to target regions: *This picture of transcription hubs based only on binding/unbinding suggested by the referee indeed seems to apply - for the case of the spots formed from elongating Pol II. We find in our new two-color STED data that these spots*

might represent single genes, as proposed also by referee #1 (new Fig. EV4). In agreement with this interpretation, the newly added hexanediol treatment data (new Fig. 3C,D) imply that spots of elongating Pol II are not formed by liquid phase condensation, but most likely represent Pol II engaged with the DNA template during transcript elongation.

Assumption of a liquid phase: *We can understand the referee's criticism, given a somewhat overwhelming number of studies that simply assume a LLPS scenario as a relevant mechanism. As stated above, the new experiments with hexanediol give some insights here. First, in the case of elongating Pol II (Pol II Ser2P), the application of hexanediol does not seem to perturb the spot pattern formed by Pol II with this label (new Fig. 3C,D). Accordingly, in this case, a contribution of weak interactions typical of a liquid phase seems unlikely.*

The hexanediol treatment does, however, perturb the clusters enriched in recruited Pol II (Pol II Ser5P): the clusters split into smaller pieces in some cases, and in other cases unfold (new Fig. 3A,B). Our interpretation is that weak interactions driving phase separation therefore have some contribution to the formation of these Pol II Ser5P-enriched clusters, but cannot fully account for their formation. Instead, by our added experiments on regulatory chromatin (Fig. 4) and our simulations indicate that a combination of these weak interactions within the Pol II Ser5P-enriched phase together with an affinity of the Pol II Ser5P-enriched phase for regulatory chromatin regions can explain the observed cluster formation, the outcomes of hexanediol and also inhibitor treatments.

Distinguishing this scenario where regulatory chromatin provides a surface for condensation from a scenario based on canonical LLPS alone is a major point of our work. This distinction from LLPS can also be seen by the newly added live imaging data, which show Pol II Ser5P clusters that do not grow, and do not fuse or ripen into larger droplets (new Fig. 3E,F for experiments and Fig. 5D,E for the model). Further phenomena that are explained by our surface-condensation model are the following:

- *morphological types i-iii, as well as their dependence on phosphorylation (Fig. 2 vs. Fig. 6)*
- *different responses of Pol II Ser5P and Ser2P patterns to hexanediol (Fig. 3A-D vs. Fig. 5F)*
- *Increase of cluster area and solidity with H3K27ac intensity (Fig. 4E vs Fig. 5G)*

We have also gone into much detail how our model assumptions are justified by direct experimental verification (new Figs. 3, 4, EV4, EV5, EV6, EV11, see other responses to this referee as well as referees #1 and #2). Of course, one can never rule out other models - but once all assumptions and predictions of a given model are verified, it is typically seen as trustworthy. Seeing that we have verified all assumptions and predictions, we hope the referee can also conclude that our model, even if it is not the only possible model, is sufficiently validated.

Thank you for sending us your revised manuscript. We have now heard back from the two referees who were asked to evaluate your revised study. As you will see below, the reviews think that the study has improved as a result of the performed revisions. However, reviewer #3 still raises some remaining concerns, requesting some additional controls and explanations to better support the main conclusions. We would ask you to address these issues in a second round of revision.

Moreover, we would ask you to address some remaining editorial issues listed below.

REFEREE REPORTS

Reviewer #1:

For the revised manuscript, the authors generated new data to comprehensively address the concerns. They could mitigate them to a large extent, or revised their original statements accordingly. This strengthened the manuscript substantially; especially the association studies of the Ser5P-/Ser2P-clusters with H3K27ac and super-enhancer regions (oligopaint) is a valuable and interesting addition in this context and supports an important conclusion. From my side, the manuscript is acceptable for publication.

Reviewer #3:

Referee report:

I appreciate the efforts to respond to my and the other referee's concerns. However, I still think my main concerns were not properly addressed as you will see below:

Major comments:

(1) I must maintain the position that it is problematic that the live-imaging experiments performed with Ser5P and Ser2P FAB fragments (Fig. 1B) are clearly overlapping and that the fixed samples imaged with full-length antibodies and STED microscopy are not (Fig. 2). In principle, the authors argue that the images using STED have improved signal-to-noise, but the authors have not presented convincing evidence that this is true. To my eye it appears clearly that the staining for Ser2-Phos is different than the live imaging data. It is slightly concerning that only the analyzed data and not the raw imaging data for the fixed Ser2P Fab fragment data was included in the referee report. I would strongly encourage the authors to use a different full-length antibody if at all possible, optimize the staining, and perform these experiments in the presence and absence of a transcriptional inhibitor, so that we can be satisfied that the results presented in Figure 2 are true. An additional control that would assuage doubts about possible artefactual stainings would be to exploit the onset of the mir430 foci at early cell stages as reported in recent studies including Hilbert et al. 2021. The Fab fragments will certainly localize strongly to these two foci (as previously reported) in a txn-inhibitor-dependent fashion. Can the authors also perform fixed stainings at these early stages using the full-length antibody and demonstrate that, in the presence of a txn inhibitor, there are no foci? It is essential because the underlying basis of the model, the key ingredients, rely on this immunostaining in Fig. 2 and I feel that this must be addressed and strongly controlled for.

(2) The authors have also not really addressed the issue of showing that surface condensation (as a pre-wetting transition in the new iteration) is the mechanism at play. In my previous report I asked whether simple binding to specific regions was sufficient to explain the fluorescent data (a model currently proposed by some groups in the field), which has nothing to do with phase separation. Such model would lead to co-localization of signal with DNA, which would lead to similar morphologies as observed here given the impossibility to resolve single molecules. The authors need to rigorously consider this as a possible explanation of their experiments. Another major issue is that the authors try to distinguish LLPS from pre-wetting transitions (fig 5B), and just pick three different values of interaction energy and assign them to surface absorption, condensation and LLPS. How are these assignments done? It does look that it is simply based on how the simulations look like, but there is a very quantitative and defined way to show whether the observations in the simulation correspond to LLPS or a pre-wetting transition. Finally, the authors claim they have predictions of the model that relate to the perturbations. However, here again they change the parameters of the simulation somewhat arbitrarily (or simply so that it corresponds to the

experiments) by for example changing the interaction parameter and particle number in the triptolide perturbation. This is not a prediction.

Minor comments:
(1) Line 135 - what do you mean by tolerated well? Please explain.
(2) I find the reference to an "unfolded" shape to be misleading. Do you mean elongated? Complex geometry? I find that unfolded implies something about the stability of the protein, which I don't think is relevant in this context.
(3) Hexanediol effect is hard to interpret and the field does not consider this as a proof of phase separation anymore.

Reviewer #1:

For the revised manuscript, the authors generated new data to comprehensively address the concerns. They could mitigate them to a large extent, or revised their original statements accordingly. This strengthened the manuscript substantially; especially the association studies of the Ser5P-/Ser2P-clusters with H3K27ac and super-enhancer regions (oligopaint) is a valuable and interesting addition in this context and supports an important conclusion. From my side, the manuscript is acceptable for publication.

We appreciate the referee’s positive response.

Reviewer #3:

Referee report:

I appreciate the efforts to respond to my and the other referee's concerns. However, I still think my main concerns were not properly addressed as you will see below:

We sincerely hope that this time we understood the referee’s concerns, and appreciate the additional explanations to get us on the right track. We hope the referee is now convinced of our conclusions based on the additional materials presented below.

Major comments:

(1) I must maintain the position that it is problematic that the live-imaging experiments performed with Ser5P and Ser2P FAB fragments (Fig. 1B) are clearly overlapping and that the fixed samples imaged with full-length antibodies and STED microscopy are not (Fig. 2). In principle, the authors argue that the images using STED have improved signal-to-noise, but the authors have not presented convincing evidence that this is true. To my eye it appears

clearly that the staining for Ser2-Phos is different than the live imaging data. It is slightly concerning that only the analyzed data and not the raw imaging data for the fixed Ser2P Fab fragment data was included in the referee report. I would strongly encourage the authors to use a different full-length antibody if at all possible, optimize the staining, and perform these experiments in the presence and absence of a transcriptional inhibitor, so that we can be satisfied that the results presented in Figure 2 are true. An additional control that would assuage doubts about possible artefactual stainings would be to exploit the onset of the mir430 foci at early cell stages as reported in recent studies including Hilbert et al. 2021. The Fab fragments will certainly localize strongly to these two foci (as previously reported) in a txn-inhibitor-dependent fashion. Can the authors also perform fixed stainings at these early stages using the full-length antibody and demonstrate that, in the presence of a txn inhibitor, there are no foci? It is essential because the underlying basis of the model, the key ingredients, rely on this immunostaining in Fig. 2 and I feel that this must be addressed and strongly controlled for.

As the referee still has concerns with regard to overlapping signals between Pol II Ser5P and Ser2P Fab labeling, we took several steps, which hopefully can give assurance that the fixed sample Pol II Ser2P staining and also overall immunofluorescence is trustworthy. Furthermore, as we inspected images of fixed Fab samples more thoroughly, we could now recover the non-overlapping Pol II Ser2P pattern with spots throughout the nucleus - likely because in the fixed Fab samples we could record the two color channels sequentially, not simultaneously, thereby preventing spectral crosstalk.

The experiments are quite extensive, and all address the point of technical reliability. Therefore, we have added a new paragraph in regard to this point, which we provide here along with the four supporting Appendix Figures that were newly added or extended.

Added paragraph:
“To rule out that conclusions from the analysis of the STEDD microscopy data are affected by technical artefacts, we carried out several control experiments. All results obtained by the analysis of STEDD images were reproduced, albeit at lower resolution, in an analysis of our live imaging data (Appendix Fig. 3A-C). Fixation of Fab-injected embryos allowed instant-SIM microscopy with improved signal-to-noise ratio and temporal separation of color channels, thereby revealing a pattern of Pol II Ser2P spots placed adjacent to Pol II Ser5P clusters (Appendix Fig. 3D) that resembles the pattern seen in our STEDD micrographs (Fig. 2A,B). The data obtained from these fixed embryos also reproduced the results obtained by the gating-based analysis of STEDD data (Appendix Fig. 3E-G). To verify the specificity of the Pol II Ser2P immunofluorescence detection, we also recorded data from embryos fixed at the oblong stage of development. The oblong stage precedes the sphere stage, and in the minutes directly following cell division, nuclei exhibit prominent clusters of elongating Pol II that are associated with microRNA miR-430 transcription (Hadzhiev 2019, Chan 2019, Hilbert 2021). Staining with the same primary antibodies used to label samples for STEDD microscopy, we indeed observed the expected prominent clusters of Pol II Ser5P and Pol II Ser2P signal (Appendix Fig. 4). Note that, in line with a conversion from morphology type ii to morphology type iii, the shape of these prominent clusters seen in the Pol II Ser5P channel was more unfolded for higher levels of the Pol II Ser2P signal (Appendix Fig. 4). The specificity of the antibody used to label Pol II Ser2P for STEDD microscopy was further confirmed by treatment of whole embryos with

the transcription inhibitor flavopiridol, which indeed largely abolished the Pol II Ser2P signal (Appendix Fig. 5A,B}). Flavopiridol treatment had the same effect, regardless of exchange of all primary antibodies, secondary antibodies, and fluorophores in the immunofluorescence protocol by alternative ingredients (Appendix Fig. 5C,D). To control for artefacts only appearing with super-resolution microscopy, we carried out two-color STED microscopy for samples labeled with both alternative sets of antibodies and fluorophores (Appendix Fig. 6A-B). An analysis based on gates for the morphology types i-iii reproduced the relationship of cluster morphology to Pol II phosphorylation (Appendix Fig. 6C-H) detected in data acquired by STEDD microscopy (Fig. 2C-E). These control experiments support the validity of the conclusions drawn from the analysis of STEDD microscopy data."

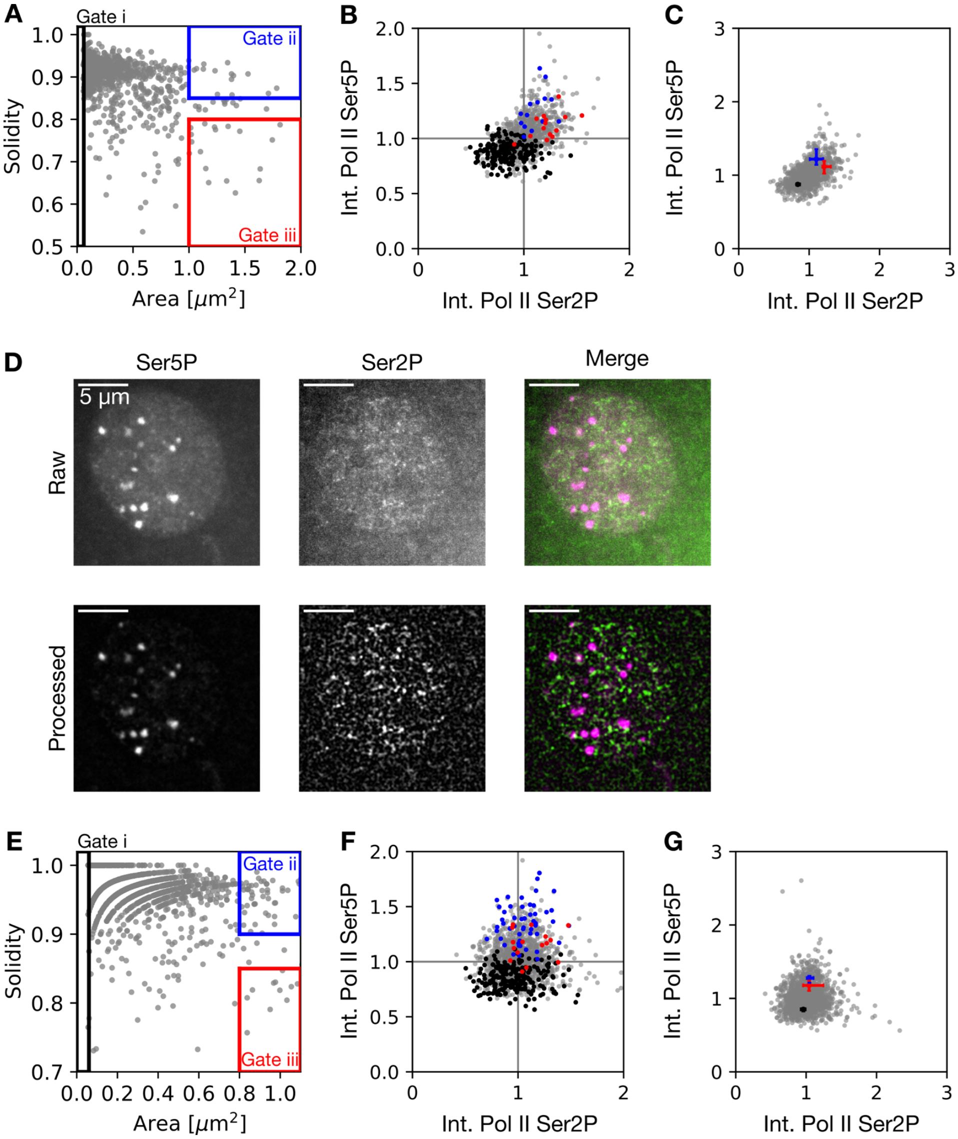

Appendix Figure 3: Cluster morphology types are also detected in live embryos and fixed embryos injected with antigen-binding fragments. A) Cluster properties were extracted from images obtained from live zebrafish embryos (sphere stage) using antigen-binding fragments (for details, see Materials and Methods). Area and solidity of the individual clusters, with gates showing type i, ii and iii clusters. B) Phosphorylation levels (serine 2 and serine 5 phosphorylation) of the individual clusters (mean fluorescence intensity). The values are normalized to the median of each analyzed nucleus. Number of nuclei: n=109. Number of clusters: n=959. C) Median Pol II Ser2P and Ser5P intensities with 95% bootstrap confidence intervals (10,000 resamples). D) Representative maximum-intensity projection of a nucleus of an embryo injected with antigen-binding fragments prior to fixation. Raw data were processed by local background subtraction and smoothing. E-G) Same analysis as in panels A-C, applied to image data obtained from fixed embryos injected with antigen-binding fragments. Number of nuclei: n=177. Number of clusters: n=1326.

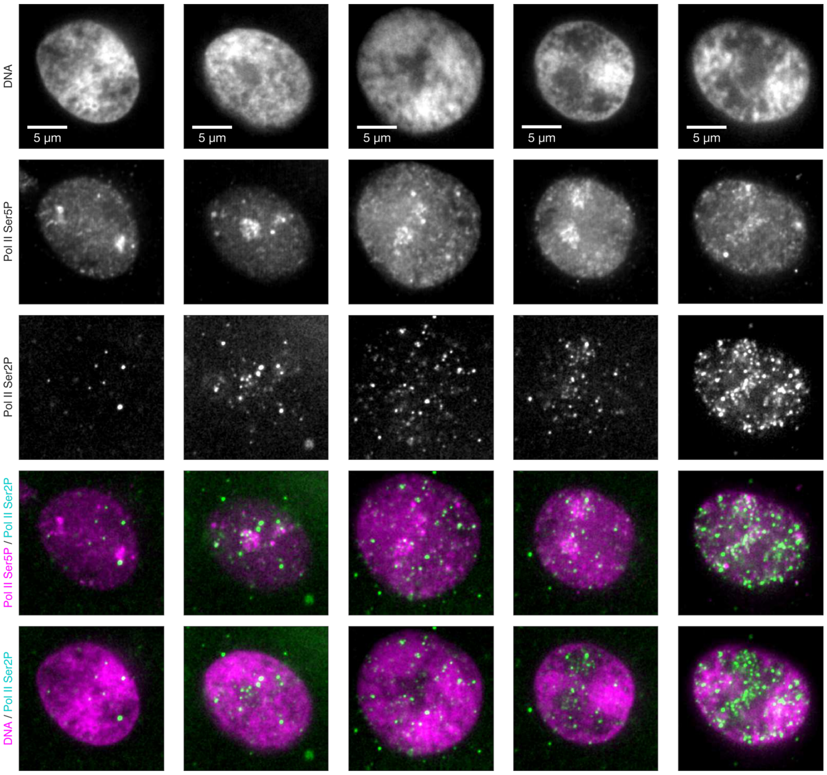

<u>Appendix Figure S4: Recruited and elongating RNA polymerase II is detected in microRNA miR-430-associated foci.</u>Micrographs of nuclei in a zebrafish embryo fixed in the oblong stage, showing prominent clusters of recruited RNA polymerase II (Pol II Ser5P) and elongating RNA polymerase II (Pol II Ser2P) associated with the transcription of the miR-430 microRNA cluster. Images recorded by instant-SIM microscopy, single z-sections are shown, Pol II Ser5P and Pol II Ser5P labeled via immunofluorescence (antibody set 1), DNA labeled with Hoechst 33342. Images were sorted by eye, based on the apparent progress of the transcription onset and DNA domain coarsening that follows cell division.

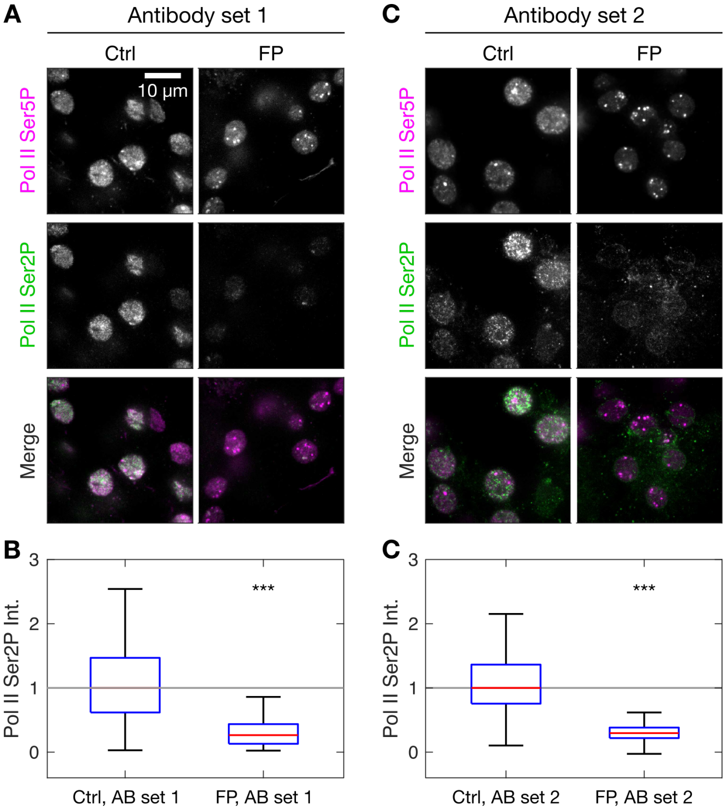

Appendix Figure S5: Verification of RNA polymerase II serine 2 phosphorylation immunodetection. A) Representative micrographs of fixed sphere stage embryos that were untreated (Ctrl) or treated with flavopiridol (FP, 10 $\mu \mathrm{M}$, 90 min) and labeled by immunofluorescence. Images are single instant-SIM confocal sections. Main antibody set (AB set 1): primary antibodies -- mouse IgG anti-Ser5P, rabbit IgG anti-Ser2P; secondary antibodies -- anti-mouse IgG STAR RED, anti-rabbit IgG STAR ORANGE. B) Quantification of mean Pol II Ser2P intensities inside nuclei, AB set 1. Segmentation based on Pol II Ser5P channel, intensity values normalized to the median of the control condition. Two-tailed permutation test for Pol II Ser2P intensity differences, *** indicates $P<0.001$ ($P<0.0001$, $n=409,91$ nuclei from five and three embryos). C) Same as panel A, but for an alternative set of primary and secondary antibodies (AB set 2): primary antibodies -- rat IgG anti-Ser5P, mouse IgM anti-Ser2P; secondary antibodies -- anti-rat IgG Alexa 647, anti-mouse IgM Alexa 594. D) Same quantification as in panel B, but for AB set 2 ($P<0.0001$, $n=106,147$ nuclei from two and three embryos).

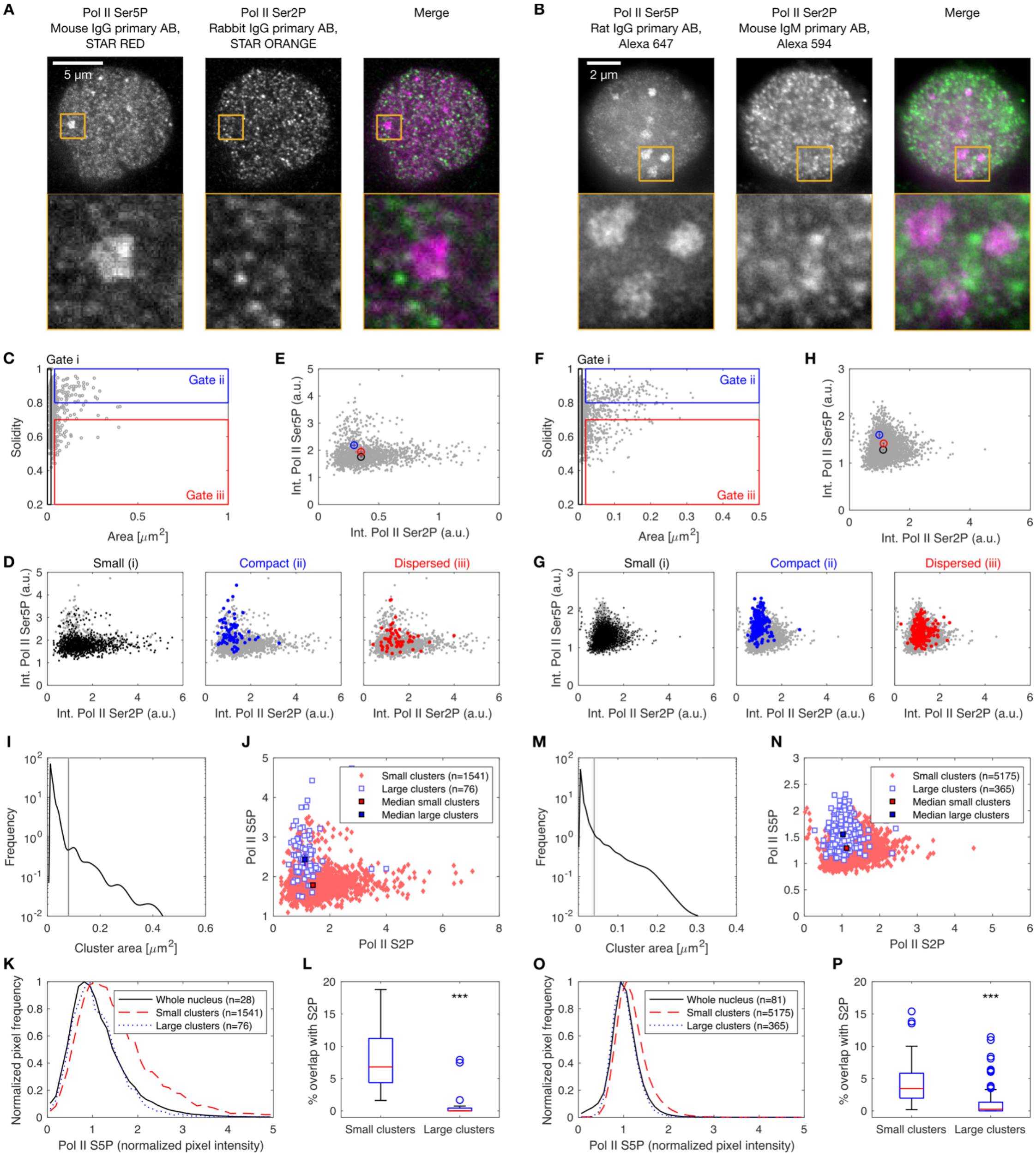

Appendix Figure S6: Assessment of Pol II Ser5P and Pol II Ser2P localization by 3D-STED with two alternative sets of primary antibodies and fluorophores. *A) Representative two-color 3D-STED section through a nucleus in a fixed sphere-stage zebrafish embryo. Immunofluorescence based on the primary antibody combination used throughout this study for combined Pol II Ser5P and Pol II Ser5P detection (AB set 1, rabbit mono-clonal IgG anti-Pol II Ser2P and mouse mono-clonal IgG anti-Pol II Ser5P) and secondary antibodies conjugated with the STED-optimized fluorophores STAR RED and STAR ORANGE, respectively. 100% 3D-depletion was used. B) Same as panel A for immunofluorescence with an alternative combination of primary antibodies (AB set 2, mouse IgM anti-Pol II Ser2P and rat IgG anti-Pol II Ser5P) and secondary antibodies conjugated with the STED-compatible fluorophores Alexa 594 and Alexa 647, respectively. 3D-STED depletion reduced to 25% to compensate for weaker fluorophore signal. C) Area and solidity of clusters detected in the Pol II Ser5P channel overlaid with gating areas for cluster types i-iii. Maximum area for gate i: 0.02 µm^2, minimum area for gates ii and iii: 0.04 µm^2, maximum solidity for gate iii: 0.7, minimum solidity for gate ii: 0.8. D) Mean Pol II Ser5P and Pol II Ser2P*

*intensity of all clusters (gray) overlaid with the intensities of those clusters within the gates applied in panel C. E) Median Pol II Ser5P and Pol II Ser2P intensities of clusters within the gates applied in panel C with bootstrap 95%-confidence intervals (10,000 resamples). F-H) Same as panels C-E, but for AB set 2. Modified maximum area for gate i: 0.01 μm^2, modified minimum area for gates ii and iii: 0.02 μm^2. I) Probability distribution of the area of Pol II Ser5P clusters. A cut-off at 0.08 μm^2 divides two populations visible in the logarithmic plot. J) n=1541 small and n=76 large clusters were extracted from images of n=28 nuclei from three embryos. K) Per-pixel Pol II Ser2P intensity levels throughout the entire nucleus and within large and small Pol II Ser5P clusters. L) Percentage of all Pol II Ser2P-positive pixels (robust background threshold with 3.0 standard deviations after background subtraction with range 1.0 μm) overlapping with small or large clusters. Two-tailed permutation test for differences in overlap, *** indicates P<0.001 , (P<0.0001, n=28 nuclei). M-P) Same as panels I-L but for antibody set 2. Area cut-off 0.04 μm^2, n=5175 small and n=365 large clusters from n=81 nuclei from two embryos (P<0.0001, n=81 nuclei).*

(2) The authors have also not really addressed the issue of showing that surface condensation (as a pre-wetting transition in the new iteration) is the mechanism at play. In my previous report I asked whether simple binding to specific regions was sufficient to explain the fluorescent data (a model currently proposed by some groups in the field), which has nothing to do with phase separation. Such model would lead to co-localization of signal with DNA, which would lead to similar morphologies as observed here given the impossibility to resolve single molecules. The authors need to rigorously consider this as a possible explanation of their experiments.

We are not at all against binding to chromatin regions as an explanation, in fact, it is a crucial part of our model and generally an exciting direction of work. Following the referee's comment, we added simulations of a "binding-only" version of the theoretical model. These show that binding indeed is sufficient to get most of the cluster morphologies; however, large, compact clusters only emerge with a model that incorporates liquid phase properties. Here are the additional paragraph and Appendix Figure:

Added paragraph:
"Our theoretical model includes interactions of red particles with regulatory chromatin as well as particle-particle interactions. Several reports on the role of transient particle-chromatin binding (Sabari 2018, Cho 2018, McSwiggen 2019, Shrinivas 2019, Chong 2018, Gibson 2019, Li 2020, Trojanowski 2021, Zuo 2021) raise the question of whether a ``binding-only'' model is sufficient to explain cluster formation. To implement such a binding-only scenario, we removed interactions between red particles ($w_{S5P\text{-}S5P}$=0) and restrained particle-chromatin interactions to be strictly local. Without further change to any of the other interactions, the size of the clusters is markedly reduced relative to the full model (Appendix Fig. S13A). A ten-fold increase in particle affinity ($w_{RC\text{-}S5P}$=-5.0) for regulatory chromatin can compensate for the reduction in cluster size, but the relationship between cluster size (area) and morphology (solidity) is changed relative to the full model (Appendix Fig. 13B). In particular, large clusters in the binding-only model exhibit markedly reduced solidity, both for unmodified and ten-fold increased particle-chromatin affinity (Appendix Fig. 13C). A binding-only model can thus explain cluster formation in general, but does not reproduce the large and compact clusters (type ii morphology) seen in our data (Fig. 2B-E). Our full theoretical model, which includes interactions between the particles that bind to regulatory chromatin, does produce such large clusters with compact morphologies. Accordingly, our investigation implies that clusters form by association of recruited Pol II with regulatory chromatin, and are enlarged and compacted by interactions amongst the particles that form these clusters."

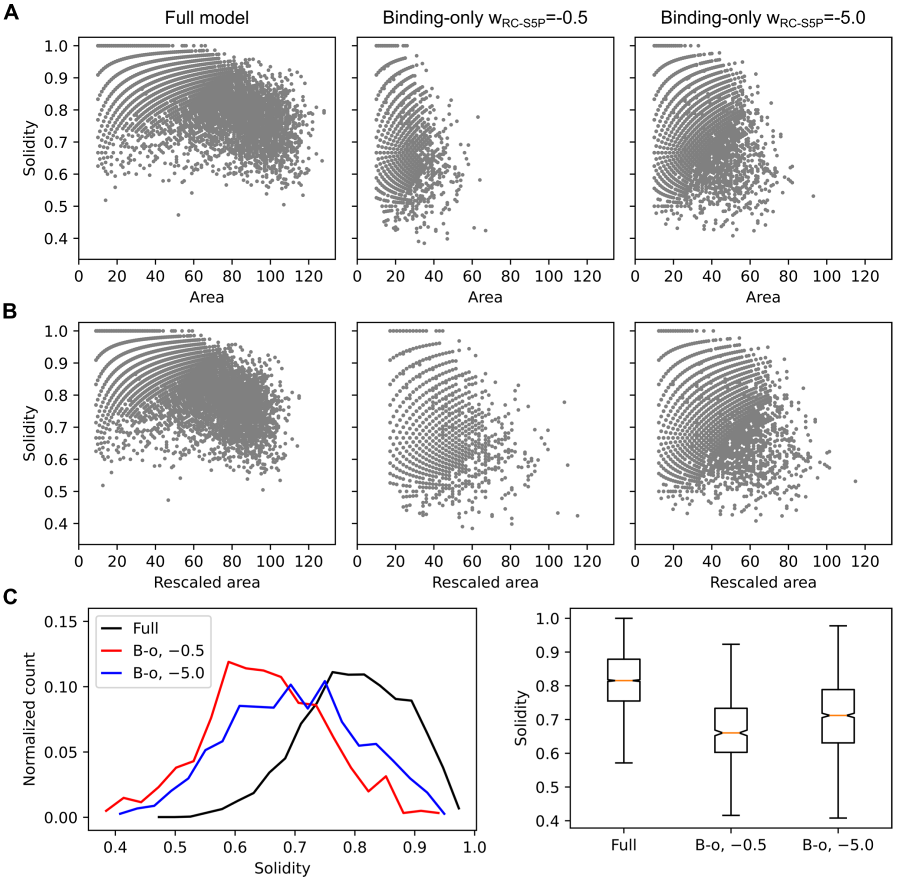

Appendix Figure S13: Comparison of the full theoretical model and the binding-only model.
A) Scatter plot of cluster solidity vs. cluster area for the full model (original parameters $w_{RC\text{-}S5P}$=-0.5 and $w_{S5P\text{-}S5P}$=-0.35) and the binding-only model ($w_{S5P\text{-}S5P}$=0 for both cases and $w_{RC\text{-}S5P}$=-0.5 or -5.0 as indicated). B) Rescaled scatter plots for the data shown in panel A. Cluster area was linearly rescaled so that the mean value of the top 1% percent cluster area was the same as in the full model simulation. Solidity was not rescaled. C) The solidity of large clusters (rescaled area>50) visualized as a histogram (plotted as line chart, left) and a boxplot (right)

Another major issue is that the authors try to distinguish LLPS from pre-wetting transitions (fig 5B), and just pick three different values of interaction energy and assign them to surface absorption, condensation and LLPS. How are these assignments done? It does look that it is simply based on how the simulations look like, but there is a very quantitative and defined way to show whether the observations in the simulation correspond to LLPS or a pre-wetting transition.

We made two additions here to address this point. First, to explain how we chose the value for the red-red affinity, we provide an additional analysis of how large clusters emerge with and without polymer surface present, to justify the parameter value we chose (wS5P-S5P=-0.35). Here is the new Appendix Figure:

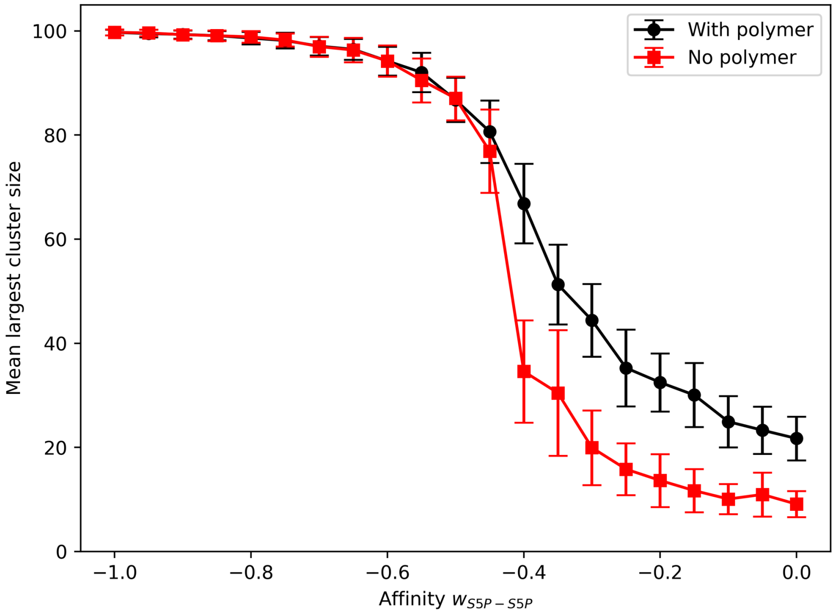

Appendix Figure S11: Dependence of cluster formation on particle-particle interactions and the presence of a polymer as a condensation surface. Mean largest cluster size (shown as number of particles in the largest contiguous cluster) of simulations with varying affinity $w_{S5P\text{-}S5P} \in [-1.0,0.0]$. Total number of red particles is constant N_{S5P} = 100) and 200 snapshots were obtained from the time series of a given simulation to calculate the mean of each affinity parameter. Error bars represent standard deviation. Simulations were performed for the case with no polymer (red, only S5P particles) and with a single polymer (black, $L_{polymer}$=20, N_{RC}=8 and N_{IC}=12).

Second, we took the referee's comment as a motivation to examine our model to more clearly explain how surface condensation can be distinguished from canonical LLPS. We did not address the prewetting transition as such, as others have done this more thoroughly before (referenced by us) and, moreover, our manuscript is already very theory-loaded (distinction of first-order, second-order phase transition etc. will be too much). We provide here a new paragraph and supporting Appendix Figure that we added in response to this request:

Added paragraph:
"Beyond the behaviors that could be directly compared to our experimental observations, our theoretical model can illustrate further differences between a canonical LLPS scenario and surface condensation. To this end, we extracted synthetic microscopy images of the distribution of red particles from simulations without and with a polymer chain that can serve as a condensation surface (Appendix Fig. S12). In the absence of a polymer chain, a dilute and a dense phase could be detected over a range of bulk concentrations of the red particles (Appendix Fig. S12). Within the bulk concentration range in which two phases coexist, the concentration in both phases was approximately constant, despite changes in bulk concentration (gray-shaded region in Appendix Fig. S12). The bulk concentration range of coexistence was extended when the interaction between red particles was made stronger via adjustment of $w_{S5P\text{-}S5P}$ (Appendix Fig. S12). These behaviors are strongly indicative of canonical LLPS (Alberti 2019). Upon addition of a polymer chain, the high concentration phase could already be detected at lower bulk concentrations (additional blue-shaded region in Appendix Fig. S12). Such formation of clusters at sub-saturated bulk concentrations in the

presence of a polymer surface can be used as a clear indicator of surface-mediated condensation. In experiments where the polymer surface cannot be removed or deactivated, one can still see that, in this surface condensation regime, the concentration of the dilute phase increases approximately proportionally with the bulk concentration, despite the presence of a second, dense phase (red line within the blue-shaded regions in Appendix Fig. S12). This analysis indicates two behaviors that distinguish the surface condensation regime from the canonical LLPS regime: (i) cluster formation at sub-saturated bulk concentration and (ii) an increase in dilute phase concentration with bulk phase concentration in the presence of a dense phase.

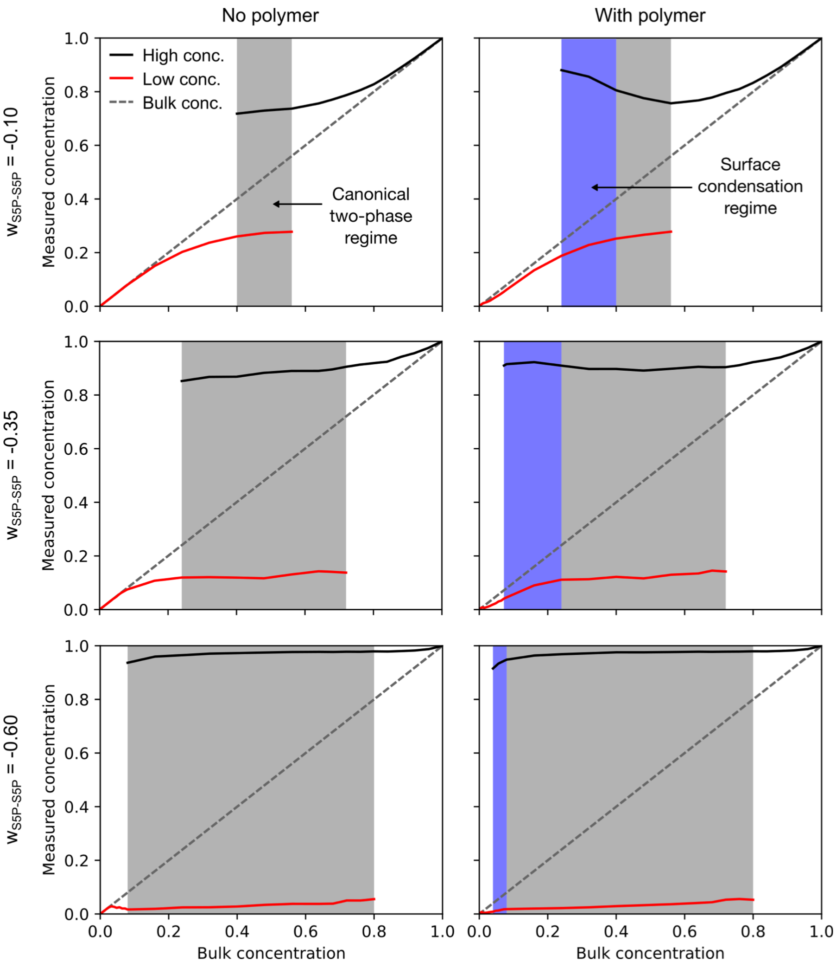

<u>Appendix Figure S12: Presence of low and high concentration phases in lattice model simulations with and without polymer surface.</u> Lattice (25x25) simulations of S5P (N_{S5P} ∈[0,625]) without and with polymer ($L_{polymer}$=20, N_{RC}=8, N_{IC}=12), $w_{RC\text{-}S5P}$=-0.5, and $w_{S5P\text{-}S5P}$=-0.1,-0.35,-0.6 as indicated). Simulation results are processed by applying a Gaussian filter, phase masks are obtained by thresholding (cut-off 0.5) and subsequent erosion of masks with a square structural element of 3x3 pixels. The bulk concentration is indicated for reference (calculated by dividing N_{S5P} by number of total lattice sites). Shaded areas indicate the canonical two-phase regime (gray, obtained empirically from simulations without polymer) and the surface condensation regime (blue, obtained empirically from simulations with polymer).

Finally, the authors claim they have predictions of the model that relate to the perturbations. However, here again they change the parameters of the simulation somewhat arbitrarily (or simply so that it corresponds to the experiments) by for example changing the interaction parameter and particle number in the triptolide perturbation. This is not a prediction.
To be more conservative with our claims, we have changed all language from "predicted" to "reproduced", and also changed "predictive power" to "explanatory power".

Minor comments:
(1) Line 135 - what do you mean by tolerated well? Please explain.
We changed the statement to the following (changes underlined):
"Our previous work demonstrated that fluorescently labeled antigen-binding fragments (Fab) of antibodies against post-translational modifications <u>do not interfere with the normal development in an obvious manner</u> and provide good sensitivity as well as time resolution in zebrafish embryos (Sato 2019, Hilbert 2021)."

(2) I find the reference to an "unfolded" shape to be misleading. Do you mean elongated? Complex geometry? I find that unfolded implies something about the stability of the protein, which I don't think is relevant in this context.
This is a good point. We mean "complex geometry", in the sense that protrusions and invaginations are apparent. We do not mean elongated shape, as an elongated shape can still exhibit a high solidity, in that sense not being unfolded in any apparent way. "Complex morphology", in our opinion, is less specific and idiomatic than "unfolded", which is why we retained the notion of "unfolded shapes". In a few strategic places, we now clarified that we refer to cluster unfolding, to prevent confusion with, for example, protein unfolding. We have refrained from describing cluster shapes with the word "elongation", as it is too difficult to keep this apart from transcriptional elongation.

(3) Hexanediol effect is hard to interpret and the field does not consider this as a proof of phase separation anymore.
We are well aware of the ongoing discussion on hexanediol. We added these experiments in response to the request made by referee 1. We cited several of the papers that point out problems with hexanediol treatment in terms of side effects on catalytic processes, cellular regulation, and genome packing. As far as possible, we tried to work at relatively "safe" concentration and treatment duration, and also tried to carefully choose our words with respect to what can be concluded from these experiments.

Accepted **10th Sep 2021**

Thank you again for sending us your revised manuscript. We think that the performed revisions have satisfactorily addressed the concerns of reviewer #3 and I am therefore pleased to inform you that your paper has been accepted for publication.

EMBO PRESS

YOU MUST COMPLETE ALL CELLS WITH A PINK BACKGROUND ➔

PLEASE NOTE THAT THIS CHECKLIST WILL BE PUBLISHED ALONGSIDE YOUR PAPER

Corresponding Author Name: Lennart Hilbert, Karlsruhe Institute of Technology
Journal Submitted to: Molecular Systems Biology
Manuscript Number: MSB-2021-10272

USEFUL LINKS FOR COMPLETING THIS FORM

http://www.antibodypedia.com
http://1degreebio.org
http://www.equator-network.org/reporting-guidelines/improving-bioscience-research-repor
http://grants.nih.gov/grants/olaw/olaw.htm
http://www.mrc.ac.uk/Ourresearch/Ethicsresearchguidance/Useofanimals/index.htm
http://ClinicalTrials.gov
http://www.consort-statement.org
http://www.consort-statement.org/checklists/view/32-consort/66-title
http://www.equator-network.org/reporting-guidelines/reporting-recommendations-for-tumo
http://datadryad.org
http://figshare.com
http://www.ncbi.nlm.nih.gov/gap
http://www.ebi.ac.uk/ega
http://biomodels.net/
http://biomodels.net/miriam/
http://jjj.biochem.sun.ac.za
http://oba.od.nih.gov/biosecurity/biosecurity_documents.html
http://www.selectagents.gov/

Reporting Checklist For Life Sciences Articles (Rev. June 2017)

This checklist is used to ensure good reporting standards and to improve the reproducibility of published results. These guidelines are consistent with the Principles and Guidelines for Reporting Preclinical Research issued by the NIH in 2014. Please follow the journal's authorship guidelines in preparing your manuscript.

A- Figures
1. Data
The data shown in figures should satisfy the following conditions:

- ➔ the data were obtained and processed according to the field's best practice and are presented to reflect the results of the experiments in an accurate and unbiased manner.
- ➔ figure panels include only data points, measurements or observations that can be compared to each other in a scientifically meaningful way.
- ➔ graphs include clearly labeled error bars for independent experiments and sample sizes. Unless justified, error bars should not be shown for technical replicates.
- ➔ if n< 5, the individual data points from each experiment should be plotted and any statistical test employed should be justified
- ➔ Source Data should be included to report the data underlying graphs. Please follow the guidelines set out in the author ship guidelines on Data Presentation.

2. Captions

Each figure caption should contain the following information, for each panel where they are relevant:

- ➔ a specification of the experimental system investigated (eg cell line, species name).
- ➔ the assay(s) and method(s) used to carry out the reported observations and measurements
- ➔ an explicit mention of the biological and chemical entity(ies) that are being measured.
- ➔ an explicit mention of the biological and chemical entity(ies) that are altered/varied/perturbed in a controlled manner.

- ➔ the exact sample size (n) for each experimental group/condition, given as a number, not a range;
- ➔ a description of the sample collection allowing the reader to understand whether the samples represent technical or biological replicates (including how many animals, litters, cultures, etc.).
- ➔ a statement of how many times the experiment shown was independently replicated in the laboratory.
- ➔ definitions of statistical methods and measures:
  - common tests, such as t-test (please specify whether paired vs. unpaired), simple $\chi 2$ tests, Wilcoxon and Mann-Whitney tests, can be unambiguously identified by name only, but more complex techniques should be described in the methods section;
  - are tests one-sided or two-sided?
  - are there adjustments for multiple comparisons?
  - exact statistical test results, e.g., P values = x but not P values < x;
  - definition of 'center values' as median or average;
  - definition of error bars as s.d. or s.e.m.

Any descriptions too long for the figure legend should be included in the methods section and/or with the source data.

In the pink boxes below, please ensure that the answers to the following questions are reported in the manuscript itself. Every question should be answered. If the question is not relevant to your research, please write NA (non applicable). We encourage you to include a specific subsection in the methods section for statistics, reagents, animal models and human subjects.

B- Statistics and general methods

Please fill out these boxes ➔ (Do not worry if you cannot see all your text once you press return)

1.a. How was the sample size chosen to ensure adequate power to detect a pre-specified effect size?	In all cases where differences were found to be not statstically significant (n.s.), the individual groups contained more than 60 data points. A type II error is thus unlikely except for very small differences. A inumber of 60 data points was readily achieved in our experiments, so that power calculation was omitted.
1.b. For animal studies, include a statement about sample size estimate even if no statistical methods were used.	No animal experiments were included in this study (see below).
2. Describe inclusion/exclusion criteria if samples or animals were excluded from the analysis. Were the criteria pre-established?	Samples were excluded where fluorescence staining failed, all other samples were included.
3. Were any steps taken to minimize the effects of subjective bias when allocating animals/samples to treatment (e.g. randomization procedure)? If yes, please describe.	Where different treatments were applied, fish eggs for the generation of primary cell cultures were chosen randomly from the same batch of eggs.
For animal studies, include a statement about randomization even if no randomization was used.	No animal experiments were included iin this study (see below).
4.a. Were any steps taken to minimize the effects of subjective bias during group allocation or/and when assessing results (e.g. blinding of the investigator)? If yes please describe.	Samples did not need to be grouped after experiments, samples were categorized according to treatment conditions. Analaysis was fully automated, thus inherently preventing investigator bias.
4.b. For animal studies, include a statement about blinding even if no blinding was done	No animal experiments were included iin this study (see below).
5. For every figure, are statistical tests justified as appropriate?	Yes.
Do the data meet the assumptions of the tests (e.g., normal distribution)? Describe any methods used to assess it.	Statistical testing was based on permutation tests throughout. This computational statistics test does not require the assumption of an underlying probability distribution, thus voiding the requirement of a test for normality.
Is there an estimate of variation within each group of data?	Yes. Confidence intervals, standard deviation, standard deviation of mean, or quartile ranges are given with all data shown.

Is the variance similar between the groups that are being statistically compared?	This was not tested, see above point on permutation testing.

C- Reagents

6. To show that antibodies were profiled for use in the system under study (assay and species), provide a citation, catalog number and/or clone number, supplementary information or reference to an antibody validation profile. e.g., Antibodypedia (see link list at top right), 1DegreeBio (see link list at top right).	For antibody validation details, see added PDF file<br>Clone HTA28, H3Ser28Phos, ab10543; Lot # GR3219690-4<br>Clone 4H8, (Pol II CTD phosphoS5), ab5408; Lot # GR205997-15, GR3325973-3
7. Identify the source of cell lines and report if they were recently authenticated (e.g., by STR profiling) and tested for mycoplasma contamination.	The human monocytic cell line (THP- 1) was purchased from DSMZ (Braunschweig, Germany). Authentication is carried out by the supplier, mycoplasma testing is performed locally on a regular basis.

* for all hyperlinks, please see the table at the top right of the document

D- Animal Models

8. Report species, strain, gender, age of animals and genetic modification status where applicable. Please detail housing and husbandry conditions and the source of animals.	Wild type zebrafish (Danio rerio) were used (AB strain, sourced from the Zebrafish International Resource Center and maintained at the European Zebrafish Resource Center). Fish were housed at Karlsrruhe Institute of Technology.
9. For experiments involving live vertebrates, include a statement of compliance with ethical regulations and identify the committee(s) approving the experiments.	Live vertebrates were not used for experimentation. Only eggs of zebrafish were used up to 12 hours post fertilization, so that procedures are not categorized as animal experiments.
10. We recommend consulting the ARRIVE guidelines (see link list at top right) (PLoS Biol. 8(6), e1000412, 2010) to ensure that other relevant aspects of animal studies are adequately reported. See author guidelines, under 'Reporting Guidelines'. See also: NIH (see link list at top right) and MRC (see link list at top right) recommendations. Please confirm compliance.	All zebrafish husbandry was performed in accordance with the EU directive 2010/63/EU and German animal protection standards (Tierschutzgesetz §11, Abs. 1, No. 1) and is under supervision of the government of Baden-Württemberg, Regierungspräsidium Karlsruhe, Germany (Aktenzeichen35-9185.64/BH KIT).

E- Human Subjects

11. Identify the committee(s) approving the study protocol.	N.A.
12. Include a statement confirming that informed consent was obtained from all subjects and that the experiments conformed to the principles set out in the WMA Declaration of Helsinki and the Department of Health and Human Services Belmont Report.	N.A.
13. For publication of patient photos, include a statement confirming that consent to publish was obtained.	N.A.
14. Report any restrictions on the availability (and/or on the use) of human data or samples.	N.A.
15. Report the clinical trial registration number (at ClinicalTrials.gov or equivalent), where applicable.	N.A.
16. For phase II and III randomized controlled trials, please refer to the CONSORT flow diagram (see link list at top right) and submit the CONSORT checklist (see link list at top right) with your submission. See author guidelines, under 'Reporting Guidelines'. Please confirm you have submitted this list.	N.A.
17. For tumor marker prognostic studies, we recommend that you follow the REMARK reporting guidelines (see link list at top right). See author guidelines, under 'Reporting Guidelines'. Please confirm you have followed these guidelines.	N.A.

F- Data Accessibility

18: Provide a "Data Availability" section at the end of the Materials & Methods, listing the accession codes for data generated in this study and deposited in a public database (e.g. RNA-Seq data: Gene Expression Omnibus GSE39462, Proteomics data: PRIDE PXD000208 etc.) Please refer to our author guidelines for 'Data Deposition'.<br><br>Data deposition in a public repository is mandatory for:<br>a. Protein, DNA and RNA sequences<br>b. Macromolecular structures<br>c. Crystallographic data for small molecules<br>d. Functional genomics data<br>e. Proteomics and molecular interactions	All raw data, analysis scripts, and simulation code used in this study are provided for public access via Zenodo under the following DOIs:
19. Deposition is strongly recommended for any datasets that are central and integral to the study; please consider the journal's data policy. If no structured public repository exists for a given data type, we encourage the provision of datasets in the manuscript as a Supplementary Document (see author guidelines under 'Expanded View' or in unstructured repositories such as Dryad (see link list at top right) or Figshare (see link list at top right).	See above
20. Access to human clinical and genomic datasets should be provided with as few restrictions as possible while respecting ethical obligations to the patients and relevant medical and legal issues. If practically possible and compatible with the individual consent agreement used in the study, such data should be deposited in one of the major public access-controlled repositories such as dbGAP (see link list at top right) or EGA (see link list at top right).	N.A.
21. Computational models that are central and integral to a study should be shared without restrictions and provided in a machine-readable form. The relevant accession numbers or links should be provided. When possible, standardized format (SBML, CellML) should be used instead of scripts (e.g. MATLAB). Authors are strongly encouraged to follow the MIRIAM guidelines (see link list at top right) and deposit their model in a public database such as Biomodels (see link list at top right) or JWS Online (see link list at top right). If computer source code is provided with the paper, it should be deposited in a public repository or included in supplementary information.	Simulation code has been provided in the form of scripts deposited on Zenodo.

G- Dual use research of concern

22. Could your study fall under dual use research restrictions? Please check biosecurity documents (see link list at top right) and list of select agents and toxins (APHIS/CDC) (see link list at top right). According to our biosecurity guidelines, provide a statement only if it could.	Our study does not fall under dual use research restrictions.
